# *Meta*-Xylene-Based Diamines with Protected Benzyl Sites: Potential NCN Pincer Ligands with Tunable Steric Profiles

**DOI:** 10.3390/molecules30061331

**Published:** 2025-03-16

**Authors:** Tamina Z. Kirsch, Toren Hynes, Jason D. Masuda, Saurabh S. Chitnis

**Affiliations:** 1 Department of Chemistry, Dalhousie University, 6243 Alumni Crescent, Halifax, NS B3H 4R2, Canada; tamina.kirsch@dal.ca (T.Z.K.);; 2 Department of Chemistry, Saint Mary’s University, 923 Robie Street, Halifax, NS B3H 3C3, Canada

**Keywords:** pincer ligands, coordination chemistry, bismuth, structure

## Abstract

Bulky NCN aryl-diamides featuring methyl groups in the benzyl positions were synthesized with the aim of creating a new class of *meta*-xylene-based trianionic pincer ligands where the common decomposition pathway of metal pincer complexes via C-H activation is prevented. Sterically demanding substituents on the ligands furthermore provide steric protection of the metal centre and can help prevent the dimerization of the complexes. While a double deprotonation of the ligands and the formation of a dilithium salt was straightforward, difficulties were encountered when attempting to deprotonate the *ipso*-C*H* proton on the central aryl ring to yield trianionic ligands. This stands in contrast to related pincer ligands without methyl groups in the benzylic positions. Experimental and theoretical investigations led to the conclusion that the challenges encountered when attempting the third deprotonation are likely caused by an interplay of increased electron density at the nitrogen atoms and steric hindrance. Both effects originate in the introduction of methyl groups in the benzylic positions, which make the targeted proton less accessible. These results provide further insight into the impact of methyl groups in the benzyl positions on both steric and electronic properties of NCN pincer ligands, which may find utility in coordination chemistry applications where metalation can be achieved by direct C-H activation rather than requiring triple deprotonation.

## 1. Introduction

Pincer ligands are tridentate ligands with a preference for meridional coordination around a metal centre [1]. The term was first introduced by van Koten, who highlighted their unique stereochemistry and high stability [2]. The scope of pincer ligand coordination now encompasses a wide variety of metals, nonmetals and metalloids [2,3,4]. Their versatility and ability to tune and influence metal centres have made them the cornerstone for many processes of modern-day catalysis, like dehydrogenations, hydrogenation reactions and cross-coupling reactions. Several reviews published on this topic highlight the historic and recent advantages in this field [5,6,7,8,9].

While meridional coordination has historically been studied for transition metals, there is rising interest in the application of pincer ligands for group 13–15 elements [5,6,7,8,9]. This is because pincer ligands can yield access to unusual (e.g., non-VSEPR) geometries and low oxidation states [10]. Select examples, focusing on group 15, include the isolation of T-shaped pnictogens centres and the isolation of monomeric pnictinidenes stabilized by monoanionic diamine ligands [11,12,13,14,15].

**Figure 1:**
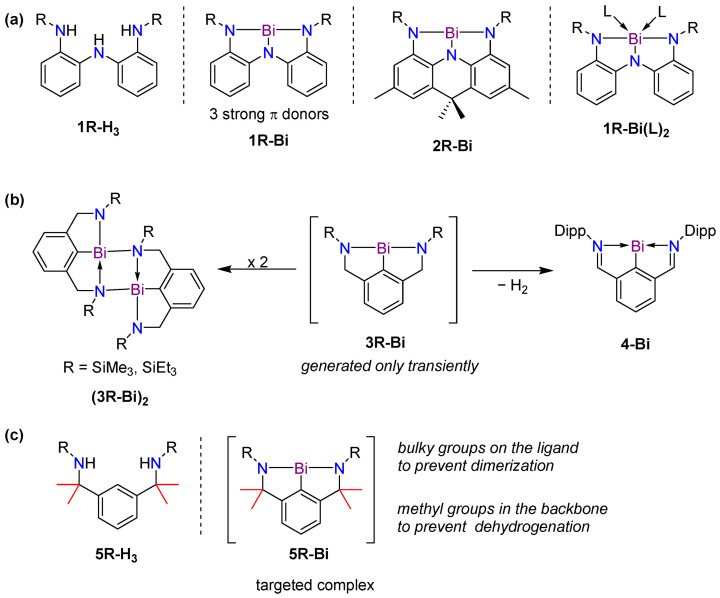
(**a**) Triamide-based T-shaped bismuth pincer complexes. (**b**) Previously targeted arylamide bismuth pincer complexes and formation of bimetallic complexes (left) or monomeric bismuthinidenes via dehydrogenative reduction (right). (**c**) New ligands and complexes targeted in this work.

**Figure 2:**
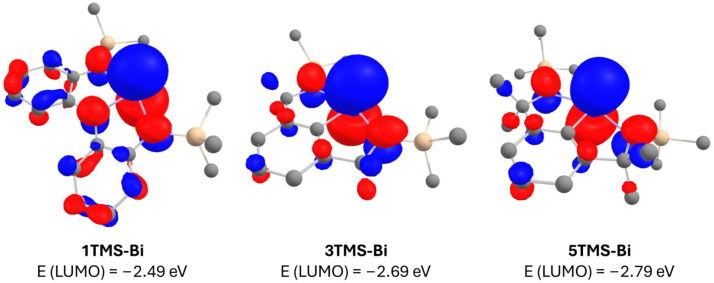
Visualization of the LUMOs (isovalue = 0.036) localized at bismuth for T-shaped bismuth pincer complexes **1R-Bi**, **3R-Bi** and **5R-Bi** and their respective calculated LUMO energies at the PBE0/def2-TZVP level. SiMe_3_ groups were selected as substituents on the flanking N atoms in each case for better comparison. Hydrogen atoms have been omitted for clarity.

We have applied a family of trianionic NNN pincer ligands, **1R-H_3_**, for the isolation of T-shaped planar bismuth complexes **1R-Bi** (Figure 1a) [16,17]. These show a high Lewis acidity due to a partially vacant p-type orbital at bismuth, which enables coordination to give derivatives of **1R-Bi(L)_2_**. The Lewis acidity has prompted the prediction of their use in metallopolymer synthesis [18] and led to a successful application as catalysts for the ring-opening polymerization of dilactide and ε-caprolactone [19]. The presence of three strongly π-donating nitrogen atoms stabilizes the planar structure at the metal, which can also exhibit nucleophilic reactivity, as shown recently for **2R-Bi** [17]. In an attempt to attenuate the ligand to metal electron donation, aryldiamide-based ligands were also explored, as these feature only two strongly π-donating nitrogen atoms and one weakly π-donating aryl group (Figure 1b, **3R-Bi**) [20]. Although the targeted complexes could be detected as monomeric species in the gas phase using ESI mass spectrometry, the species were only isolable as dimers both in solution and in the solid state. In order to prevent dimerization, ligands with larger substituents (e.g., 2,6-diisopropylphenyl, Dipp) at the nitrogen atoms were used. However, in this case, instead of the desired monomeric species, the formation of a bismuthinidene complex, **4-Bi**, was observed via double benzyl C-H activation and dehydrogenation. Thus, a new arylamide-based ligand framework, **5R-H_3_**, was designed (Figure 1c), where bulky aryl substituents at nitrogen would suppress dimerization while the methyl groups in the backbone would preclude benzyl dehydrogenation of the ligand. DFT calculations predicted complexes **5R-Bi** to be very similar in LUMO energy compared to complexes **3R-Bi,** with the LUMO being heavily localized at the bismuth centre as a p-type vacant orbital (Figure 2). This makes complexes **5R-Bi** attractive synthetic targets as potentially very strong Lewis acids.

In this paper, we describe the synthesis, steric properties and characterization of a new class of bulky trianionic NCN pincer ligands **5R-H_3_** featuring methyl groups in the ligand side arms. Although our intended exploration of planar bismuth chemistry within this ligand framework (**5R-Bi**) was ultimately not successful due to the inability to achieve the necessary threefold deprotonation, we show that they are an easily tunable class of ligands that offer higher steric protection than their predecessors, showing that remote substitution can have a significant influence in the coordination pocket. We envision that these ligands might open new coordination opportunities for other elements, including transition metals, where metalation can be achieved without triple deprotonation (i.e., via direct aryl C-H activation). The protection of the benzyl sites via methylation precludes side reactions, which have previously challenged the use of such ligands at transition metal centres [21,22,23,24,25,26,27].

## 2. Results and Discussion

### 2.1. Synthesis

**Figure 3:**
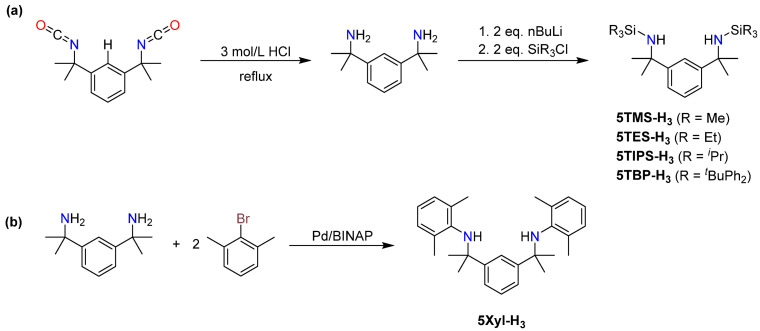
(**a**) Synthesis of silyl-substituted compounds **5R-H_3_** (R = TMS; TES; TIPS; TBP). (**b**) Synthesis of xylyl-substituted compound **5Xyl-H_3_**.

Precursor α^1^,α^1^,α^3^,α^3^-tetramethyl-1,3-benzenedimethanamine was made from commercially available 1,3-bis(1-isocyanato-1-methylethyl)benzene in 90% yield (Figure 3a). This diamine has also been applied successfully as a monoanionic pincer ligand toward palladium [28]. The installation of the silyl substituents in compounds **5R-H_3_** was performed by lithiation of the diamine, followed by in situ salt metathesis reaction with the corresponding silyl chlorides. Despite containing a polar Si-N bond, **5TIPS-H_3_** and **5TBP-H_3_** were found to be air- and moisture-stable and could be recrystallized from ethanol. The synthesis of aryldiamine **5Xyl-H_3_** was realized via a Buchwald–Hartwig amination; **5Xyl-H_3_** was obtained in >90% yield (Figure 3b). The synthesis of an even bulkier, Trip-substituted aryldiamine compound (**5Trip-H_3_**) was attempted, but although its formation could be confirmed via mass spectrometry and NMR spectroscopy, the obtained yields were too low to allow for a proper purification and analysis.

**Figure 4:**
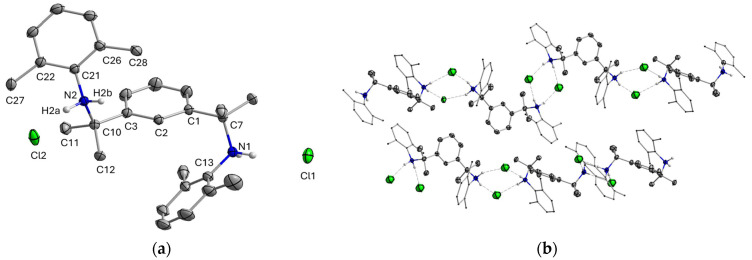
(**a**) Views of the molecular structure of **5Xyl-H_3_·2HCl** in the solid state. (**b**) Intermolecular hydrogen bonding interactions in the crystal lattice. Non-essential hydrogen atoms and solvent molecules have been omitted. Thermal ellipsoids are drawn at the 50% probability level.

Silylamines **5TMS-H_3_**, **5TES-H_3_** and arylamine **5Xyl-H_3_** were isolated as oils. As known from the literature, closely related molecules without the methyl-armed backbone were also isolated as oils [20,29]; this characteristic can be attributed to the bulky substituents at N that prevent efficient packing for a solid-state structure rather than an influence of the methyl groups. As expected, compounds **5TIPS-H_3_** and **5TBP-H_3,_** whose molecular weight exceeds 500 g/mol, were isolated as solids. Crystals suitable for single-crystal X-ray diffraction of the hydrochloride salt **5Xyl-H_3_·2HCl** were obtained by adding a few drops of concentrated hydrochloric acid to a solution of **5Xyl-H_3_** in ethanol after 2 weeks at −18 °C. The solid-state structure of this compound is displayed in Figure 4. The unit cell contains one-half molecule of ethanol, which has been omitted in Figure 4 for clarity. The selected bond lengths and angles are listed in Table 1.

In the solid state, the two side arms attached to the central aromatic ring in **5Xyl-H_3_** are significantly twisted with a C-N-C-C torsion angle of 50.90 (9)° (C13-N1-C7-C1) and 63.09 (8)° (C3-C10-N2-C21), respectively, between the Xylyl substituent and the central aryl ring. Although the steric influence of the methyl groups on the benzyl side arm cannot be excluded, this is most likely caused by the strong interaction between the NH_2_^+^ groups and the Cl^−^ ions. The distance between the chlorine ions and the nitrogen atoms with a range of 3.1328 (8)—3.1358 (8) Å lies well within the sum of their respective van der Waals radii of 3.30 Å [30]; the distance of the chlorine ion to the closest hydrogen atom bound to nitrogen, measured at 2.26 (1) Å, further supports the presence of hydrogen bonding [31]. Each chlorine ion acts as a twofold hydrogen bond acceptor between two different molecules of **5Xyl-H_3,_** which leads to the formation of the one-dimensional network (Figure 4b).

### 2.2. Steric Impact of the Methyl-Substituted Ligand Arms

The impact of incorporating methyl groups into ligand arms in replacement of hydrogen substituents has been studied in detail by Khusnutdinova and co-workers for neutral pyridine-based PNP ligands, which are commonly used in transition metal catalysis [32]. The substitution of the hydrogen substituents on the ligand arms with either one or two methyl groups resulted in higher catalytic activity in certain cases, such as the dehydrogenation of alcohols and altered catalyst selectivity [33]. For example, while the CH_2_-armed complex catalyzed the dimerization of the alkynes [34], the tetramethyl-substituted version catalyzed selective semi-hydrogenation to alkenes [35,36]. The usage of benzyl CHMe-armed complexes where only one hydrogen atom was substituted with a methyl group led to over-hydrogenation to ethylbenzene, highlighting once more the impact of even small steric modifications in sidearms of pincer complexes. The observed selectivity is reasoned to be caused by steric repulsion within the formed reaction intermediate, which is caused by the methyl groups in the ligand side arms. In light of these observations, we sought to investigate the steric properties of the ligands prepared here via comparison with the non-methylated analogues prepared earlier.

The buried volume percentage (%V_bur_) of the DFT-optimized structures of the corresponding bismuth complexes, **3R-Bi** and **5R-Bi**, were calculated using SambVca 2.1 [37,38,39]. The ligands can be grouped into aryl-substituted and silyl-substituted, and calculations were performed with one member of each group by computationally modelling the structure of the corresponding bismuth complex (**3R-Bi** or **5R-Bi**). As we have reported previously [40], when performing energy scans of the dihedral angle between the central bismuth atom and its substituents, the preference of bismuth aryldiamine complexes for a planar geometry is quite small, with less than 20 kJ/mol, compared to a dihedral angle closer to 120°. Based on these results, a fluxional behaviour of the complexes might be observed in solution. However, to allow for an easier comparison with related well-researched planar triamide complexes (Figure 1a), only the planar configuration of the complexes was further investigated.

**Figure 5:**
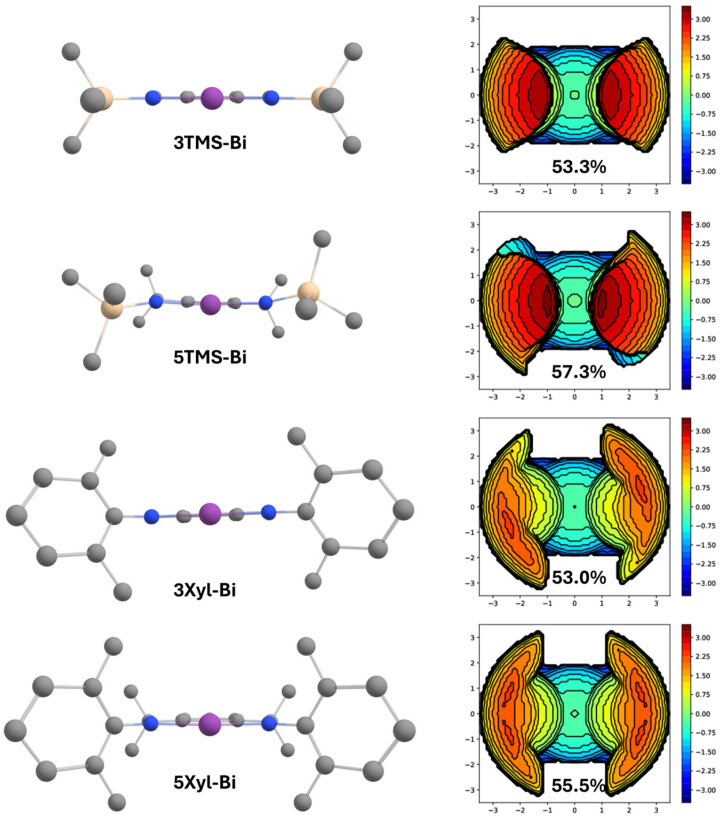
(**Left**): Calculated structures for compounds **3TMS-Bi**, **5TMS-Bi**, **3Xyl-Bi** and **5Xyl-Bi** (top to bottom) Hydrogen atoms have been omitted for clarity. Grey: carbon; blue: nitrogen; purple: bismuth. (**Right**): Visualization of the respective steric maps.

The optimized structures and the respective steric maps are displayed in Figure 5. The steric maps show that the xylyl and silyl substituents provide comparable levels of steric protection, with %V_bur_ values within each ligand class being only slightly higher for the silyl derivatives. Interestingly, there is a pronounced difference between the distribution of steric bulk between the xylyl and silyl substituents, with the former offering the most steric shielding farther away from the metal and the latter concentrating the steric protection in close vicinity of the metal. Importantly, the steric maps highlight that the substitution of methyl groups at the ligand side arms leads to an increased steric shielding of the binding pocket (by 3–5%). While this increased steric shielding is desirable as it should help prevent the formation of dimeric complexes via coordination of the central bismuth atom, it also might potentially limit the access towards the *ipso*-C*H* atom of the ligand precursor, therefore reducing its reactivity. This assumption was confirmed experimentally when all attempts of activating the targeted proton in order to synthesize **5R-Bi** were met with a great challenge, which will be discussed in detail in the following section.

### 2.3. Attempted Synthesis of ***5R-Bi***

**Figure 6:**
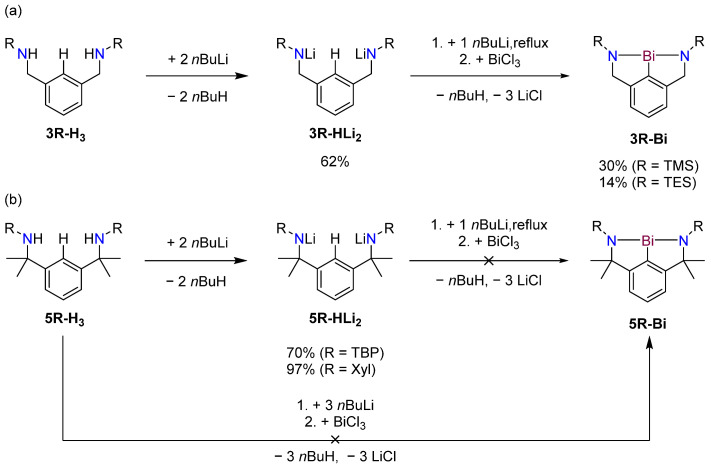
Reaction scheme to synthesize complexes **(3R-Bi)_2_** and **5R-Bi**. (**a**) Successful synthesis of complexes **(3R-Bi)_2_** via **3R-HLi_2_** as previously reported [10]; (**b**) attempted synthesis of **5R-Bi**. Top: Stepwise lithiation to **5R-Li_3_** via **5R-HLi_2_**, followed by salt metathesis using BiCl_3_. Bottom: One-pot synthesis starting from ligand **5R-H_3_**.

The synthesis of complexes **5R-Bi** was attempted via one-pot triple lithiation of **5R-H_3_**, followed by salt metathesis using BiCl_3_ (Figure 6), in line with the approach that was previously used successfully to synthesize the related bismuth complex **3R-Bi**, isolated as **(3R-Bi)_2_** [16,20]. Despite several attempts involving variation in solvents, temperatures and organolithium reagents used for deprotonation, this approach consistently yielded only bismuth metal and partially protonated ligands following workup.

Further experimentation using **5Xyl-H_3_** as a representative model revealed more information about these synthetic challenges. Attempts to deprotonate **5Xyl-H_3_** using NaO*t*Bu or LiHMDS were unsuccessful. In both cases, no reaction was observed, and the ligand was recovered as an orange oil. Using *n*BuLi, however, doubly deprotonated **5Xyl-HLi_2_** was obtained as a yellow powder that did not require any further purification after solvent removal (Figures S1 and S2). The compound **5Xyl-HLi_2_** is only poorly soluble in non-coordinating solvents such as benzene or toluene at room temperature. NMR spectra measured in C_6_D_6_ displayed multiple broad peaks that could not be assigned easily even though an NMR spectrum of the same batch of **5Xyl-HLi_2_**, measured in THF, displayed sharp peaks without any visible impurities (Figures S2 and S3). This hints towards the aggregation of in non-coordinating solvents, explaining both the broad signals and the poor solubility, which lies in agreement with reports by Zdanski and co-workers [29] and Veige and co-workers [41], respectively, who reported the formation of dimers for the related salts **3Xyl-HLi_2_** and **3Xyl-Li_3_**. Accordingly, most reactivity studies of **5Xyl-HLi_2_** were performed in THF to ensure disaggregation in solution.

With the doubly deprotonated compound in hand, several attempts were made to achieve the final deprotonation (Table 2). In all cases, NMR assays revealed that either no reaction had taken place or extensive decomposition of either the organolithium or **5Xyl-HLi_2_** was observed. For example, the ^1^H NMR spectrum of the reaction mixture containing **5Xyl-HLi_2_** and *n*BuLi is shown in Figure S5. After heating the reaction mixture to 65 °C for 30 min (entry 2), the peak assigned to *n*BuLi disappeared, but no change in chemical shift for the signals of **5Xyl-HLi_2_** occurred (Figure S6). A second set of minor signals next to the signals assigned to **5Xyl-HLi_2_** in the aromatic region appeared, which might be due to aggregation of the dilithium salt **5Xyl-HLi_2_** or decomposition. No evidence of **5Hy-H_3_** was observed (see Figure S7), precluding hydrolysis. Using the more basic *t*BuLi (entry 4) also did not yield the fully deprotonated ligand. A ^1^H NMR spectrum of the reaction mixture only showed signals corresponding to salt **5Xyl-HLi_2_** (Figure S9). The reaction between **5Xyl-HLi_2_** and *sec*BuLi (entry 7) also did not yield the targeted compound **5Xyl-Li_3_**, as confirmed by ^1^H NMR spectroscopy (Figure S17) and indirectly by ^2^H NMR spectroscopy after quenching with MeOD-*d*_4_ (Figure S18).

The observed difficulties in removing the third proton lie in accordance with the reports by Veige and co-workers [41], who stated that the removal of the third proton of their closely related ligand **3Dipp** was only possible under forcing conditions by refluxing the ligand in toluene with 3.5 eq. MeLi for 45 min. Accordingly, harsher conditions were explored in order to access **5Xyl-Li_3_**. After refluxing **5Xyl-HLi_2_** with 1.2 eq *n*BuLi in toluene for 30 min (entry 5), the formation of a dark red solution was observed. However, analysis using NMR spectroscopy revealed that the major product was still unreacted **5Xyl-HLi_2_** (Figure S10).

**Figure 7:**
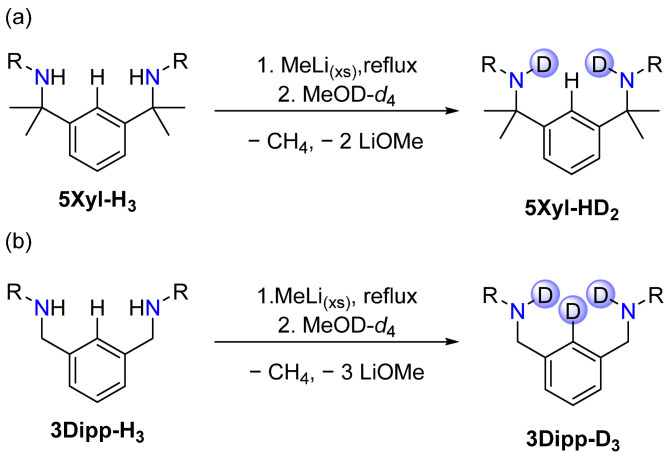
(**a**) Quenching experiment to test for the formation of **5R-Li_3_** using MeOD-*d*_4_ and detection of **5Xyl-HD_2_**. (**b**) Control experiment using **3Dipp-H_3_** [41] under the same conditions and observed formation of **3Dipp-D_3_**.

Increasing the reflux time to 1 h while using 1.2 eq. MeLi as the lithiating reagent (entry 6) gave inconclusive results as the ^1^H NMR spectrum of the reaction solution featured a very crowded aromatic region, potentially due to clustering or due to the formation of a mixture of products (Figure S11). Some signals could be assigned to the compound **5Xyl-H_3_**, most likely due to partial decomposition of the NMR sample before the measurement occurred. To exclude the possibility that **5Xyl-Li_3_**, which was anticipated to be highly moisture sensitive, in fact formed and then decomposed during the workup of the reaction, a quenching experiment using MeOD-*d*_4_ was performed (Figure 7a).

After refluxing **5Xyl-HLi_2_** with 1.2 eq. MeLi in toluene for 1 h, excess MeOD-*d*_4_ was added to the reaction mixture, which was accompanied by heat development and a colour change from dark red to orange. All solvents were removed under a vacuum, and a ^2^H NMR sample in protonated THF was subsequently obtained. The spectrum displayed multiple peaks between 1.97 ppm and 4.00 ppm but, notably, no peak in the aromatic region, which confirms that the third lithiation targeting the *ortho*-C*H* was unsuccessful (Figure S12). The major peak at 3.47 ppm was assigned to the N*D* in accordance with the observed chemical shift in the corresponding N*H* signal in the ^1^H NMR spectrum. Two peaks at 3.58 ppm and 1.73 ppm, respectively, correspond to THF-*d,* and the remaining peaks at 4.00 ppm, 3.18 ppm and 1.97 ppm most likely correspond to residual MeOD-*d*_4_ and LiOMeD in the sample.

To confirm the validity of the quenching experiment, the same conditions were applied to a sample of **3Dipp** using MeLi and MeOD-*d*_4_ (Figure 7b). A control NMR sample taken before adding MeOD-*d*_4_ confirmed the formation of **3Dipp-Li_3_** as the peaks detected in the ^1^H NMR spectrum match the reported spectrum for **3Dipp-Li_3_** (Figure S15) [41]. The ^2^H NMR spectrum obtained after quenching the reaction mixture containing **3Dipp-Li_3_** with MeOD-*d*_4_ showed only two signals, as expected (Figure S16). In combination with the ^1^H NMR spectrum, which confirmed the formation of **3Dipp-Li_3_** prior to quenching, it can be concluded that the signal at 7.57 ppm corresponds to the previously lithiated *ortho*-C*D* position, while the signal at 3.11 ppm can be assigned to the two N*D* groups. This serves as a confirmation that the quenching experiment is in fact, a valid method to indirectly detect the formation of the targeted lithium salts.

**Figure 8:**
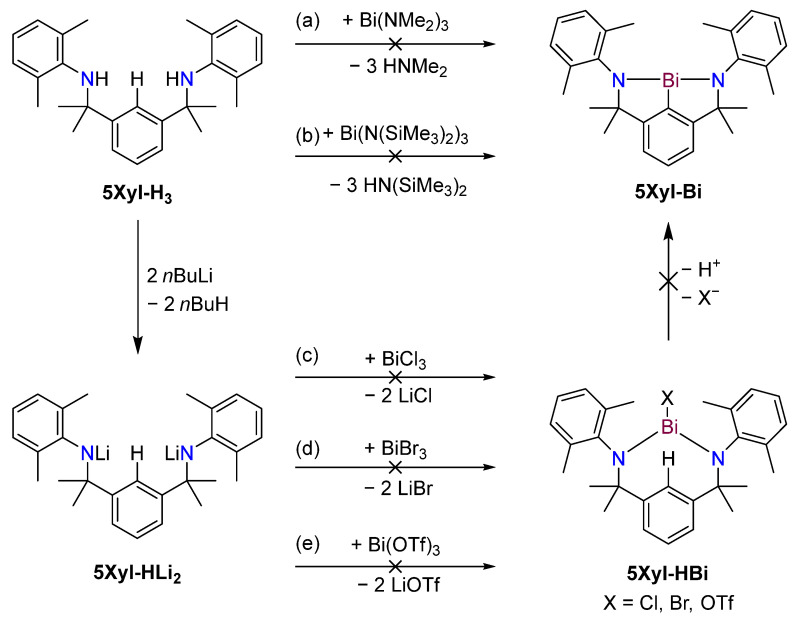
Reactions of with bismuth precursors to access **5Xyl-Bi**, starting from **5Xyl-H_3_** (**top**) or **5Xyl-HLi_2_** (**bottom**).

As the method has previously been applied successfully to access a variety of planar bismuth triamides [17,19,41], direct deamination between **5Xyl-H_3_** and Bi(NMe_2_)_3_ was also investigated (Figure 8a). No reaction was observed between **5Xyl-H_3_** and Bi(NMe_2_)_3_ or Bi(N(SiMe_3_)_2_)_3_, even with catalytic amounts of pyridine and stirring over multiple days in the 110–150 °C range (Figures S19–S21). Besides reacting **5Xyl-H_3_** with bismuth amides, the reactivity of **5Xyl-HLi_2_** was also investigated with bismuth (pseudo)halides with the aim of preparing a bismuth-containing complex, **5Xyl-HBi**, which retained an *ipso*-C*H*. We postulated that the proximity of the bismuth atom to the *ipso*-C*H* should increase the acidity of the latter and therefore help remove the third proton to yield **5Xyl-Bi**. However, although a reaction could be observed with all three bismuth precursors (Figure 8c–e), it was unselective, and attempts to isolate the targeted compound were not successful. Over time, the continuous formation of a very dark precipitate was observed, even after extracting the reaction mixture after solvent removal with non-coordinating solvents, such as toluene or hexanes, to separate it from the byproduct salts. Upon air exposure, the precipitate turned off-white, therefore excluding the hypothesis that it might be exclusively bismuth metal (Figure S25). Similar results were obtained when using BiBr_3_ as a precursor (route d, Figure 8). Addition led to an immediate colour change to dark green and the formation of a dark precipitate. The crowded NMR spectrum might either hint towards an unselective or incomplete reaction or clustering of the potential product **5Xyl-HBiBr** (Figure S27). Unfortunately, a clean isolation of **5Xyl-HBiBr** failed once again. Experiments involving silylamine compounds **5R-H_3_** gave results similar to those observed for **5Xyl-H_3_**. While the isolation of the dilithium salts was straightforward, attempts to access **5R-Bi** via a third lithiation and reaction with BiCl_3_ appeared to be unsuccessful. The reaction between **5R-HLi_2_**, 1.1 eq. MeLi and 1 eq. BiBr_3_ led to the formation of a green-brown solution. The NMR spectrum supports the hypothesis of the formation of a dimeric **5R-HBiBr** species, displaying two sets of signals (Figure S29).

### 2.4. Impact of the Methyl Side Arms on Deprotonation

The observed challenges associated with performing the third deprotonation on **5R-HLi_2_** in comparison to **3R-HLi_2_** can have their cause either in steric or electronic effects.

The chemical shift in the signals in the ^1^H NMR spectrum can be used as a rough indicator to compare the acidity of protons within the same chemical environment [42,43]. The chemical shift for the *ipso*-C*H* in **3Dipp-H_3_** is 7.45 ppm in CDCl_3_ [29], which is in agreement with the detected aromatic signal in the ^2^H NMR spectrum after performing a quenching experiment of the corresponding trilithium salt. By comparison, the signal appears almost 0.5 ppm upfield of the *ipso*-C*H* signal of **5Xyl-H_3_**. Assuming a correlation between the electronic deshielding and acidity of the C*H* proton, as is the case for classic protic hydrogen atoms and their hydrogen bond acidity [43], this would hint towards a higher acidity of the *ipso*-C*H* proton on **5Xyl-H_3_** compared to **3Xyl-H_3_**, which is inconsistent with the observed reactivity. We speculate that the greater steric bulk perturbs the chemical shifts to a larger extent than electronic effects in this case, preventing this approach to estimating acidity.

We also considered that the *ortho*-directing effect of the two amine side arms of **5R-HLi_2_** might not have been predominant enough to exclusively yield *ortho*-lithiated products **5R-Li_3_** and that a deprotonation might have occurred on the methyl groups of the ligand side arms instead. However, the NMR data obtained from the deuteration experiments after lithiation make it seem unlikely that a lithiation in said position occurred in more than trace amounts since the main signals obtained in the NMR spectrum perfectly match the unreacted compound **5R-H_3_**.

While studies of the pk_a_ values of amine-substituted benzene derivatives are less common, most likely due to the lower acidity of aromatic protons compared to amines, there are several reports about the impact of substituents on the pk_a_ values of protonated pyridine derivates that provide some guidance of the effect of steric bulk on acidity. For example, Lefebvre and co-workers performed an extensive experimental and computational study, investigating the acidity of 2,6-di-tert-butylpyridine in comparison to pyridine and other *ortho*-substituted pyridine derivates [44]. In both aqueous solution and dimethyl sulfonamide (DMSO), the acidity decreased in the order pyridine-H^+^ > 2-methylpyridine-H^+^ > 2,6-dimethylpyridine-H^+^ (pK_a_ = 5.17, 5.97, 6.75 (water); = 3.45, 4.01, 4.46 (DMSO)). These results support the hypothesis that the acidity decreases with increasing substitution in proximity to the acidic centre, which can be reasoned to be caused both by the positive inductive effects of the methyl groups and the additional steric shielding, which might limit the access of the base.

**Figure 9:**
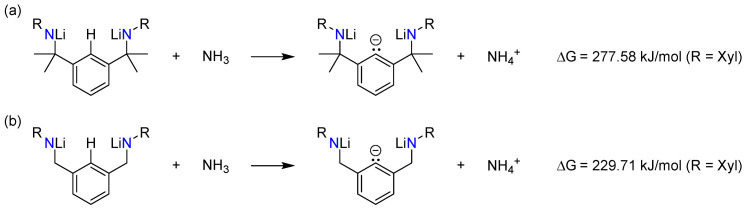
Calculated proton transfers from **5Xyl-HLi_2_** (**a**) and **3Xyl-HLi_2_** (**b**) to NH_3_ using a PCM model (THF) for solvation.

This hypothesis was also borne out by our DFT calculations involving the proton transfer from **5Xyl-HLi_2_** or **3Xyl-HLi_2_** to NH_3_. To model the impact of solvation, an implicit solvent model was applied using thf as the solvent. Proton transfer was more favourable in the case of **5Xyl-HLi_2_** (Figure 9). The absolute values of the energies must be considered with caution as the calculations were performed in the gas phase without the consideration of intermolecular interactions, which are anticipated to be very strong as lithium amides are known to form aggregates even in solution [45]. The calculated Gibbs free energies suggest that the removal of the *ipso*-C*H* proton is less favoured by almost 50 kJ/mol for **5Xyl-HLi_2_** compared to the equivalent **3Xyl-HLi_2_** without benzylic methyl groups. This thermodynamic preference can be explained by the fact that the methyl groups inductively increase the electron density at the nitrogen atoms, making the removal of the proton, which creates a negative charge in very close proximity, less favourable.

Together with a reduced acidity, the increased steric shielding of the central *ipso*-C*H* proton in **5Xyl-HLi_2_** compared to **3Xyl-HLi_2_** should require a greater deformation energy penalty of the ligand towards an approaching base and therefore pose a higher activation barrier for final deprotonation.

Thus, the challenging deprotonation of derivatives of **5R-HLi_2_** is likely caused by an interplay of both electronic factors (increased electron density due to the methyl groups) and steric hindrance, which makes the targeted proton less accessible, rendering the formation of **5R-Li_3_** unfavourable under the range of conditions attempted.

## 3. Materials and Methods

### 3.1. General Synthetic Procedures

All manipulations were performed using standard Schlenk and glovebox techniques under an atmosphere of dry nitrogen. Solvents were distilled from Na/benzophenone and stored over molecular sieves prior to use. Deuterated benzene was freeze-pump-thawed twice and stored over activated 3 Å molecular sieves for at least 48 h. Reaction glassware was baked in a 130 °C oven for at least 1 h prior to use and assembled under nitrogen or pumped into a glovebox while hot. Nuclear magnetic resonance spectra are referenced to tetramethylsilane (^1^H, ^13^C) on a Bruker AVANCE 300 spectrometer, Bruker AVANCE Neo 400 spectrometer or a Bruker AVANCE Neo 500 spectrometer (Bruker, Ettlingen, Germany), with residual solvent used for chemical shift calibration. The spectra are referenced to tetramethylsilane. ^2^H NMR spectra were measured on a Bruker AV-400 spectrometer using proteo-solvents and calibrated using residual ^2^H solvent signals of the respective solvents. Samples for NMR spectroscopy were prepared and sealed inside the glovebox with Parafilm before removal into ambient atmosphere. Electronspray Ionization (ESI) and Atmospheric Pressure Chemical Ionization (APCI) spectra were obtained on a Bruker micrOTOF instrument. Elemental analyses were performed using samples packaged in tin boats under air. Combustion analysis was performed using an Elementar Unicube instrument in CHN/S mode. Chlorotrimethylsilane (TMSCl), chlorotriethylsilane (TESCl), chlorotriisopropylsilane (TIPSCl) and *tert*-butyldiphenylchlorosilane (TBPCl) were purchased from TCI America and used as received. *n*BuLi solution and 1,3-bis(1-isocyanato-1-methylethyl)benzene were purchased from Millipore Sigma and used as received. The unsubstituted diamine precursor was prepared by refluxing 1,3-bis(1-isocyanato-1-methylethyl)benzene in 3 m aqueous hydrochloric acid following modified literature procedures [46].

### 3.2. X-Ray Crystallography

The crystal chosen was attached to the tip of a MicroLoop with Paratone-N oil. Measurements were made on a Bruker D8 VENTURE diffractometer equipped with a PHOTON III CMOS detector using monochromated Mo Kα radiation (λ = 0.71073 Å) from an Incoatec micro-focus sealed tube at 150 K. The initial orientation and unit cell were indexed using a least-squares analysis of the reflections collected from a complete 180 phi-scan with 1 per frame. For data collection, a strategy was calculated to maximize data completeness and multiplicity in a reasonable amount of time and then implemented using the Bruker Apex 4 software suite [47]. Data collection, unit cell refinement, data processing and multi-scan absorption correction were applied using the APEX4 [47] software package (version V2022.10-0.). The structure was solved using SHELXT [48], and all non-hydrogen atoms were refined anisotropically with SHLEXL [49] using the OLEX2 [50] graphical user interface. Unless otherwise noted, all hydrogen atom positions were idealized and ride on the atom to which they were attached. The final refinement included anisotropic displacement factors on all non-hydrogen atoms. CCDC 2423437 contains the supplementary crystallographic data for this paper. These data can be obtained free of charge via https://www.ccdc.cam.ac.uk/structures/ (accessed on 11 March 2025).

### 3.3. Theoretical Calculations

All calculations were performed using the Gaussian 16 Suite [51]. The PBE0 hybrid functional (with D3BJ dispersion correction) and the def2-SVP basis set (with associated pseudopotentials) were used in all cases [52]. In all cases, vibrational frequency calculations showed zero negative frequencies for the optimized structures.

The percentage of buried volume %V_bur_ was calculated based on the coordinates of the DFT-optimized geometry of the complexes using the webtool SambVca 2.1 [37,38,39]. Bondi radii were scaled by 1.17, and the sphere radius was set to R = 3.5 Å for all calculations. Hydrogen atoms were not included in the calculations.

## 4. Conclusions

We report the synthesis of new NCN pincer aryl-diamides whose steric profiles can be modularly varied via Si-N or C-N coupling reactions. Unlike previous benzylamine-based pincers, the ligands revealed here feature methyl groups in the benzyl positions, which preclude benzyl C-H activation-based decomposition pathways. The introduction of methyl groups in the benzyl position also increases the steric protection afforded to coordinated metals, as shown computationally by comparing %V_bur_ values of the hypothetical complexes **3R-Bi** and **5R-Bi**. However, attempts to achieve the triple deprotonation and metalation of the ligands were comprehensively foiled, consistently yielding the doubly deprotonated salts **5R-HLi_2_**, which is in stark contrast to the facile triple deprotonation of the ligand **3R-H_3_** to give **3R-Li_3_**. A combination of experimental and computational methods suggests that the inability to remove the third proton is due to a combination of electronic and steric effects. These results provide further insight into the stereoelectronic variation in NCN pincer ligands. We anticipate that although ligand metalation was not achieved in the present case, the reported compounds may nevertheless prove useful when metalation may be achievable via direct C-H activation (e.g., in transition metals) rather than salt metathesis. Moreover, there is no obvious reason to believe that derivatives of **5R-Bi** are intrinsically inaccessible, and other synthetic approaches (e.g., lithium halogen exchange instead of deprotonation) may prove fruitful in their pursuit.

## Figures and Tables

**Table 1:** Selected bond lengths and angles of compound **5Xyl-H_3_·2HCl**.

Bond Lengths and Angles	[Å] and [°]
C1-C2	1.400 (1)
C2-C3	1.397 (1)
C3-C10	1.525 (1)
C10-C12	1.525 (1)
C10-C11	1.531 (1)
C10-N2	1.562 (1)
N2-C21	1.480 (1)
C21-C26	1.406 (1)
C26-C28	1.508 (1)
C21-C22	1.408 (1)
C22-C27	1.509 (1)
C1-C7	1.5222 (1)
C7-N1	1.556 (1)
C3-C10-N2	108.83 (6)
C1-C7-N1	111.13 (6)
C10-N2-C21	118.51 (6)
C7-N1-C13	119.92 (6)
C11-C10-C12	108.32 (7)

**Table 2:** Reagents and conditions used to synthesize the triple lithiated salt **5Xyl-Li_3_** starting from **5Xyl-HLi_2_**.

Entry	Reagent	Solvent	Condition
1	*n*BuLi	THF	r.t.
2	*n*BuLi	THF	60 °C
3	*n*BuLi	THF	60 °C, +TMEDA
4	*t*BuLi	THF	−30 °C to r.t.
5	*n*BuLi	toluene	30 min at 110 °C
6	MeLi	toluene	1 h at 120 °C
7	*sec*BuLi	THF	−78 °C to r.t.

## Data Availability

Data are contained within the article and Supplementary Materials.

## Supplementary Materials

The following supporting information can be downloaded at https://www.mdpi.com/article/10.3390/molecules30061331/s1. Refs [20,46,47,48,49,50,53,54] are cited in Supplementary Materials.
