# Supplementary material for: Meta-Xylene-Based Diamines with Protected Benzyl Sites: Potential NCN Pincer Ligands with Tunable Steric Profiles
Source: Molecules. 2025 Mar 16;30(6):1331. doi: 10.3390/molecules30061331 (PMC11945109; doi:10.3390/molecules30061331)
Supplement: Supplementary file 1 [file molecules-30-01331-s001.zip › molecules-3504926-supplementary.pdf]

Supporting Information

Contents

Experimental 1
Representative procedure for the synthesis of the silylamines 1
Representative procedure for the synthesis of the arylamines 3
Synthesis of the dilithium salt 5Xyl-HLi_2 4
NMR reactivity studies 5
Characterization data 20
Crystallographic data 20
Mass spectra 22
NMR spectra 25
Computational details 38
References 54

Experimental

Representative procedure for the synthesis of the silylamines

The synthesis of the silylamines was performed following a modified literature procedure.[6] α^1,α^1,α^3,α^3-tetramethyl-1,3-benzenedimethanamine (3.85 g, 20.0 mmol, 1.00 eq.) was dissolved into dry THF (100 mL), and cooled using an ice bath. 2.5 M n-butyllithium in hexanes (12 mL, 30.00 mmol) was added dropwise via cannula which led to the formation of an off-white precipitate. The solution was allowed to warm up to room temperature and left to stir for 5 hours. Subsequently, the solution was cooled back down to 0°C, and trimethylchlorosilane (5.08 mL, 40.00 mmol) was added dropwise via cannula. This reaction was allowed to stir overnight. The reaction progress was monitored using NMR spectroscopy. The THF was removed under reduced pressure, and 100 mL of dry hexanes was added. After being allowed to stir for 30 minutes, the insoluble lithium chloride was filtered off, and pumping off the pentane yielded the product as a light orange oil (5.95 g, 17.7 mmol 88%). The synthetic procedure for the bis(triethylsilyl)amine and bis(triisopropylsilyl)amine was identical, except for the substitution of trimethylchlorosilane for triethylchlorosilane or triisopropylchlorosilane.

The reaction using *tert*-butyldiphenylchlorosilane turned out to be much slower than the reactions of the less bulky chlorosilanes. An additional 2-hour reflux was necessary after stirring the reaction mixture overnight to ensure completion. The product can be recrystallized from hot hexanes.

All silylamines proved to be fairly air stable and could be handled on the benchtop without noticeable decomposition.

5TMS-H_3: light orange oil, 88% yield.

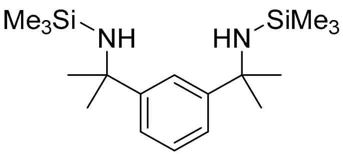

^{1}H-NMR (400 MHz, C_6D_6): δ(ppm) = 7.91 (s, 1H, C_{ipso}*H*), 7.33 (d, ^3J = 7.39 Hz, 2H, C_{meta}*H*), 7.23 (t, ^3J = 7.66 Hz, 1H, C_{para}*H*), 1.50 (s, 12H, benzyl-C*H*$_3$), 0.88 (s, 2H, N*H*), 0.10 (s, 18H, Si-C*H*$_3$). **^{13}C NMR** (100 MHz, C_6D_6): δ(ppm) = 151.6 (C_{Ar}), 127.7 (C_{Ar}), 123.4 (C_{Ar}), 123.2 (C_{Ar}), 54.9 (*C*(CH_3)$_2$), 34.0 (benzyl-*C*H_3), 2.6 (Si-*C*H_3). **ESI-HRMS:** Due to the high temperatures at the spectrometer source, only the hydrolysis product α^1,α^1,α^3,α^3-tetramethyl-1,3-benzenedimethanamine (**5H-H_3**) could be detected. The formation of **5TMS-H_3** was unambiguously confirmed via NMR spectroscopy. **Elemental Analysis**: (calcd./expt.): C (64.22/62.47), H (10.78/10.66), N (8.32/10.25). The lower-than-expected carbon content is most likely due to the formation of non-combustible silicon carbide.

5TES-H_3: light orange oil, 6.95 g (83% yield).

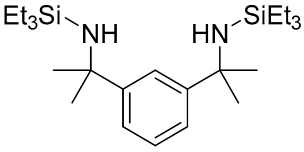

^{1}H-NMR (400 MHz, C_6D_6): δ(ppm) = 7.95 (s, 1H, C_{ipso}*H*), 7.38 (d, ^3J = 7.67 Hz, 2H, C_{meta}*H*), 7.24 (t, ^3J = 7.71 Hz, 1H, C_{para}*H*), 1.52 (s, 12H, benzyl-C*H*$_3$), 1.00 (t, ^3J = 7.94 Hz, 18H, Si-CH_2-C*H*$_3$), 0.83 (s, 2H, N*H*), 0.56 (q, ^3J = 7.92 ppm, 12H, Si-C*H*$_2$-CH_3). **^{13}C NMR** (100 MHz, C_6D_6): δ(ppm) = 152.0 (C_{Ar}), 127.8 (C_{Ar}), 123.3 (C_{Ar}), 122.7 (C_{Ar}), 54.6 (*C*(CH_3)$_2$), 33.9 (benzyl-*C*H_3), 7.7 (Si-CH_2-*C*H_3), 6.9 (Si-*C*H_2-CH_3). **APCI-HRMS (positive mode):** Calculated: 421.3429 m/z for $[C_{24}H_{49}N_2Si_2]^+$, found: 421.3433 m/z. **Elemental Analysis**: (calcd./expt.): C (68.50/67.96), H (11.50/11.63), N (6.66/7.29). The lower-than-expected carbon content is most likely due to the formation of non-combustible silicon carbide.

5TIPS-H_3: off-white powder, 3.00 g (60% yield).

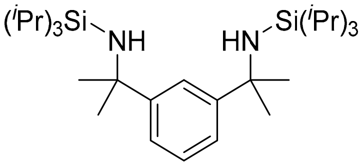

^{1}H-NMR (400 MHz, C_6D_6): δ(ppm) = 8.01 (s, 1H, C_{ipso}*H*), 7.42 (d, ^3J = 7.69 Hz, 2H, C_{meta}*H*), 7.26 (t, ^3J = 7.70 Hz, 1H, C_{para}*H*), 1.56 (s, 12H, benzyl-C*H*$_3$), 1.12-1.03 (m, 42H, Si-CH-C*H*$_3$, Si-C*H*-CH_3).

^{13}**C NMR** (100 MHz, C_6D_6): δ(ppm) = 152.5 (C_{Ar}), 128.0 (C_{Ar}), 123.1 (C_{Ar}), 122.5 (C_{Ar}), 54.5 ($C(CH_3)_2$), 33.9 (benzyl-CH_3), 19.2 (Si-CH-CH_3), 13.5 (Si-CH-CH_3). **ESI-HRMS (positive mode):** Calculated: 505.4368 m/z for $[C_{30}H_{61}N_2Si_2]^+$, found: 505.4374 m/z. **Elemental Analysis**: (calcd./expt.): C (71.35/69.48), H (11.98/11.87), N (5.55/5.44). The lower-than-expected carbon content is most likely due to the formation of non-combustible silicon carbide.

5TBP-H$_3$: off-white powder, 8.4 g (63%).

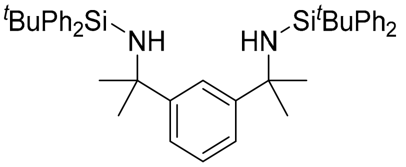

^{1}**H-NMR** (400 MHz, C_6D_6): δ(ppm) = 7.93 (s, 1H, $C_{ipso}H$), 7.91-7.88 (m, 8H, $C_{Ar}H$), 7.35 (d, ^3J = 7.77 Hz, 2H, $C_{meta}H$), 7.21-7.19 (m, 13H, $C_{Ar}H$), 1.53 (s, 2H, NH), 1.47 (s, 12H, benzyl-CH_3), 1.05 (s, 18H, C(CH_3)$_3$). ^{13}**C NMR** (100 MHz, C_6D_6): δ(ppm) = 152.3(C_{Ar}), 136.8(C_{Ar}), 136.8 (C_{Ar}) 129.4 (C_{Ar}), 128.0 (C_{Ar}), 127.7 (C_{Ar}), 123.0(C_{Ar}), 122.1(C_{Ar}), 55.3($C(CH_3)_2$), 33.5(benzyl-CH_3), 28.0 (C(CH_3)$_3$), 18.66 ($C(CH_3)_3$). **ESI-HRMS (positive mode):** Calculated: 669.4055 m/z for $[C_{44}H_{57}N_2Si_2]^+$, found: 669.4062 m/z. **Elemental Analysis**: (calcd./expt.): C (78.27/78.62), H (8.68/8.51), N (6.52/4.18). The lower-than-expected nitrogen content is most likely due to the formation of non-combustible silicon nitride.

Representative procedure for the synthesis of the arylamines

Compound **5Xyl-H$_3$** was prepared following a modified literature procedure.[7] Palladium(**II**) acetate (187 mg, 1.66 mmol, 5 mol% Pd) and (±)-2,2'-bis(diphenylphosphino)-1,1'-binaphthalene (BINAP, 1.55 g, 2.50 mmol, 0.150 eq.) were dissolved in toluene (10 mL) and heated to 100 °C for 15 min in a Schlenk bomb. Sodium *tert*-butoxide (4.80 g, 49.9 mmol, 3.00 eq.), α^1,α^1,α^3,α^3-tetramethyl-1,3-benzenedimethanamine (3.20 g, 16.6 mmol, 1.00 eq.), 2-bromo-*m*-xylene (7.39 g, 39.9 mmol, 2.40 eq.) and toluene (100 mL) were added and the reaction suspension was heated at 150 °C for 90 h. The reaction progress was monitored via thin layer chromatography. While the reaction was performed under inert gas conditions, the work-up was performed under air using solvents that weren't dried prior to usage. Upon reaction completion, the crude reaction was filtered through celite and washed with dichloromethane until the celite became colourless. The solvent was removed under reduced pressure and the residue was purified by column chromatography (100% hexanes, then 10% EtOAc/hexanes). After removal of the solvents under vacuum, the product was obtained as a colourless to light orange oil (6.27 g, 15.65 mmol, 94% yield). Crystals suitable for X-ray diffraction analysis were obtained by adding concentrated hydrochloric acid to a solution of **5Xyl-H$_3$** in ethanol and storing the solution in the freezer at –18 °C for 2 weeks.

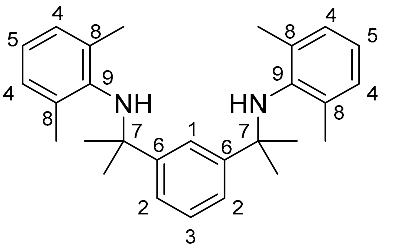

^{1}H-NMR (500 MHz,$CDCl_3$): δ(ppm) = 7.87 (t, ^{4}J =1.86 Hz, 1H, *H1*), 7.46 (dd, ^{3}J = 7.73 Hz, ^{4}J =1.94 Hz, 2H, *H2*), 7.29 (t, ^{3}J =7.75 Hz, 1H, *H3*), 6.95 (d, ^{3}J = 7.59 Hz, 4H, *H4*), 6.84 (t, ^{3}J =7.46 Hz, 2H, *H5*), 3.23 (br s, 2H, N*H*), 2.04 (s, 12H, Xyl-C*H*$_3$), 1.52 (s, 12H, NH-C*H*$_3$). **^{13}C NMR** (125.75 MHz, $CDCl_3$): δ(ppm) = 149.97 (C_{Ar}), 144.54 (C_{Ar}), 133.38 (C_{Ar}), 128.61 (C_{Ar}), 127.48 (C_{Ar}), 123.34 (C_{Ar}), 123.23 (C_{Ar}), 122.64 (C_{Ar}), 58.29 (NH-*C*$(CH_3)_2$), 31.13 (NH-C(*C*$H_3)_2$), 20.50 (Xyl-*C*H_3). **ESI-HRMS (positive mode):** Calculated: 423.2775 m/z for $[C_{28}H_{36}N_2Na]^+$, found: 423.2771 m/z. **Elemental Analysis**: (calcd./expt.): C (83.95/83.79), H (9.06/9.09), N (6.99/7.27).

For the synthesis of **5Trip-H$_3$**, the catalyst loading was increased to 10 mol% Pd while keeping all other conditions identical. **5Trip-H$_3$** was isolated as an orange oil (<5% yield).

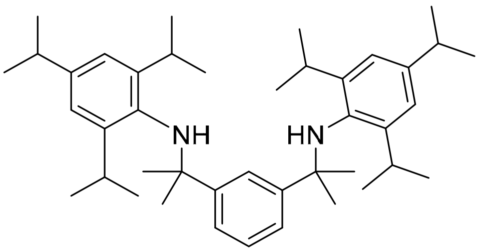

ESI-HRMS (positive mode): Calculated: 597.5147 m/z for $[C_{42}H_{65}N_2]^+$, found: 597.5147 m/z.

Synthesis of the dilithium salt 5Xyl-HLi$_2$

The reaction was performed under inert gas atmosphere as all dilithium salts are highly moisture sensitive. **5Xyl-H$_3$** (1.00 g, 2.50 mmol, 1eq.) was weighed into a Schlenk flask and dissolved in hexanes (10 mL). The reaction solution was cooled down to –30 °C and 1.6 M n-butyllithium in hexanes (3.9 mL, 6.24 mmol, 2.5 eq.) was added dropwise using a syringe while stirring which led to the formation of a pale-yellow precipitate. The reaction was stirred overnight while allowing to warm up to room temperature. After removing all volatile parts under vacuum, **5Xyl-HLi$_2$** was isolated as a yellow powder (1.00 g, 2.43 mmol, 97% yield). In most cases, no further purification was needed. Optionally, the product could be further purified by washing with hexanes (yield: 56%).

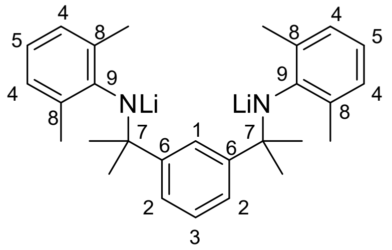

^{1}H-NMR (500 MHz, thf-d_8): δ(ppm) = 7.80 (s, 1H, *H1*), 7.31 (dd, ^3J = 7.60 Hz, ^4J = 1.78 Hz, 2H, *H2*), 6.94 (t, ^3J = 7.60 Hz, 1H, *H3*), 6.70 (d, ^3J = 7.20 Hz, 4H, *H4*), 6.15 (t, ^3J = 7.23 Hz, 2H, *H5*), 2.02 (s, 12H, Xyl-C*H*$_3$), 1.39 (s, 12H, NH-C*H*$_3$). **^{13}C NMR** (125.75 MHz, thf-d_8): δ(ppm) = 162.50 (C_{Ar}), 157.16(C_{Ar}), 135.99(C_{Ar}), 128.35(C_{Ar}), 126.27(C_{Ar}), 123.46(C_{Ar}), 122.14(C_{Ar}), 114.28(C_{Ar}), 61.53 (NLi-*C*(CH$_3$)$_2$), 35.20 (NLi-C(*C*H$_3$)$_2$), 23.20 (Xyl-*C*H$_3$).

NMR reactivity studies

Remark:

When using non-deuterated solvents, a solvent suppression algorithm was used. Spectra measured in non-deuterated solvents were either calibrated to remaining solvent signals or to other solvents that were present in trace amounts in the sample. Due to the suppression algorithm applied not all spectra could be phased correctly. Where a phase correction was not possible, the magnitude spectrum is displayed.

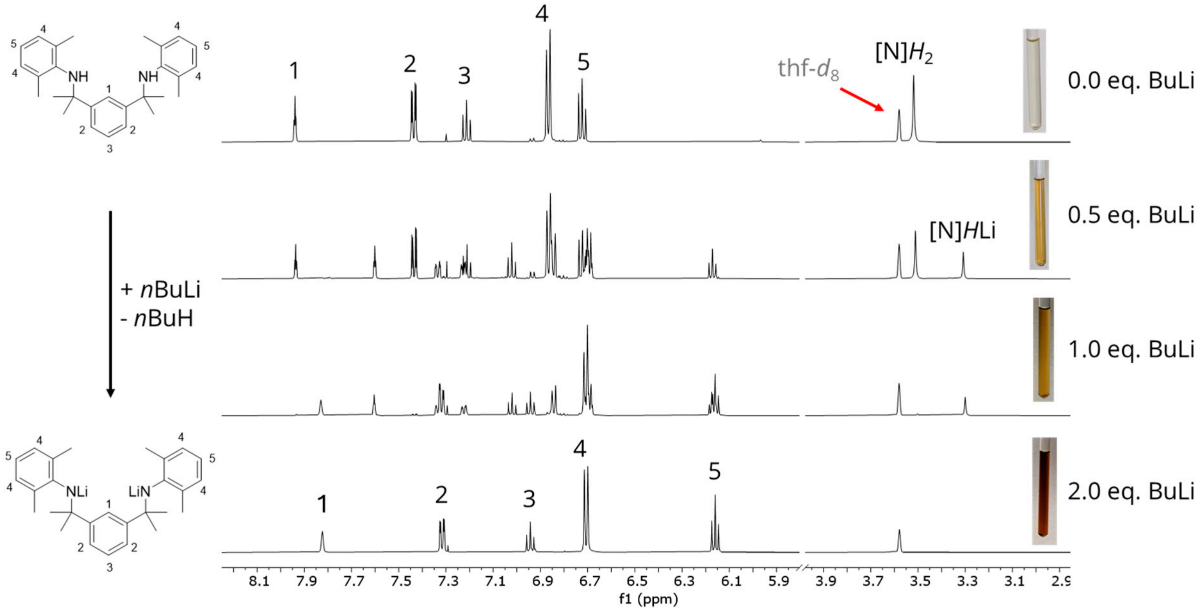

Figure S1. Stacked ^{1}H NMR spectra monitoring the stepwise addition of a 1.6 M *n*BuLi solution to a solution of **5Xyl-H$_3$** in thf-d_8 to form **5Xyl-HLi$_2$**.

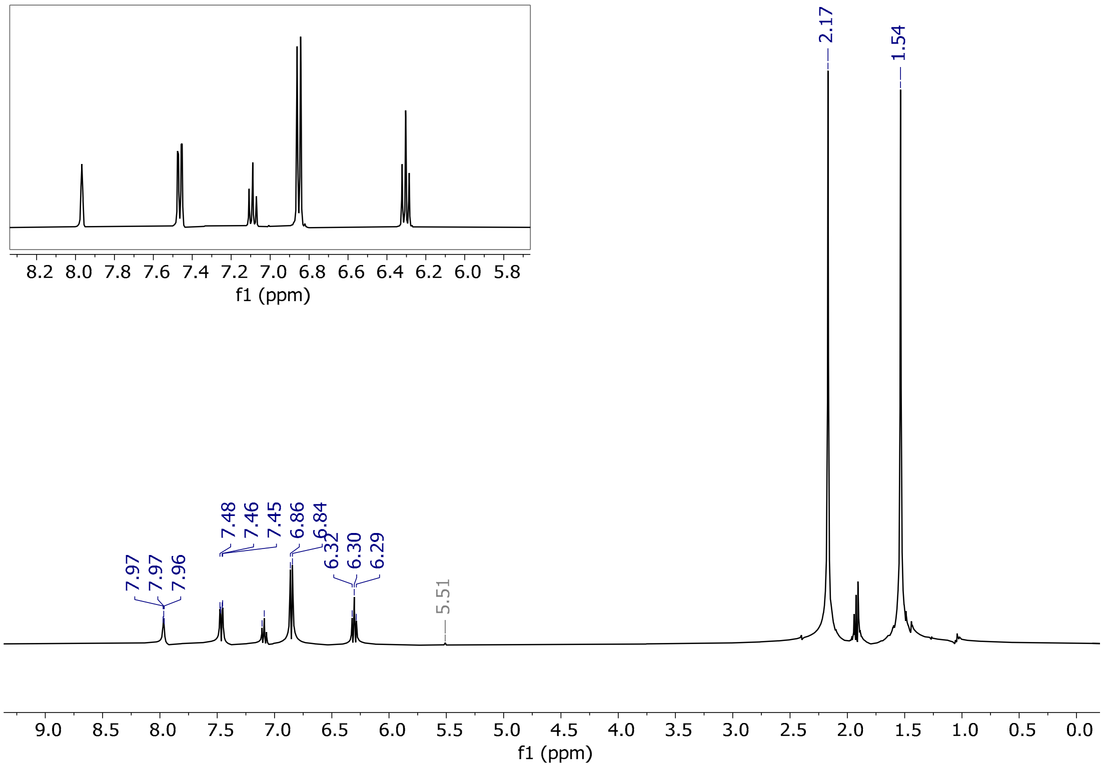

Figure S2. ^{1}H NMR spectrum of **5Xyl-HLi$_2$** in thf-h_8 calibrated to residual dichloromethane. Magnitude spectrum displayed.

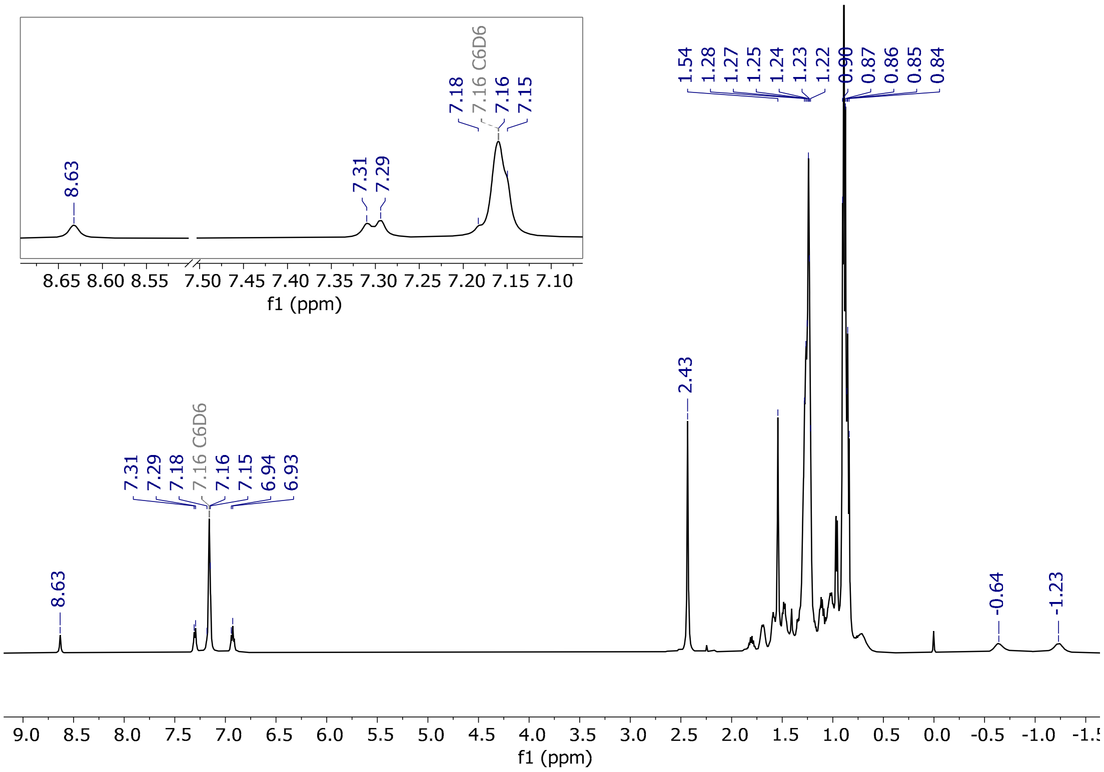

Figure S3. ^{1}H NMR spectrum of **5Xyl-HLi$_2$** in C_6D_6.

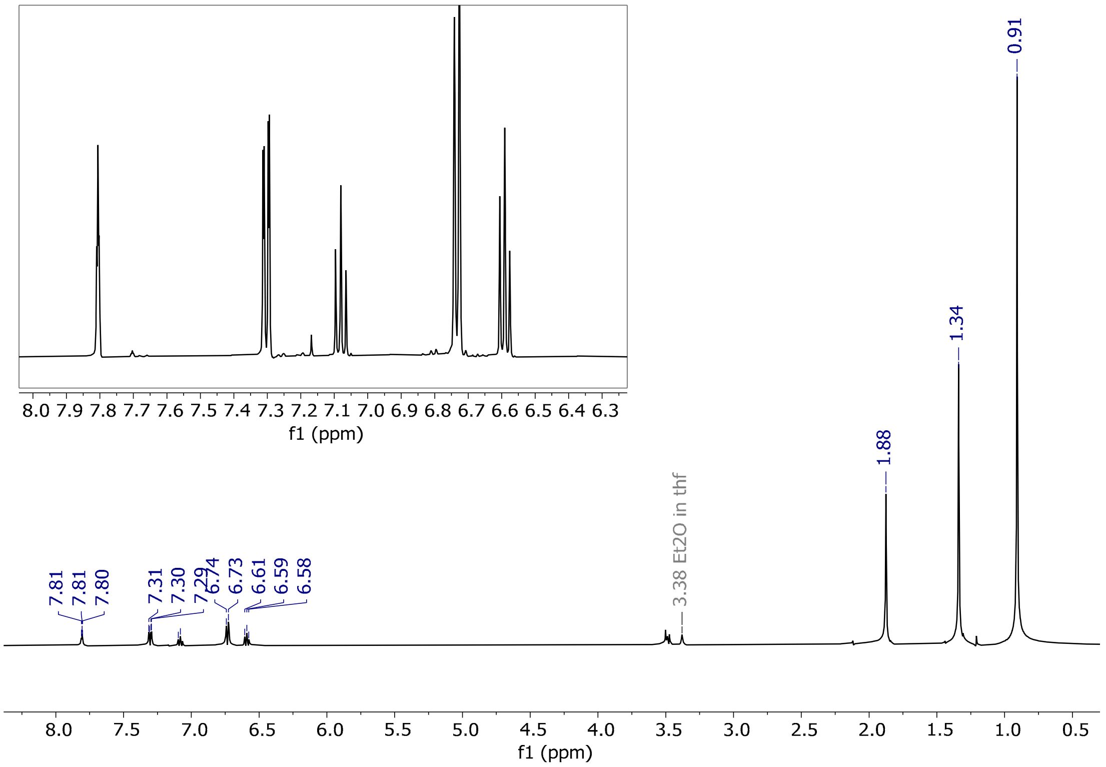

Figure S4. ^{1}H NMR spectrum of a solution of **5Xyl-H$_3$** in thf-*h*$_8$ calibrated to residual Et_2O after adding 2 eq. of NaO*t*Bu. The NMR spectrum appears unchanged. Magnitude spectrum displayed.

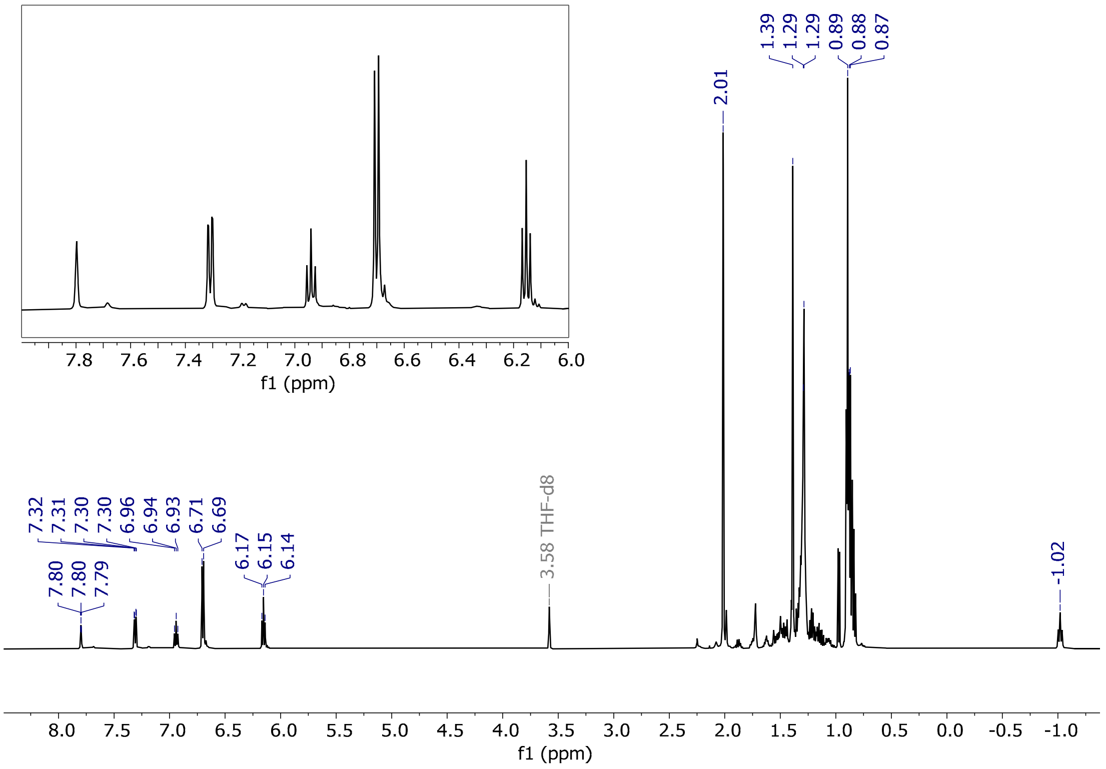

Figure S5. ^{1}H NMR spectrum of a solution of **5Xyl-HLi$_2$** in thf-*d*$_8$ after adding 1.2 eq of *n*BuLi (in hexanes). The characteristic triplet at -1.02 ppm was assigned to unreacted *n*BuLi in accordance with literature reports.[8]

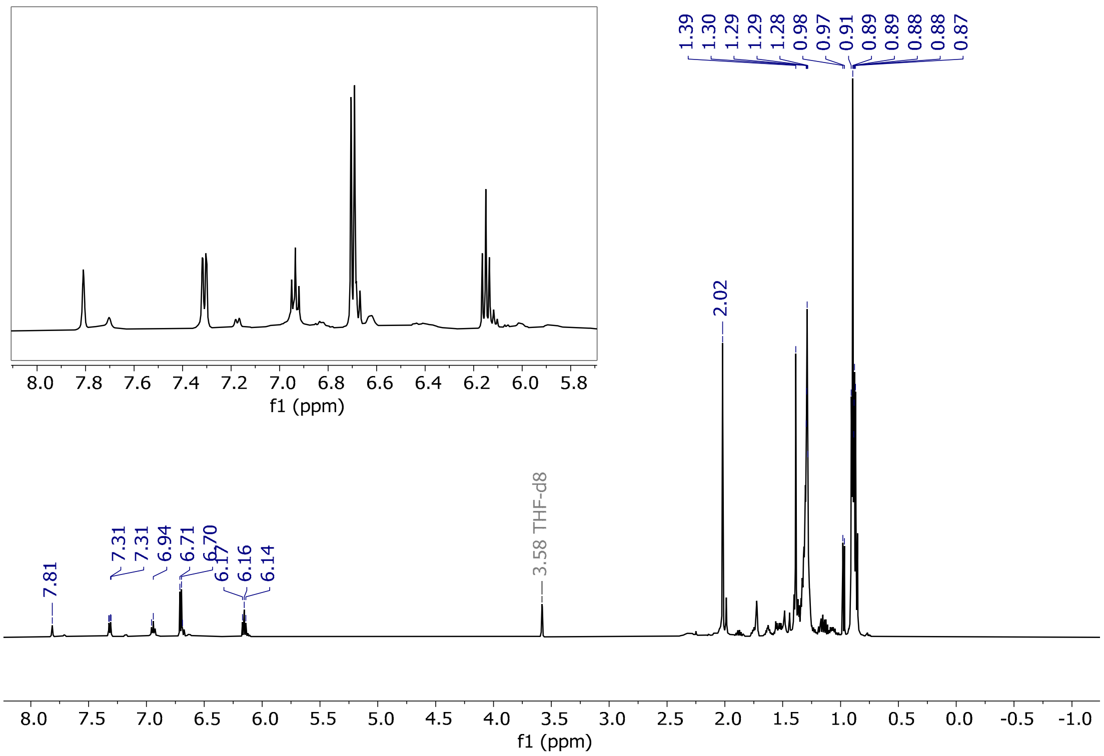

Figure S6. ^{1}H NMR spectrum of a solution of **5Xyl-HLi$_2$** in thf-d_8 after adding 1.2 eq of *n*BuLi (in hexanes) and heating to 65 °C for 30 min. The resonance peaks corresponding to **5Xyl-HLi$_2$** appear unchanged.

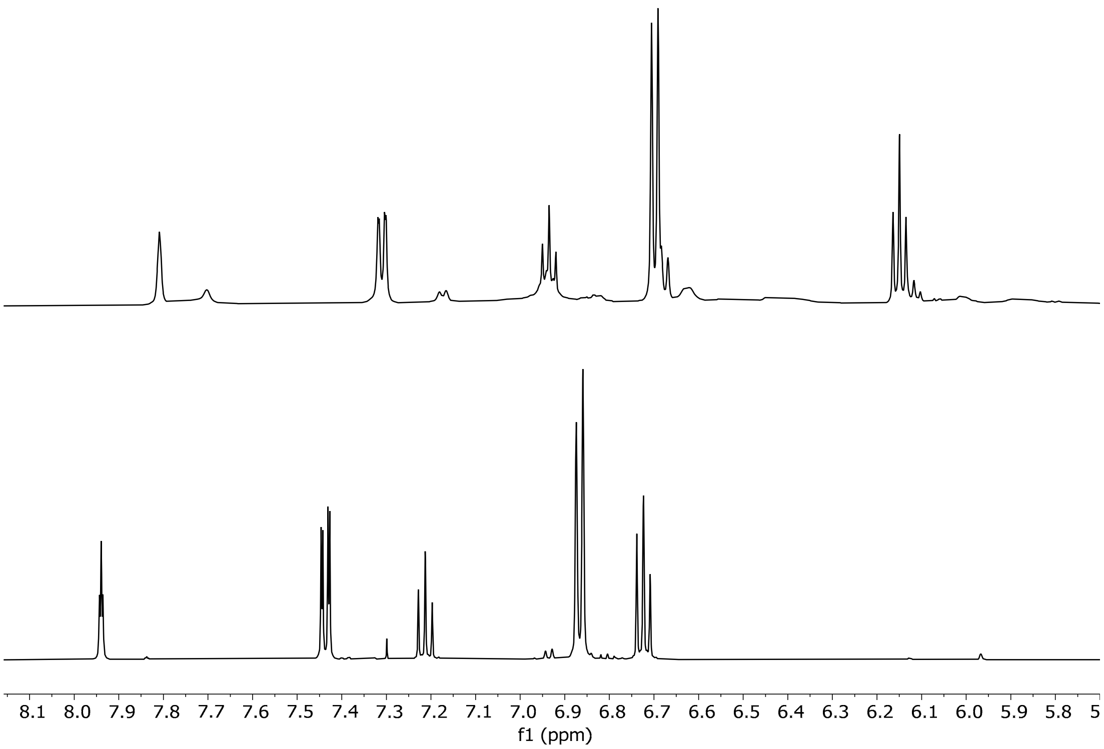

Figure S7. Stacked ^{1}H NMR spectra in thf-d_8 of a solution of **5Xyl-HLi$_2$** (bottom) and a solution of **5Xyl-HLi$_2$** after adding 1.2 eq of *n*BuLi (in hexanes) and heating to 65 °C for 30 min (top). The minor signals observed in the top spectrum do not align with the signals of **5Xyl-H$_3$** in them bottom spectrum.

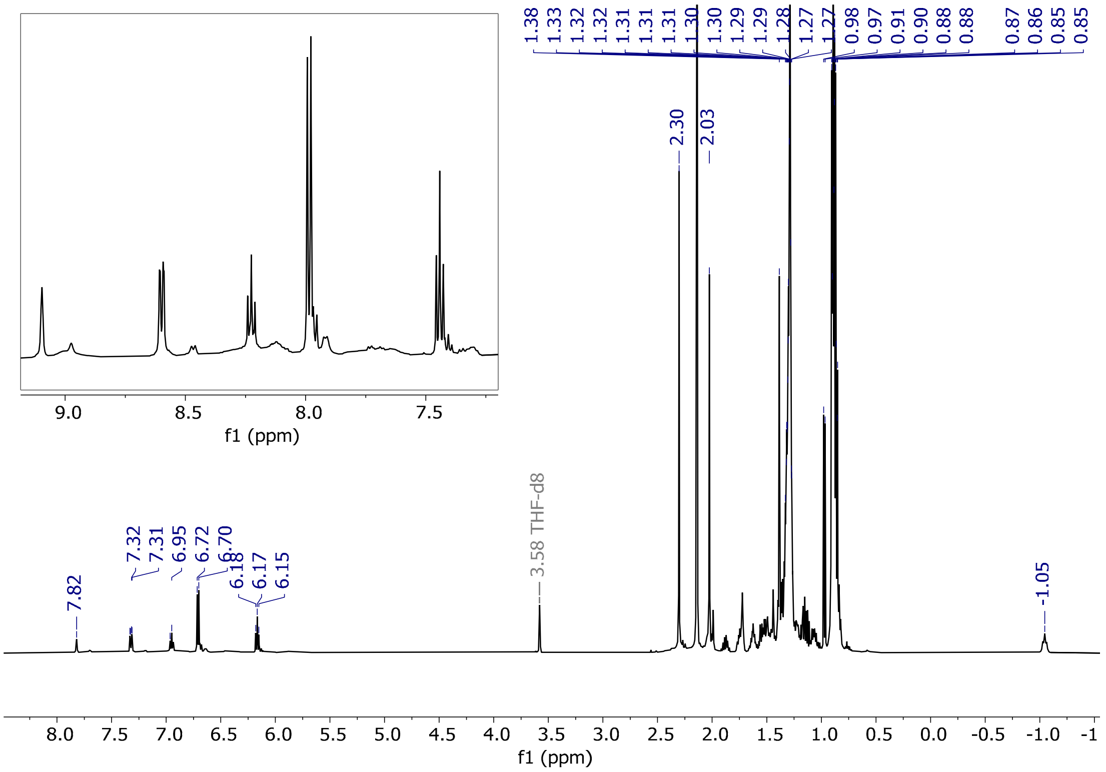

Figure S8. ^{1}H NMR spectrum of a solution of **5Xyl-HLi$_2$** in thf-d_8 after adding 1.2 eq of *n*BuLi (in hexanes) and 1.2 eq. of TMEDA. The resonance peaks corresponding to **5Xyl-HLi$_2$** appear unchanged. The peak at –1.05 ppm corresponds to unreacted *n*BuLi.

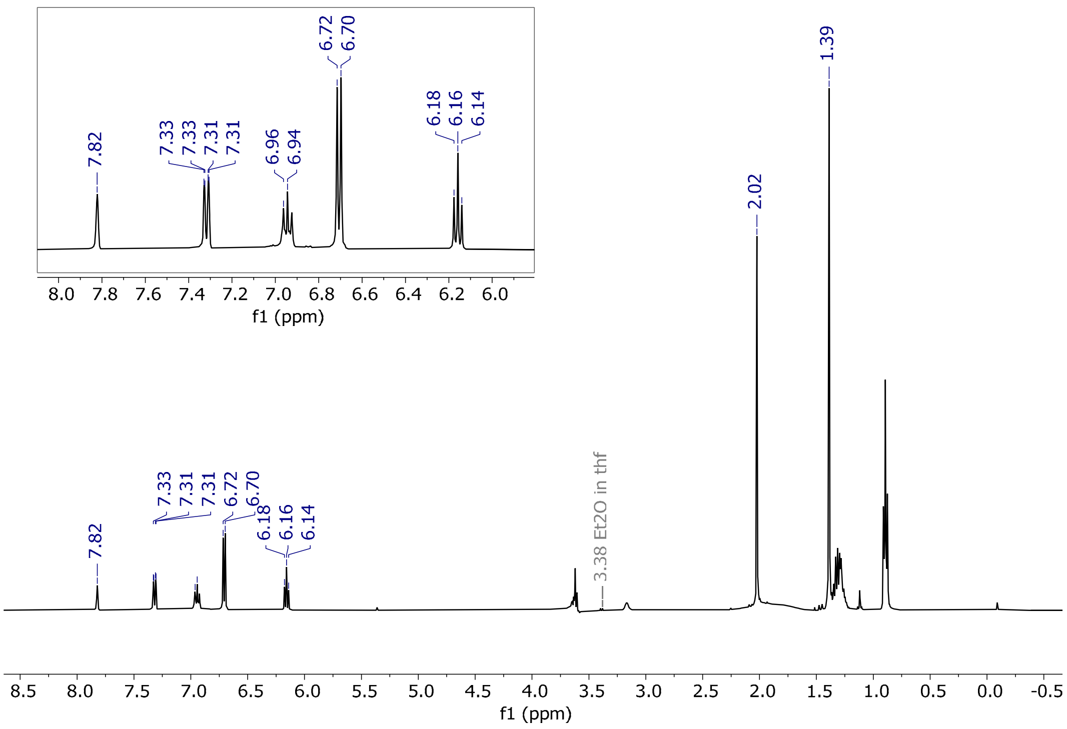

Figure S9. ^{1}H NMR spectrum of a solution of **5Xyl-HLi$_2$** in thf-h_8 after adding *t*BuLi.

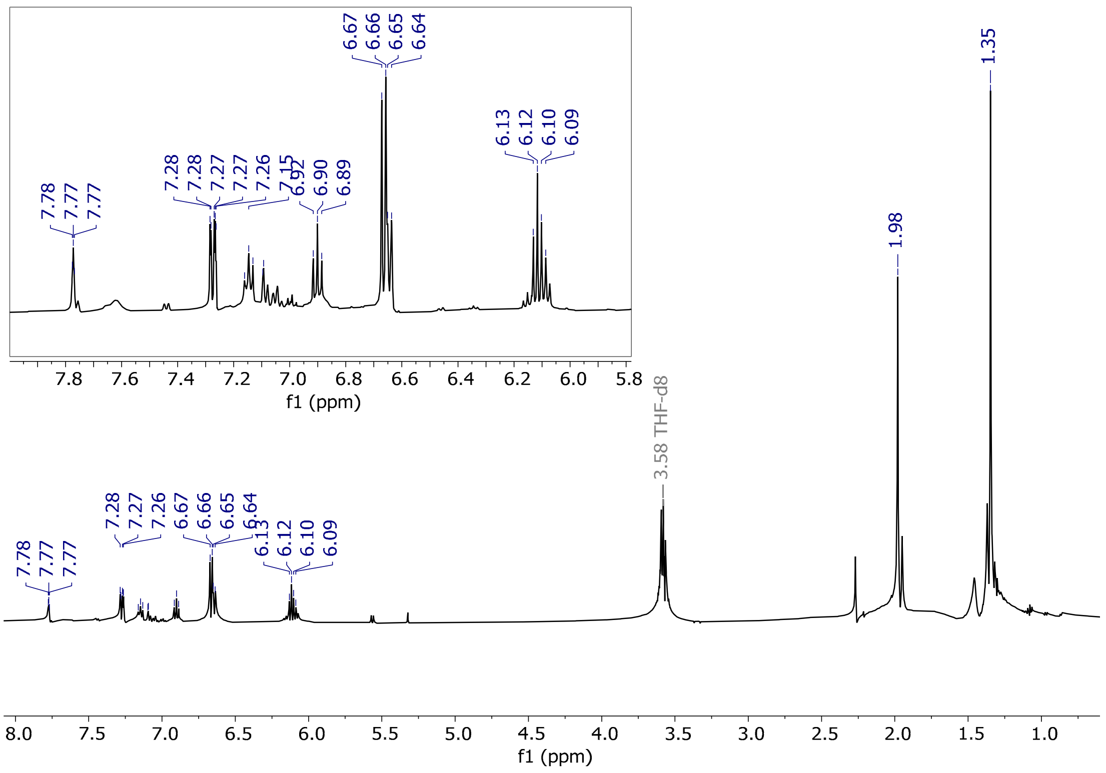

Figure S10. ^{1}H NMR spectrum of a solution of **5Xyl-HLi$_2$** in thf-h_8 calibrated to residual solvent signal after refluxing **5Xyl-HLi$_2$** with 1.2 eq. *n*BuLi in toluene for 30 min. The resonance peaks corresponding to **5Xyl-HLi$_2$** appear unchanged. Magnitude spectrum displayed.

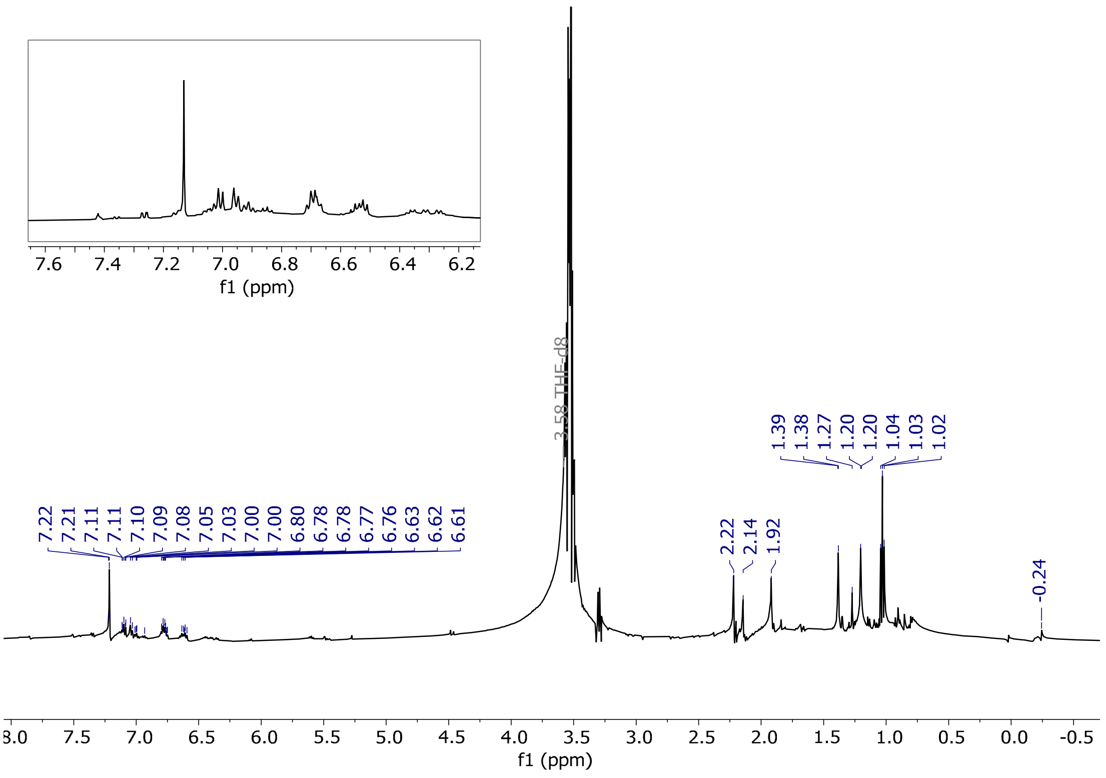

Figure S11. ^{1}H NMR spectrum of a solution of **5Xyl-HLi$_2$** in thf-h_8 calibrated to residual solvent signal after refluxing **5Xyl-HLi$_2$** with 1.2 eq. MeLi in toluene for 1 h. Magnitude spectrum displayed.

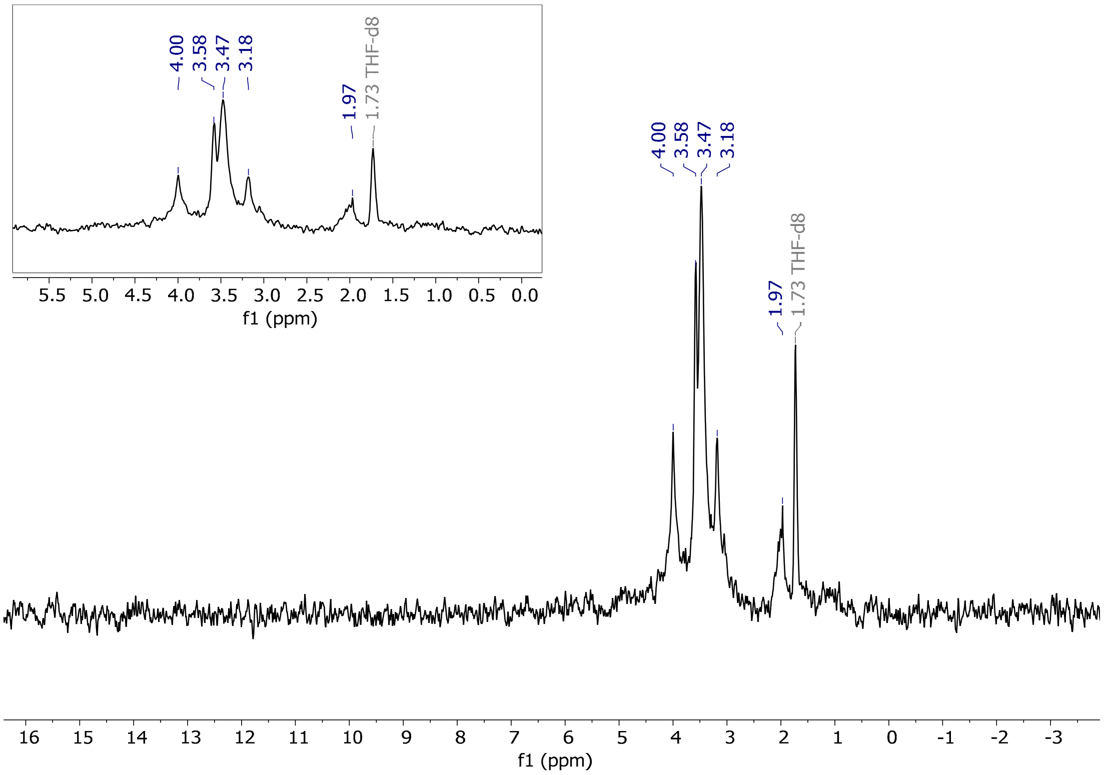

Figure S12. ^{2}H NMR spectrum in thf-h_8 after quenching a reaction mixture of **5Xyl-HLi$_2$** and MeLi with MeOD-d_4.

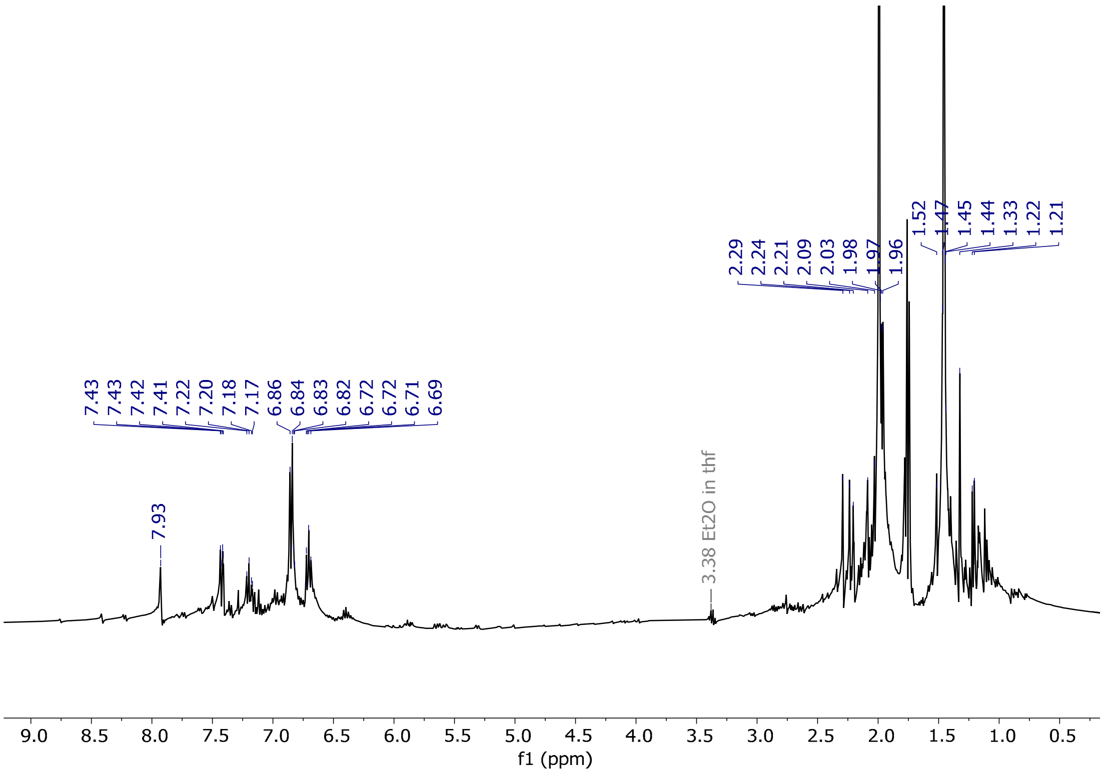

Figure S13. ^{1}H NMR spectrum in thf-h_8 calibrated to residual Et_2O after quenching a reaction mixture of **5Xyl-HLi$_2$** and MeLi with MeOD-d_4. Magnitude spectrum displayed.

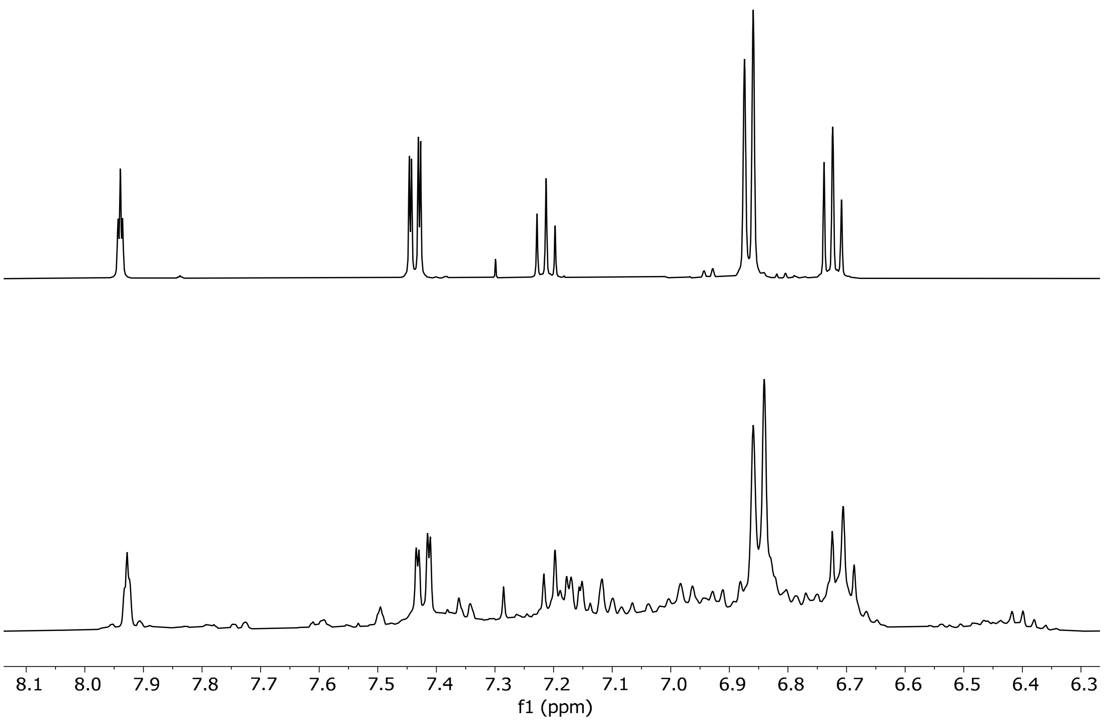

Figure S14. ^{1}H NMR spectra, stacked in thf. Top: **5Xyl-H$_3$** in thf-d_8. Bottom: A reaction mixture of **5Xyl-HLi$_2$** and MeLi after quenching with MeOD-d_4.

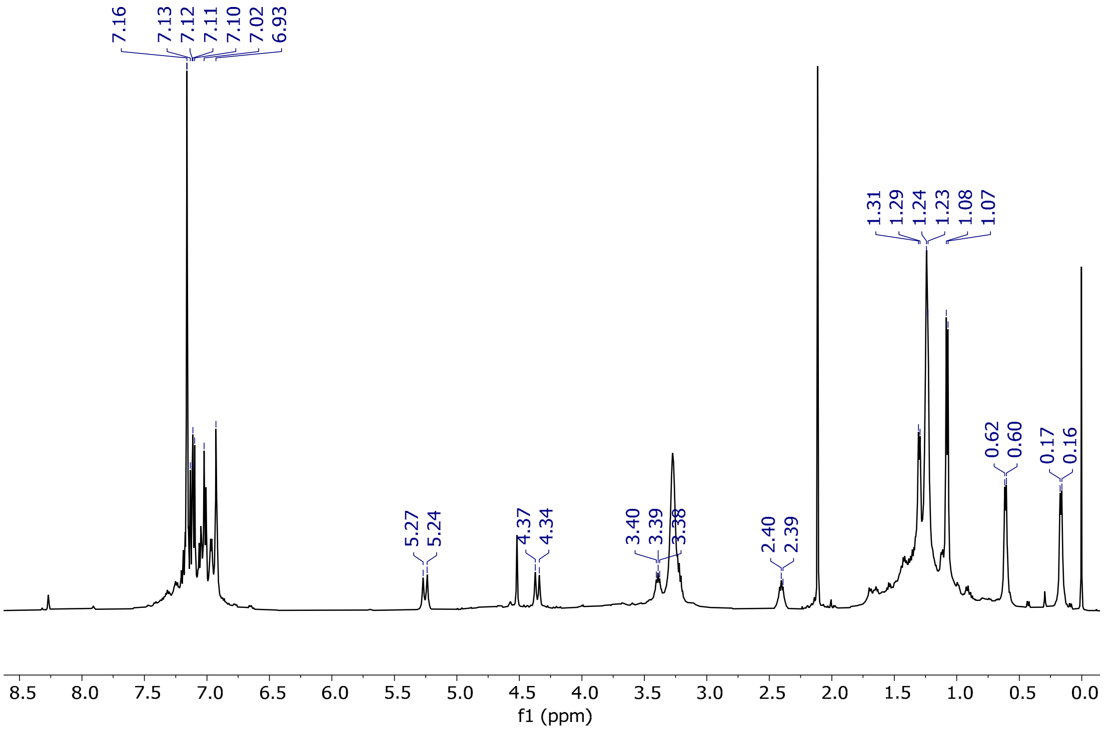

Figure S15. ^{1}H NMR spectrum of **3Dipp-Li$_3$** in C_6D_6. The spectrum contains minor impurities such as toluene (2.11 ppm) or diethyl ether (3.27 ppm) as the synthesis of **3Dipp-Li$_3$** purely served as a proof-of-concept and no further purification was attempted.

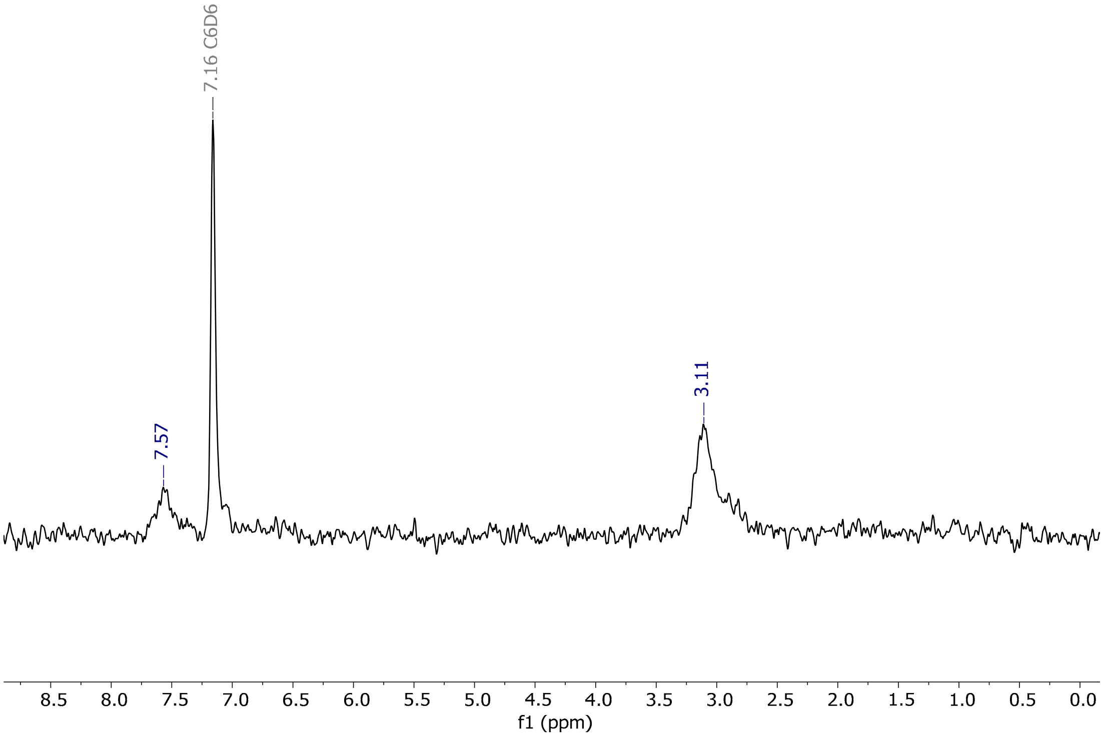

Figure S16. ^{2}H NMR spectrum in thf-h_8 after quenching a reaction mixture of **3Dipp-Li$_3$** and MeLi with MeOD-d_4.

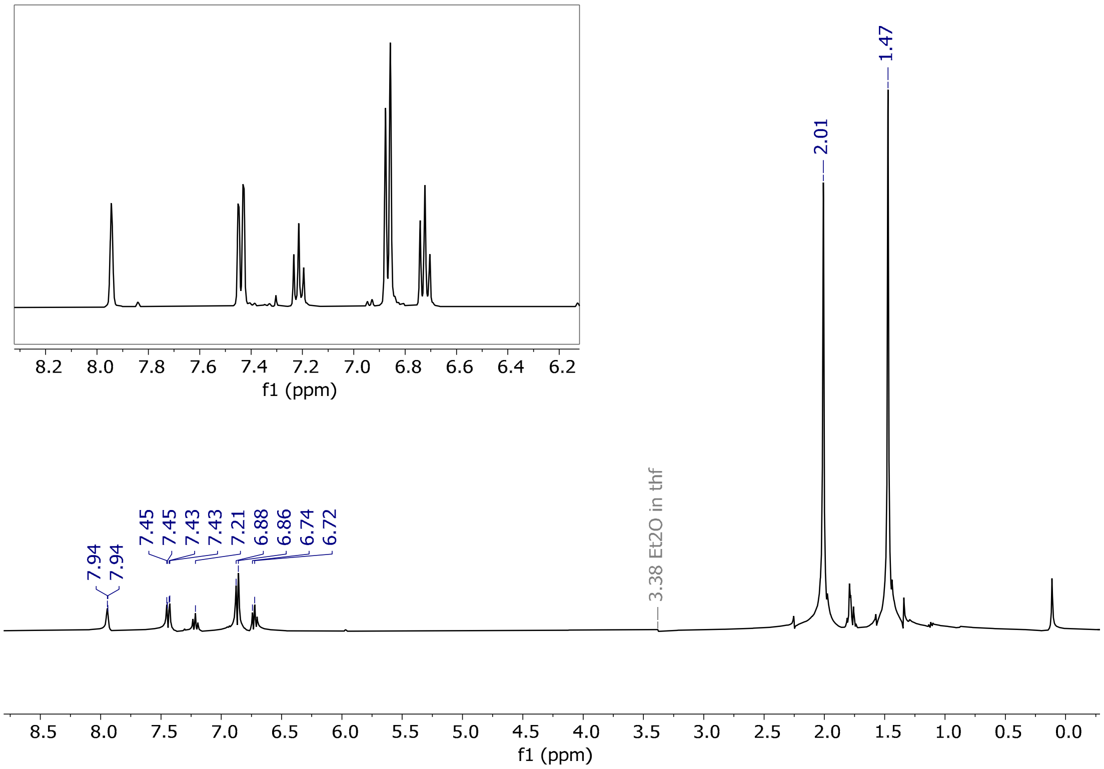

Figure S17. ^{1}H NMR spectrum of a solution of **5Xyl-HLi$_2$** in thf-h_8 calibrated to residual Et_2O after reaction with 10 eq. *sec*BuLi. Magnitude spectrum displayed.

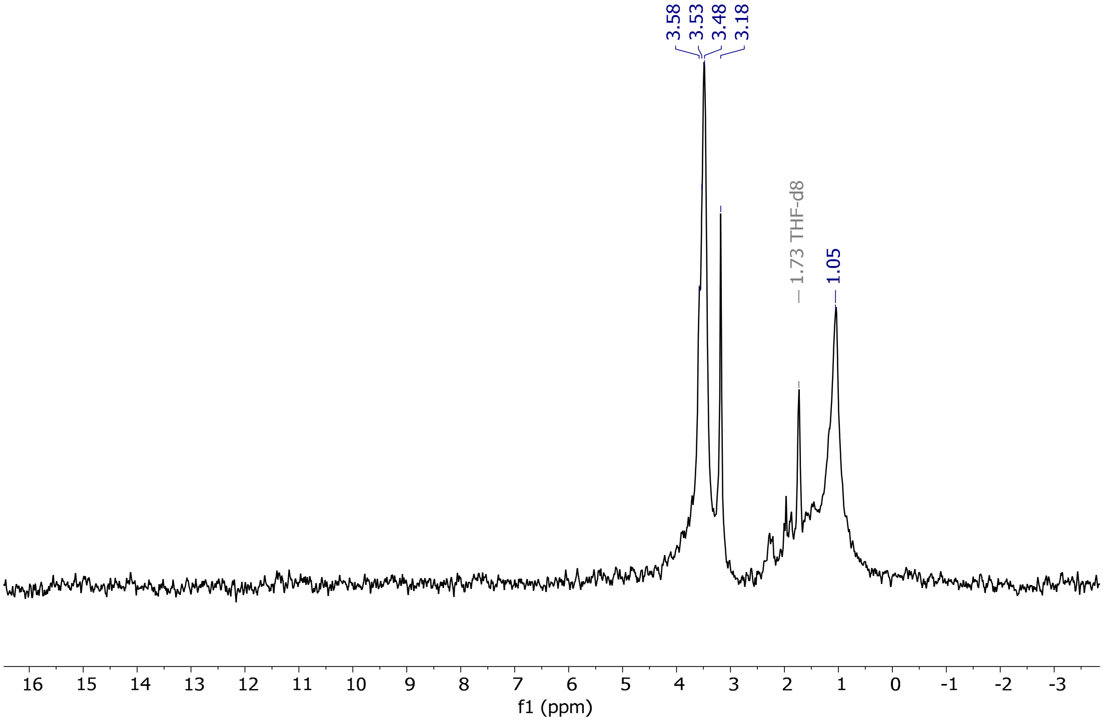

Figure S18. ^{2}H NMR spectrum in thf-h_8 after quenching a reaction mixture of **5Xyl-HLi$_2$** and *sec*BuLi with MeOD-d_4.

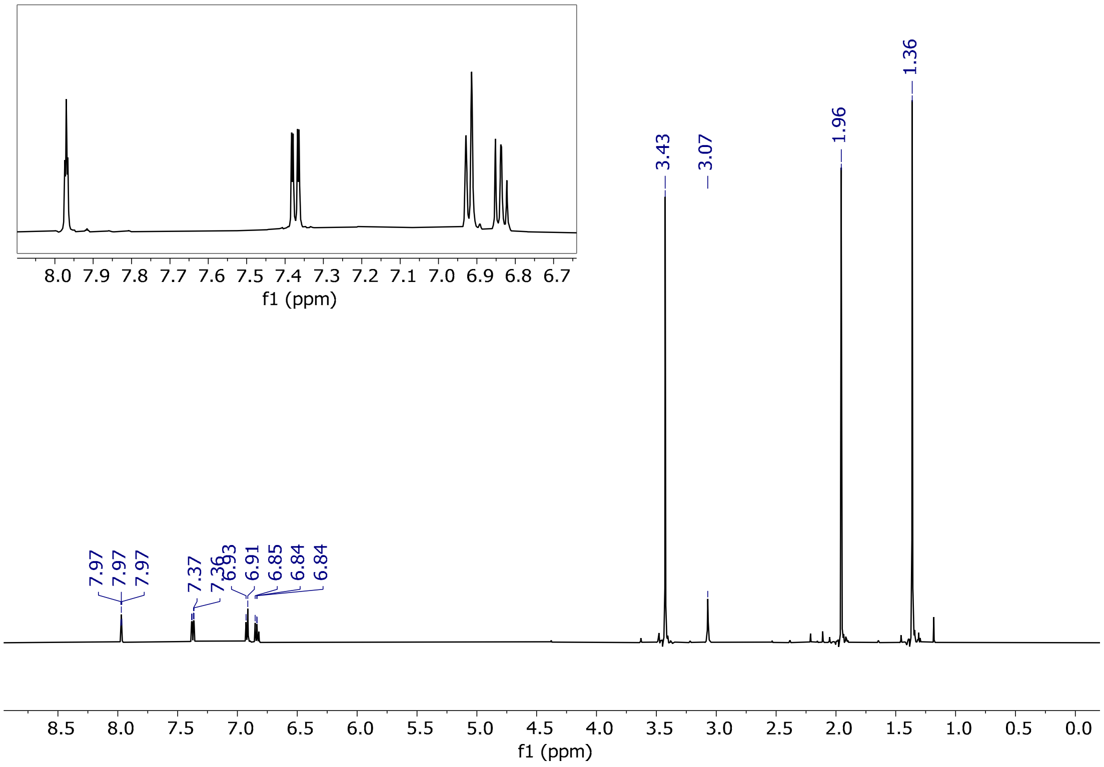

Figure S19. ^{1}H NMR spectrum of a reaction solution of **5Xyl-H$_3$,** Bi(NMe$_2$)$_3$ and pyridine (catalytic amount) after stirring at room temperature for 2 days. Redissolved in in C_6H_6 after solvent removal and calibrated to residual toluene in benzene.

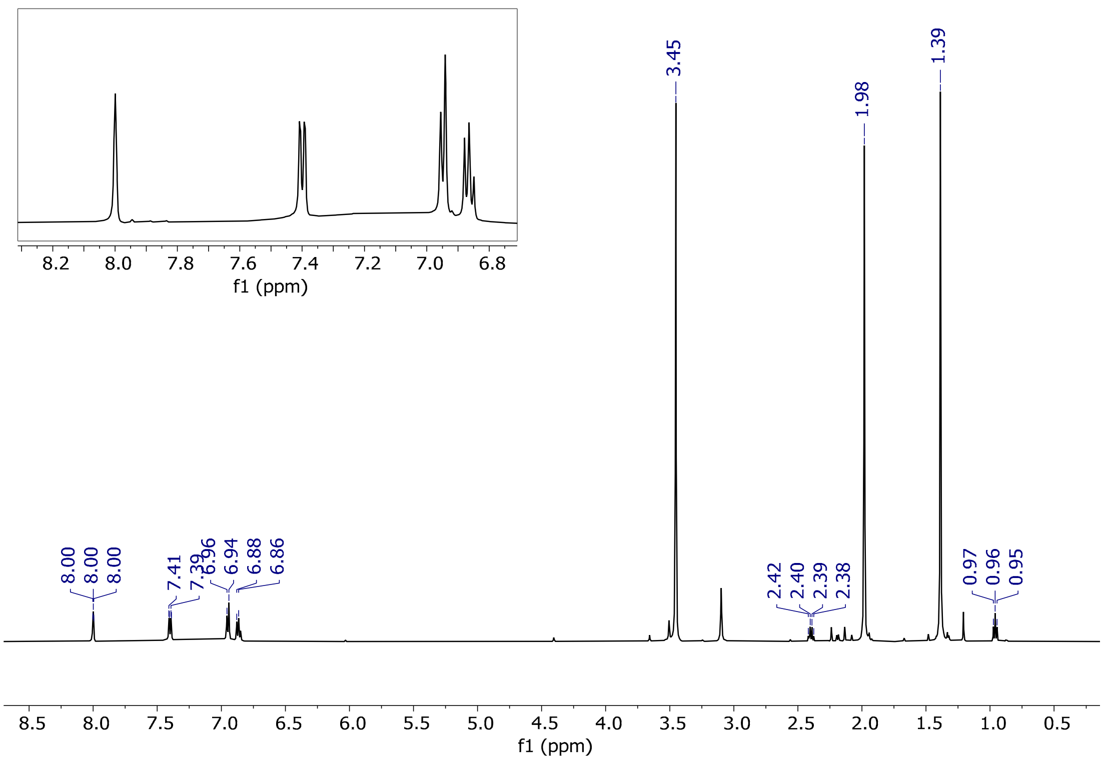

Figure S20. ^{1}H NMR spectrum of a reaction solution of **5Xyl-H₃**, Bi(NMe$_2$)$_3$ and triethylamine (catalytic amount) in C_6H_6 after heating to 110 °C for 90 min. Calibrated to triethylamine in benzene.

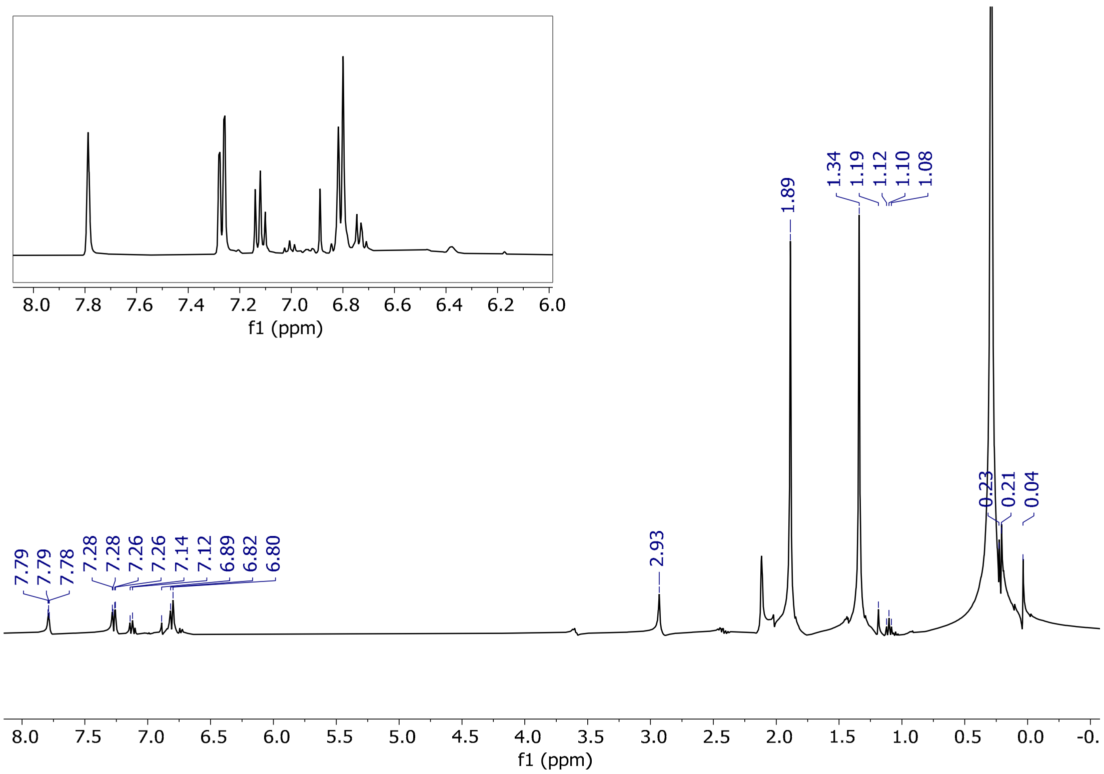

Figure S21. ^{1}H NMR spectrum of a reaction solution of **5Xyl-H$_3$**, Bi(HMDS)$_3$ and pyridine (catalytic amount) in proteo-mesitylene after heating to 150 °C for 3 d. Magnitude spectrum displayed.

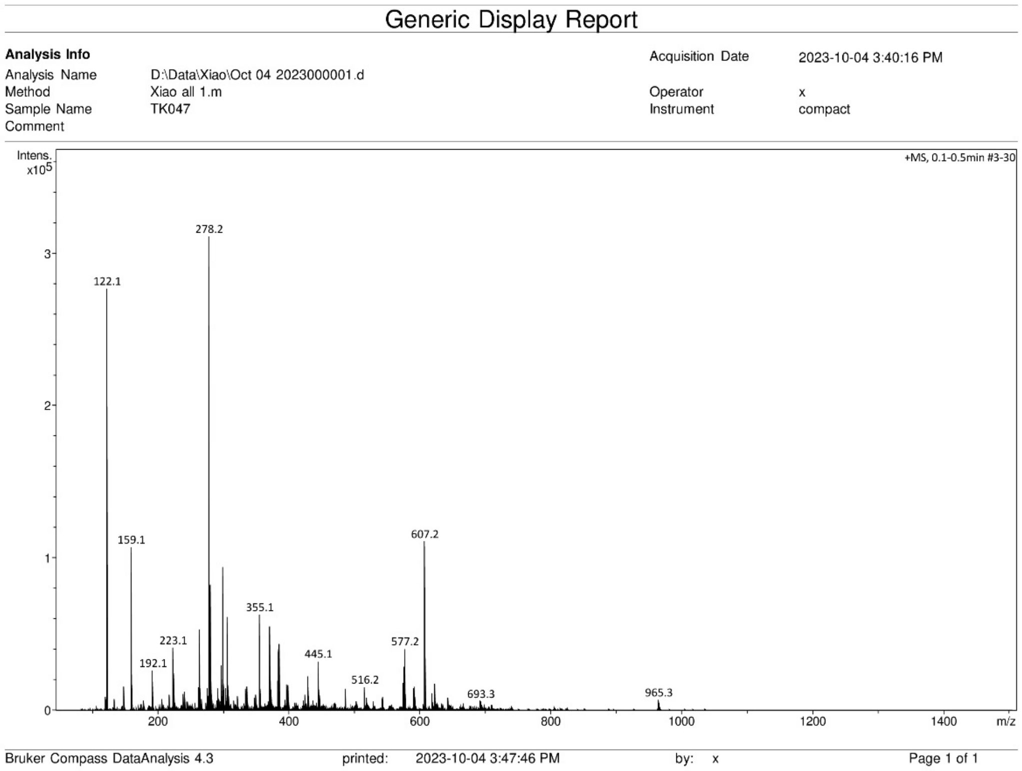

Figure S22. ESI(+) mass spectrum of a reaction mixture of **5Xyl-HLi$_2$** and $BiCl_3$ in the range from 50 to 1500 m/z. **5Xyl-HBi$^+$**can be found at 607.2 m/z.

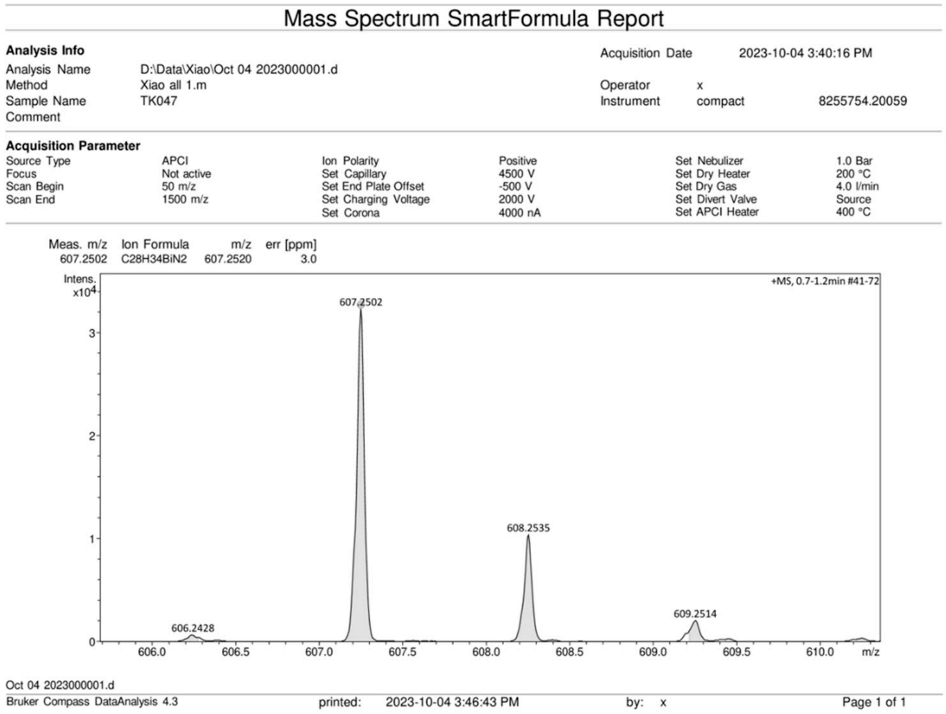

Figure S23. Calibrated high-resolution ESI(+) mass spectrum of **5Xyl-HBi$^+$**.

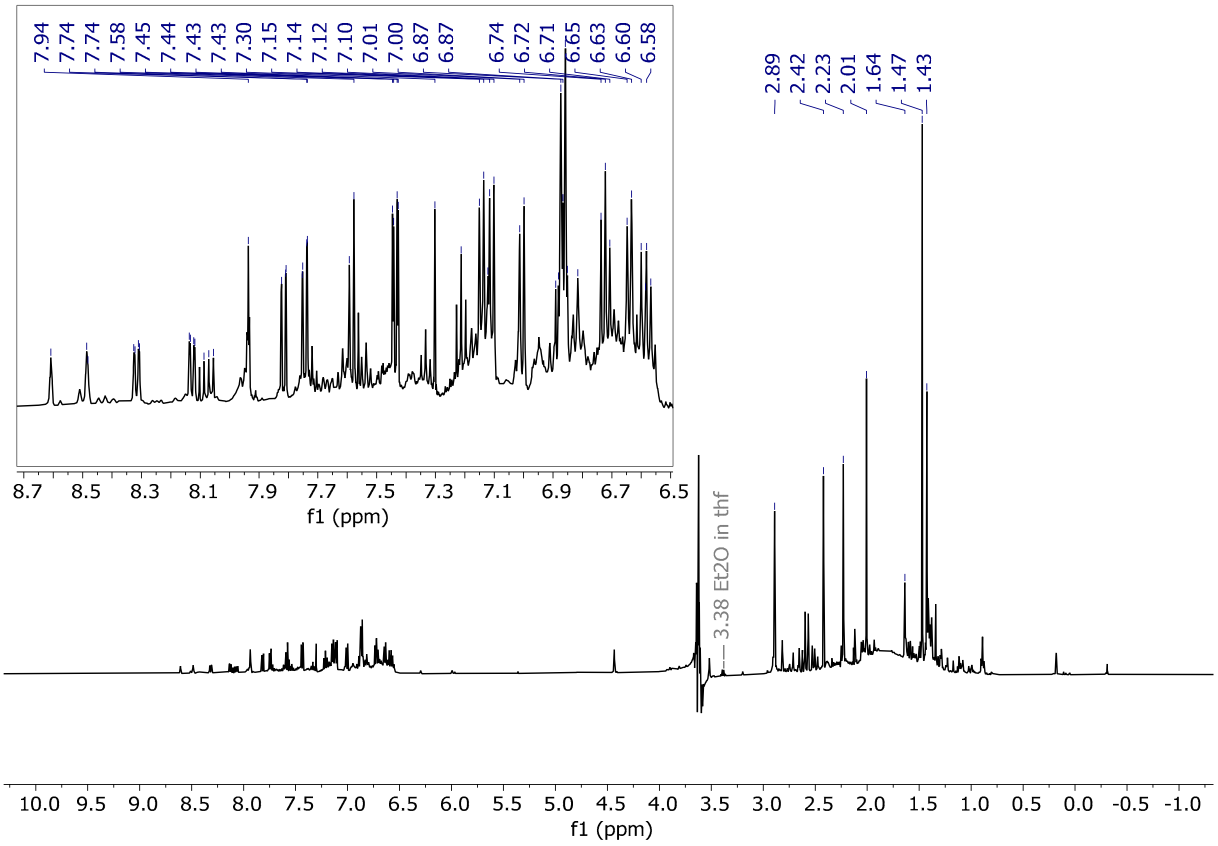

Figure S24. ^{1}H NMR spectrum of a reaction solution of **5Xyl-HLi$_2$** and $BiCl_3$ in thf-h_8. Calibrated to residual diethyl ether in thf.

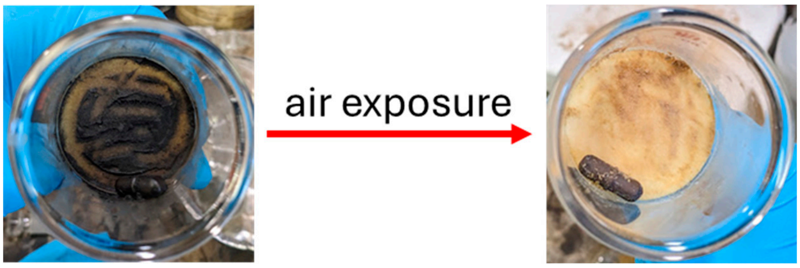

Figure S25. Dark precipitate formed during the synthesis and the workup of **5Xyl-HBiCl** before and after air exposure.

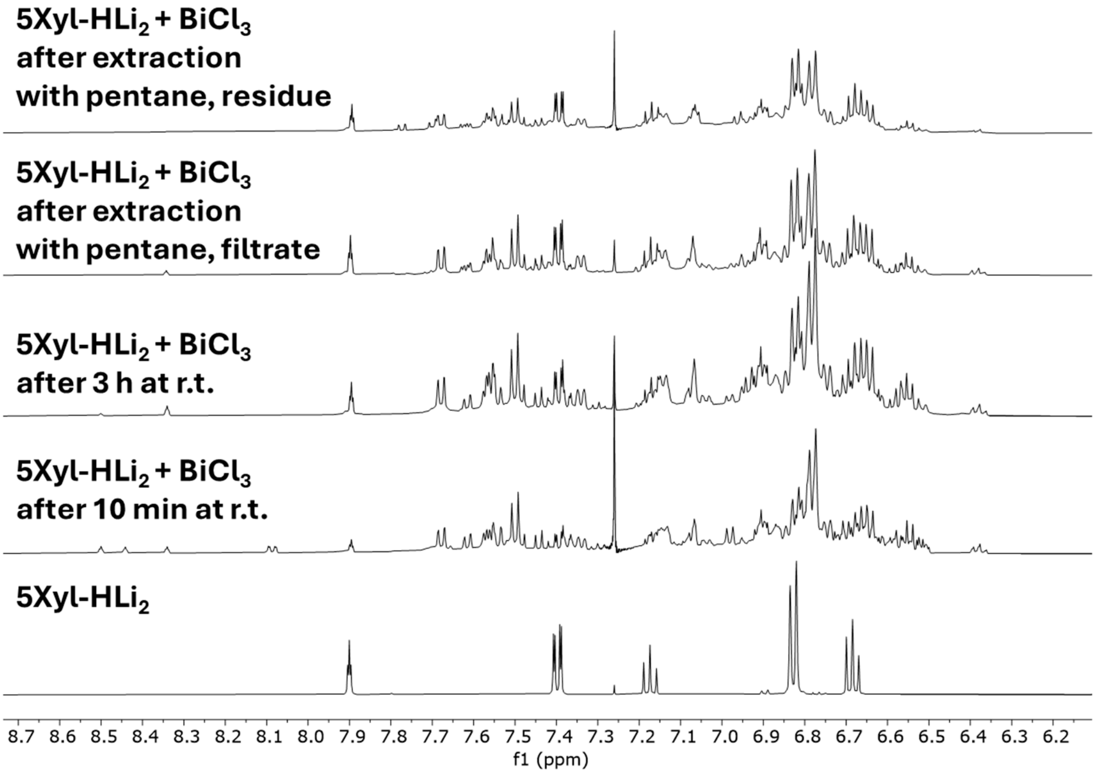

Figure S26. Stacked ^{1}H NMR spectra of the reaction of **5Xyl-HLi₂** and $BiCl_3$ in thf-h_8. The relative intensity of the protonated ligand **5Xyl-H₃** (cf. bottom spectrum) increases over time.

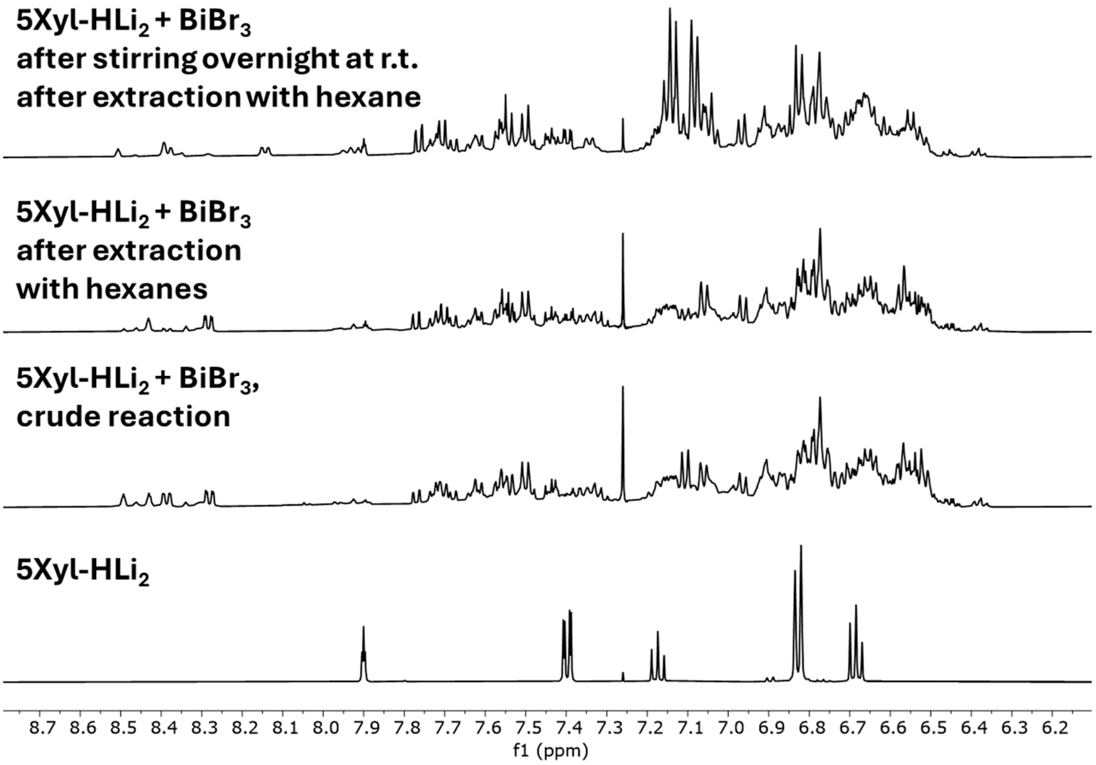

Figure S27. Stacked ^{1}H NMR spectra of the reaction of **5Xyl-HLi₂** and $BiBr_3$ in thf-h_8.

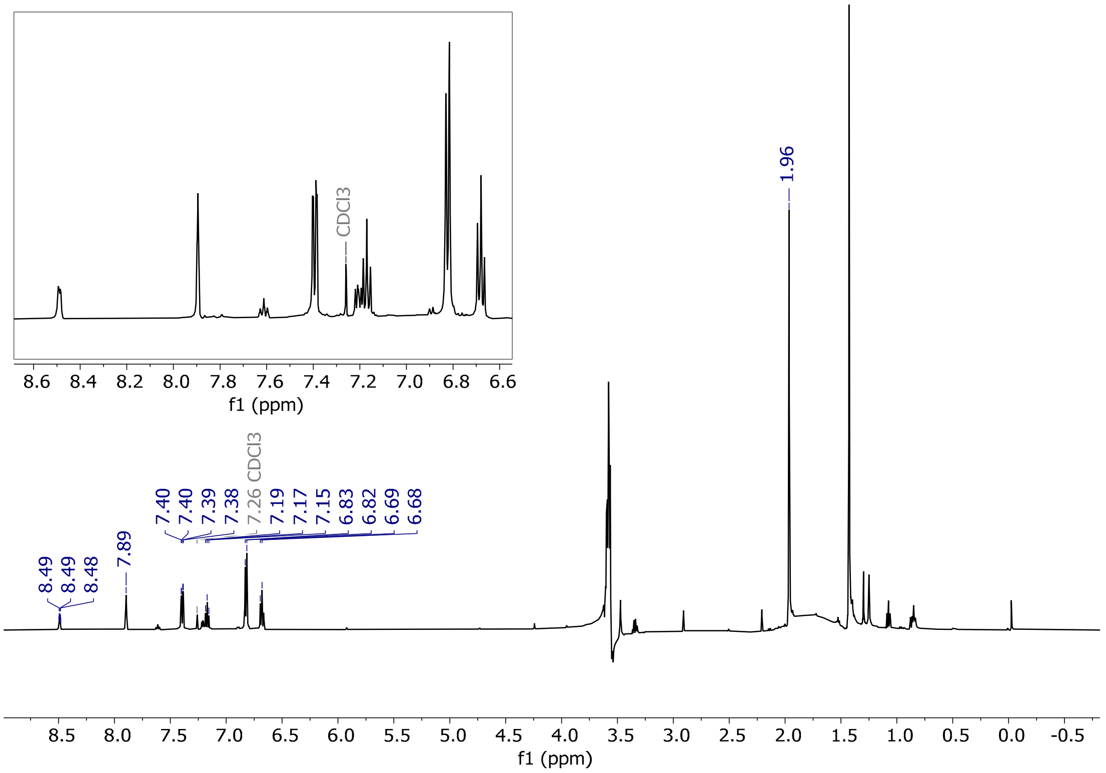

Figure S28. ^{1}H NMR spectrum of the reaction of **5Xyl-HLi$_2$** and Bi(OTf)$_3$ in thf-h_8. Calibrated to residual $CHCl_3$. All signals detected in the aromatic region can be assigned to **5Xyl-H$_3$**.

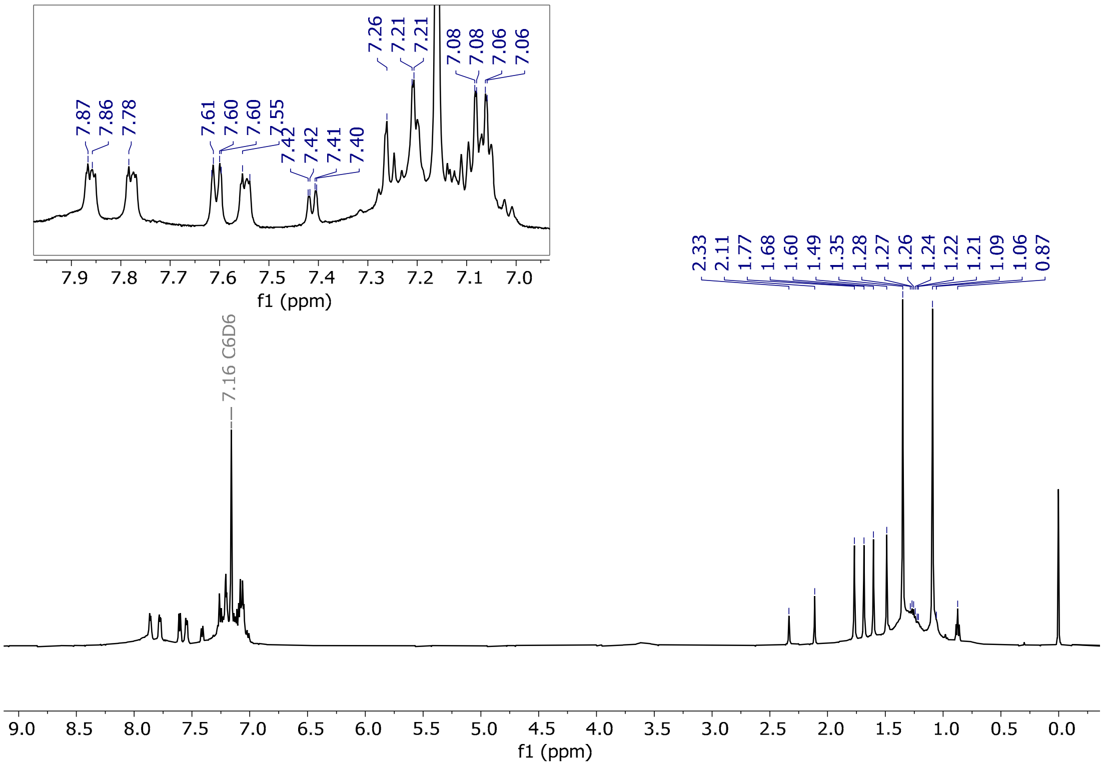

Figure S29. ^{1}H NMR spectrum of the reaction of **5TBP-HLi$_2$**, 1.1 eq. MeLi and 1 eq. $BiBr_3$ after work-up in C_6D_6.

Characterization data

Crystallographic data

Table S1. Crystal data and structure refinement for **5Xyl-H$_3$·HCl**.

Compound	**5Xyl-H$_3$·HCl · EtOH**
Empirical formula	$C_{58}H_{82}Cl_4N_4O$
Formula weight	993.07
Temperature/K	150.00
Wavelength/Å	0.71073
Crystal system	monoclinic
Space group	C 1 2/c 1
a/Å	31.4346(9)
b/Å	9.8966(3)
c/Å	17.8171(5)
α/°	90
β/°	93.4590(10)
γ/°	90
Volume/ Å^3	5532.7(3)
Z	4
ρ_{calc}/ mg/m^3	1.192
μ/ mm^{-1}	0.256
F(000)	2136
Crystal size/ mm^3	0.35 x 0.28 x 0.15
Theta range for data collection/°	2.158 to 36.327
Index ranges	-52<=h<=52, -16<=k<=16, -29<=l<=29
Reflections collected	123460
Independent reflections	13416 [R(int) = 0.0301]
Completeness to theta = 25.242°	100.0 %
Absorption correction	Semi-empirical from equivalents
Max. and min. transmission	0.7471 and 0.6938
Refinement method	Full-matrix least-squares on F2

Data / restraints / parameters	13416 / 5 / 339
Goodness-of-fit on F2	1.074
Final R indices [I>2σ (I)]	R1 = 0.0419, wR2 = 0.1103
R indices (all data)	R1 = 0.0539, wR2 = 0.1264
Extinction coefficient	n/a
Largest diff. peak and hole/ e $Å^{-3}$	0.502 and -0.493

Mass spectra

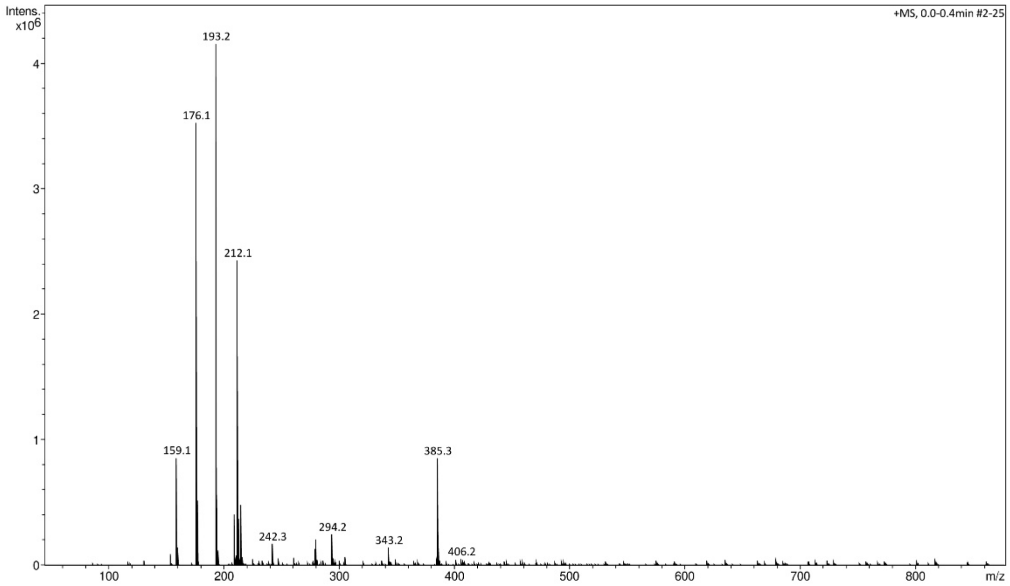

Figure S30. ESI(+) mass spectrum of **5TMS-H$_3$** in the range from 50 to 1000 m/z. Compound **5TMS-H$_3$** could not be detected. The dominant peak at 193.2 m/z most likely corresponds to the hydrolysis product **5H-H$_3$**.

Acquisition Parameter

Source Type	ESI	Ion Polarity	Positive	Set Nebulizer	0.5 Bar
Focus	Not active	Set Capillary	3500 V	Set Dry Heater	180 °C
Scan Begin	50 m/z	Set End Plate Offset	-500 V	Set Dry Gas	4.0 l/min
Scan End	1500 m/z	Set Charging Voltage	2000 V	Set Divert Valve	Source
		Set Corona	0 nA	Set APCI Heater	0 °C

Meas. m/z	Ion Formula	m/z	err [ppm]
505.4374	C30H61N2Si2	505.4368	-1.3

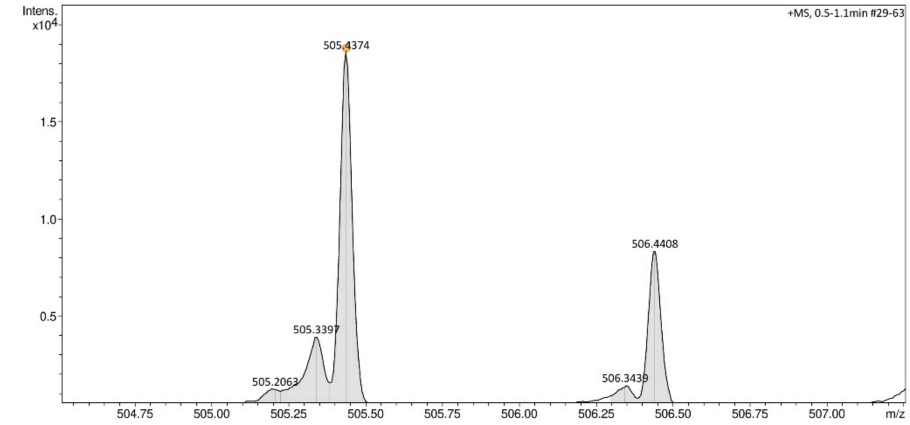

Figure S31. Calibrated high-resolution ESI(+) mass spectrum of **5TIPS-H$_3$**.

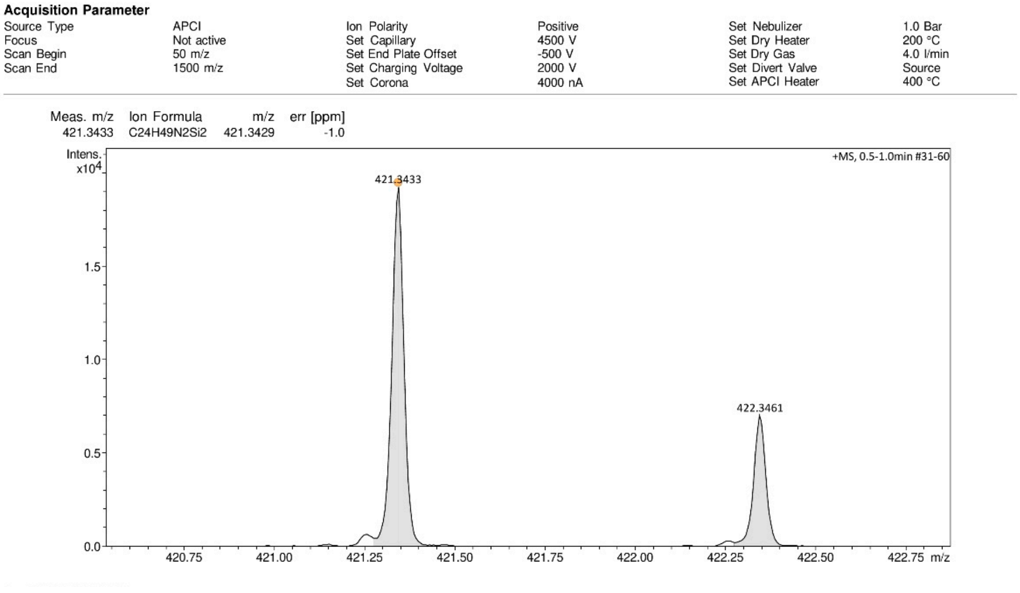

Figure S32. Calibrated high-resolution APCI(+) mass spectrum of **5TES-H$_3$.**

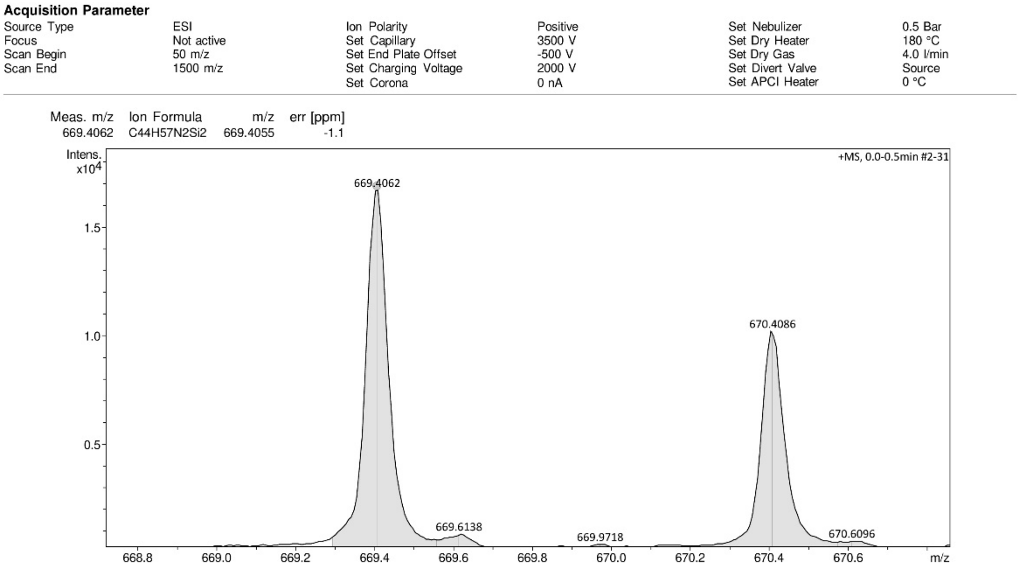

Figure S33. Calibrated high-resolution APCI(+) mass spectrum of **5TBP-H$_3$.**

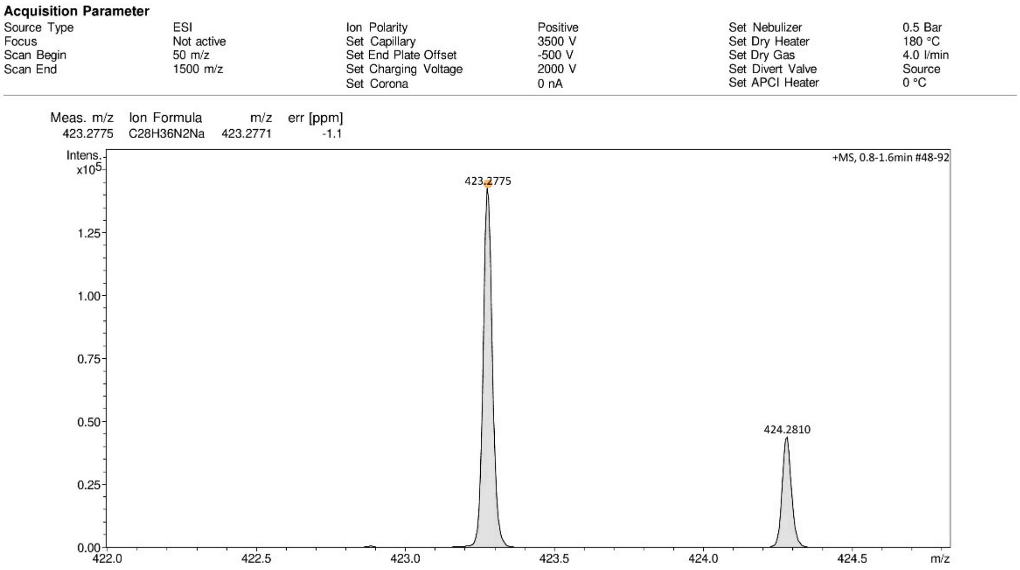

Figure S34. Calibrated high-resolution ESI(+) mass spectrum of **5Xyl-H$_3$**.

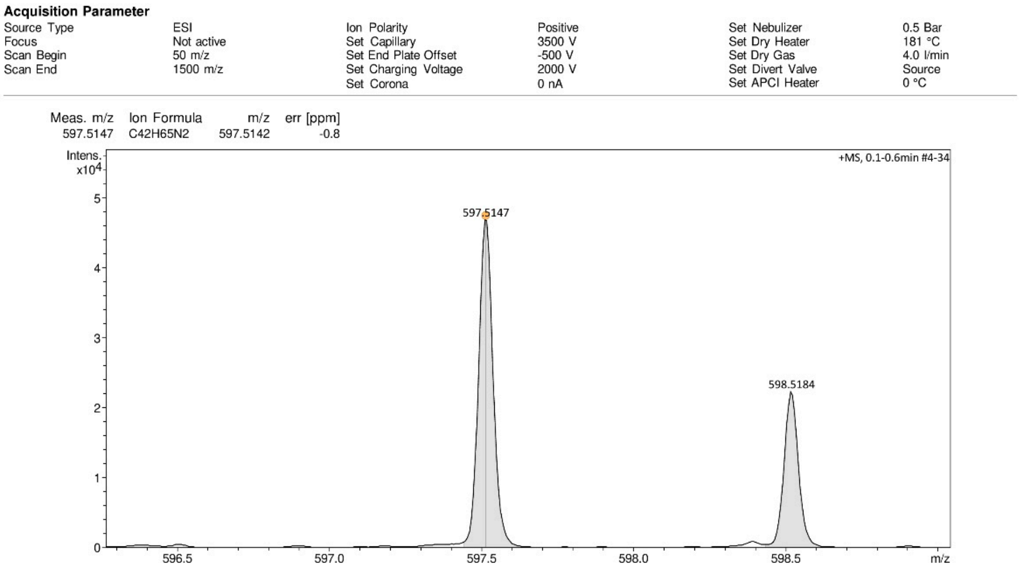

Figure 35. Calibrated high-resolution ESI(+) mass spectrum of **5Tripp-H$_3$**.

NMR spectra

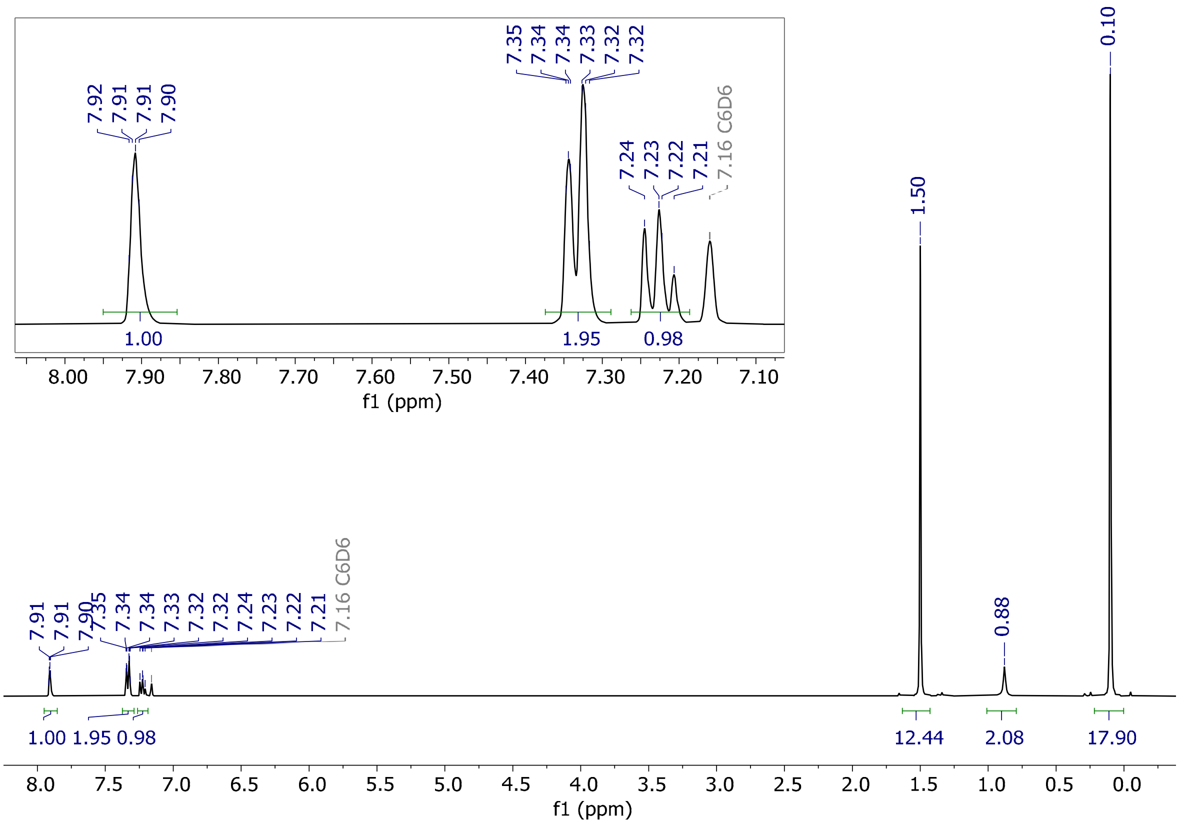

Figure S36. ^{1}H NMR spectrum of **5TMS-H$_3$** in C_6D_6.

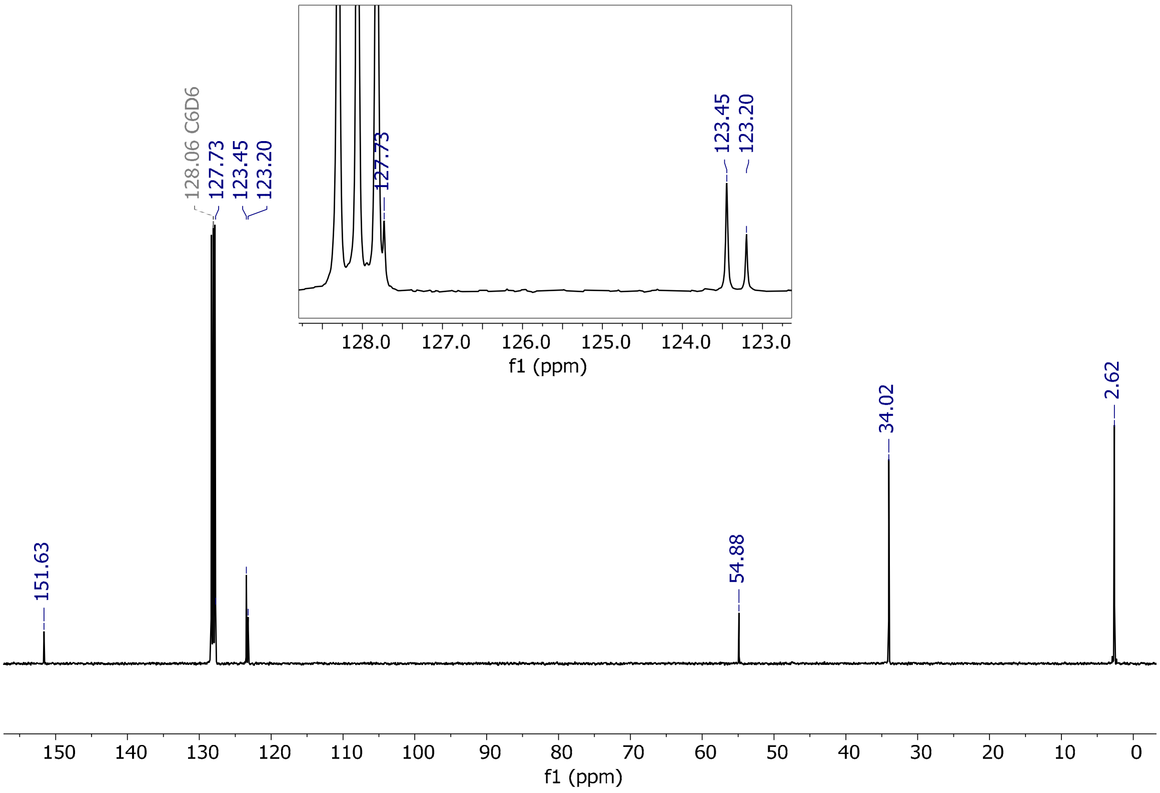

Figure S37. ^{13}C{^{1}H} NMR spectrum of **5TMS-H$_3$** in C_6D_6.

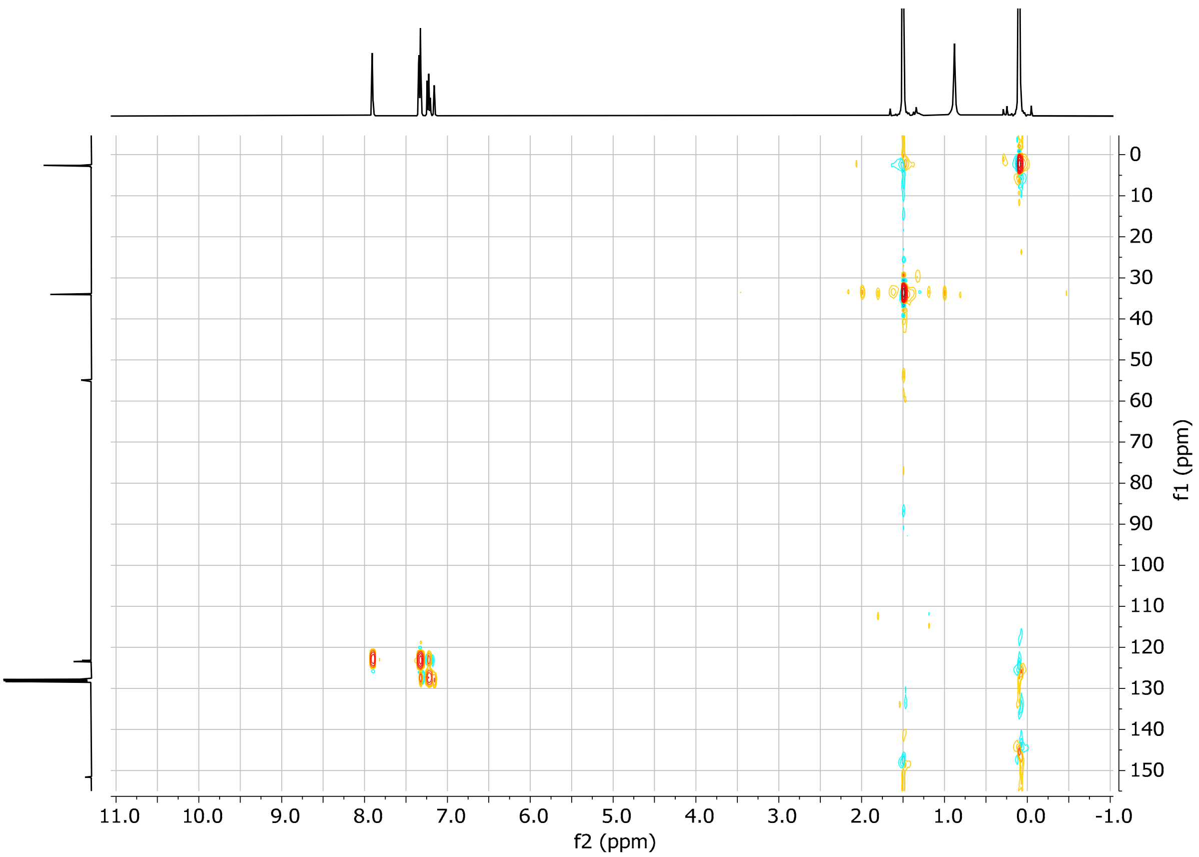

Figure S38. $^{1}H^{13}C$ HSQC NMR spectrum of **5TMS-H₃** in C_6D_6.

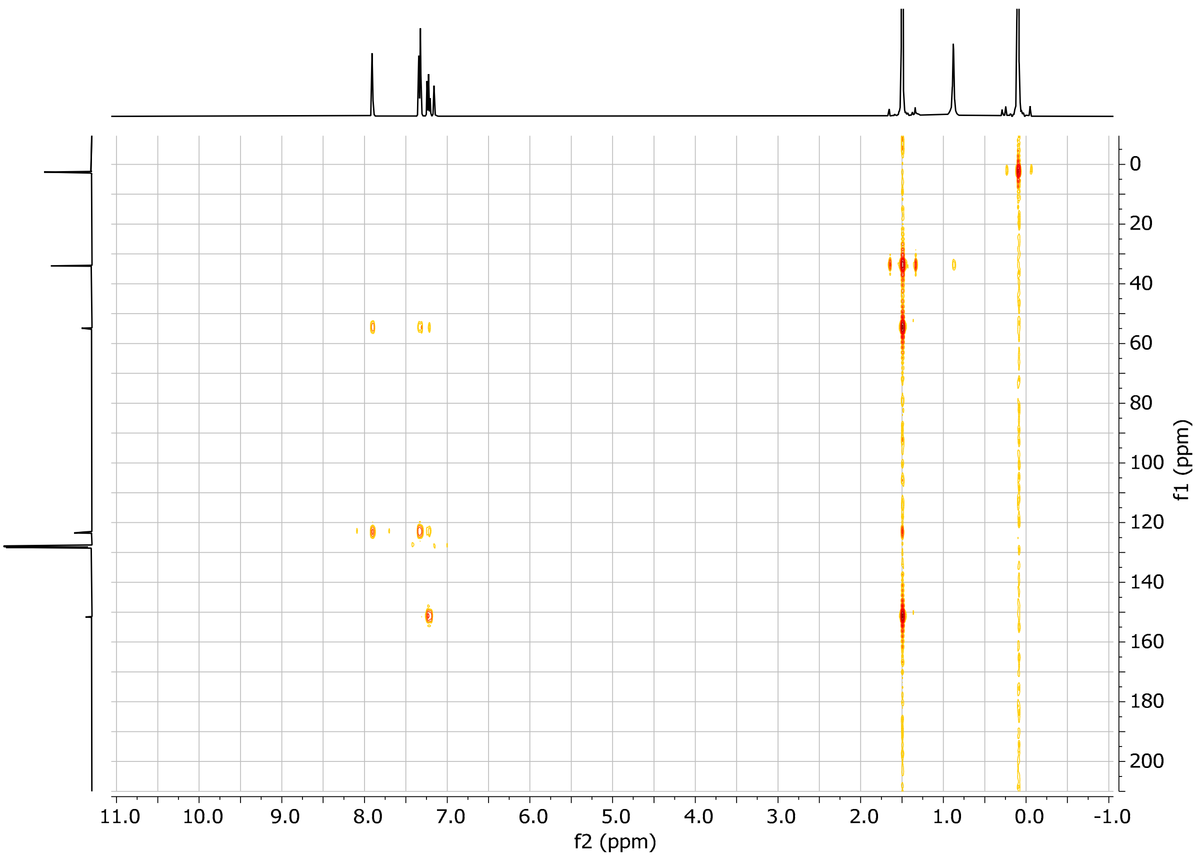

Figure S39. $^{1}H^{13}C$ HMBC NMR spectrum of **5TMS-H₃** in C_6D_6.

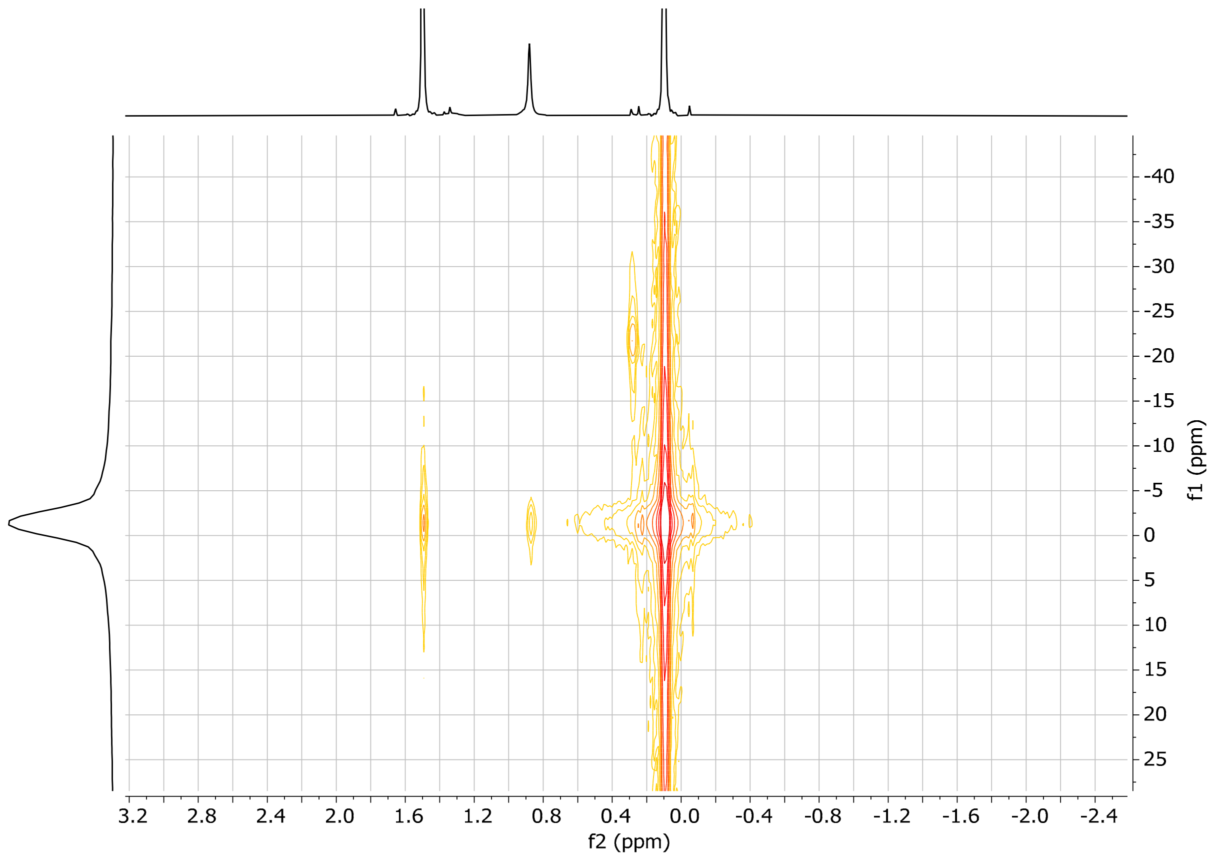

Figure S40. $^{1}H^{29}Si$ HMBC NMR spectrum of **5TMS-H$_3$** in C_6D_6.

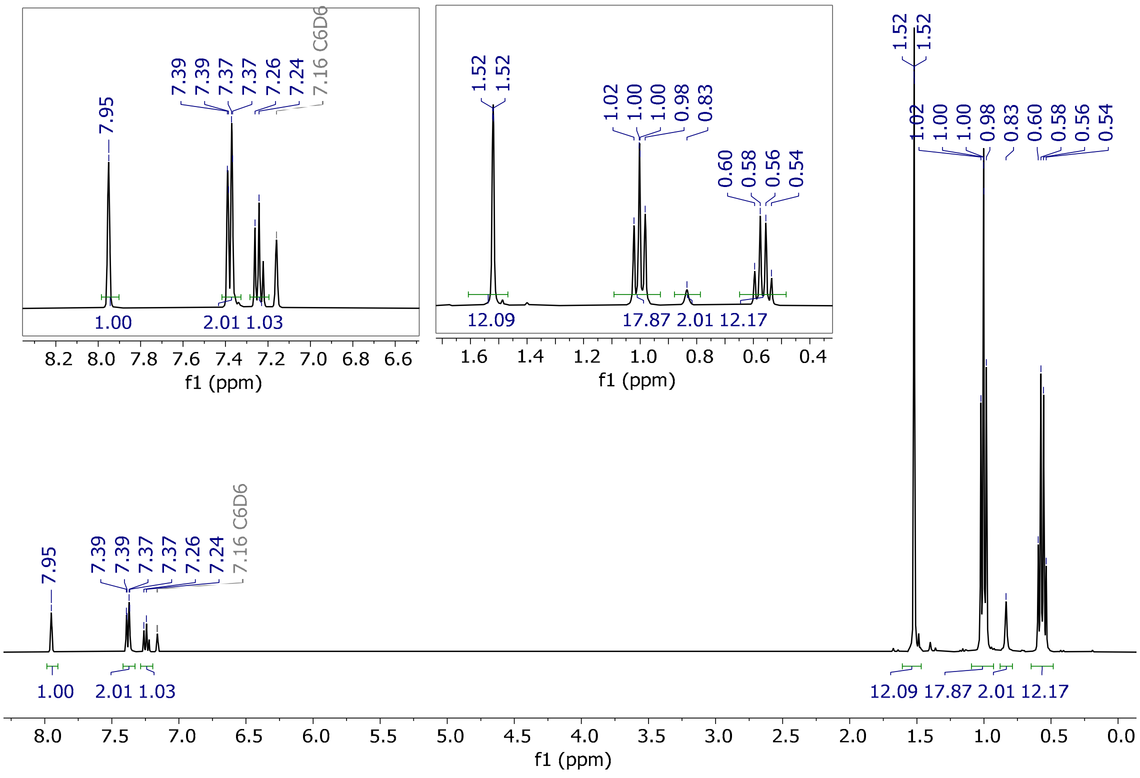

Figure S41. ^{1}H NMR spectrum of **5TES-H$_3$** in C_6D_6.

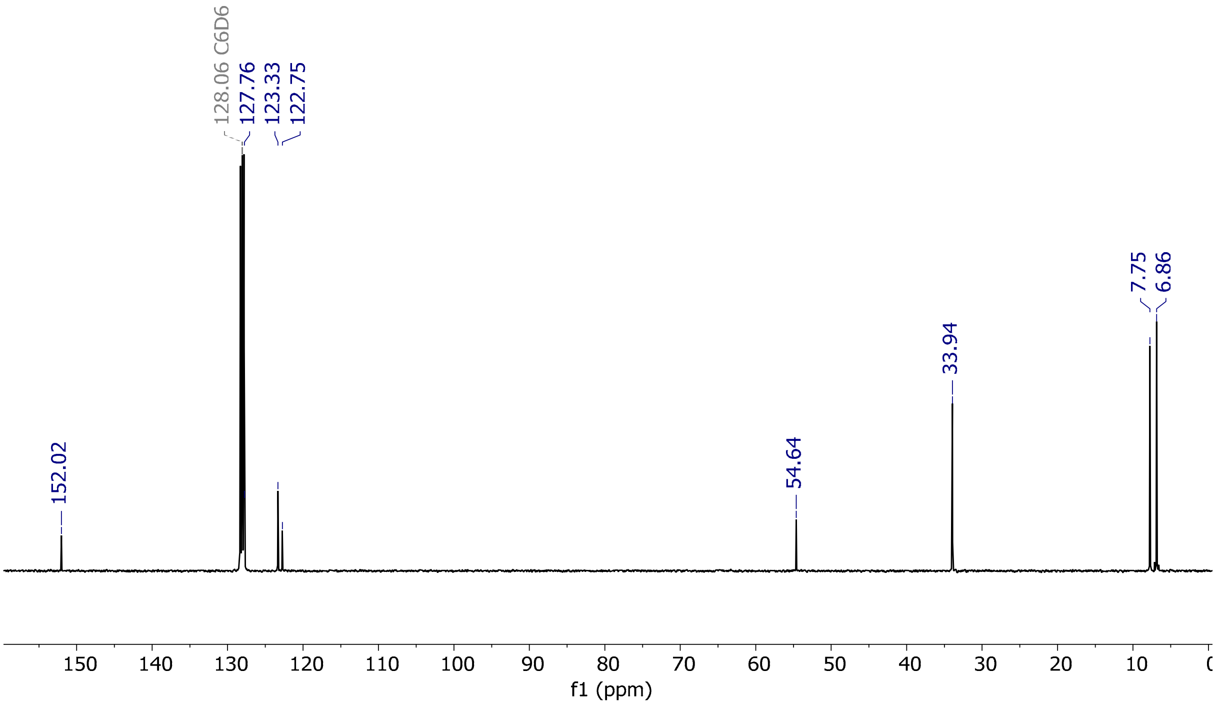

Figure S42. $^{13}C\{^{1}H\}$ NMR spectrum of **5TES-H$_3$** in C_6D_6.

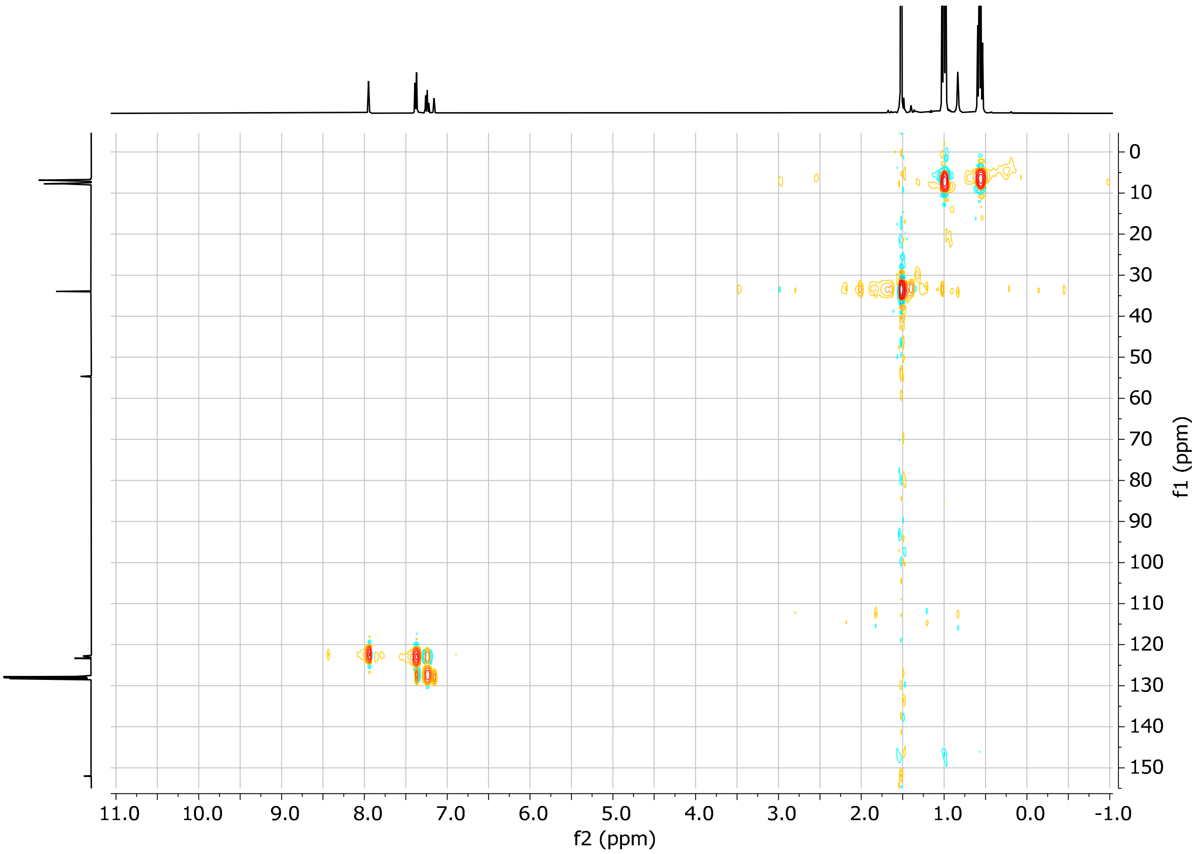

Figure S43. $^{1}H^{13}C$ HSQC NMR spectrum of **5TES-H$_3$** in C_6D_6.

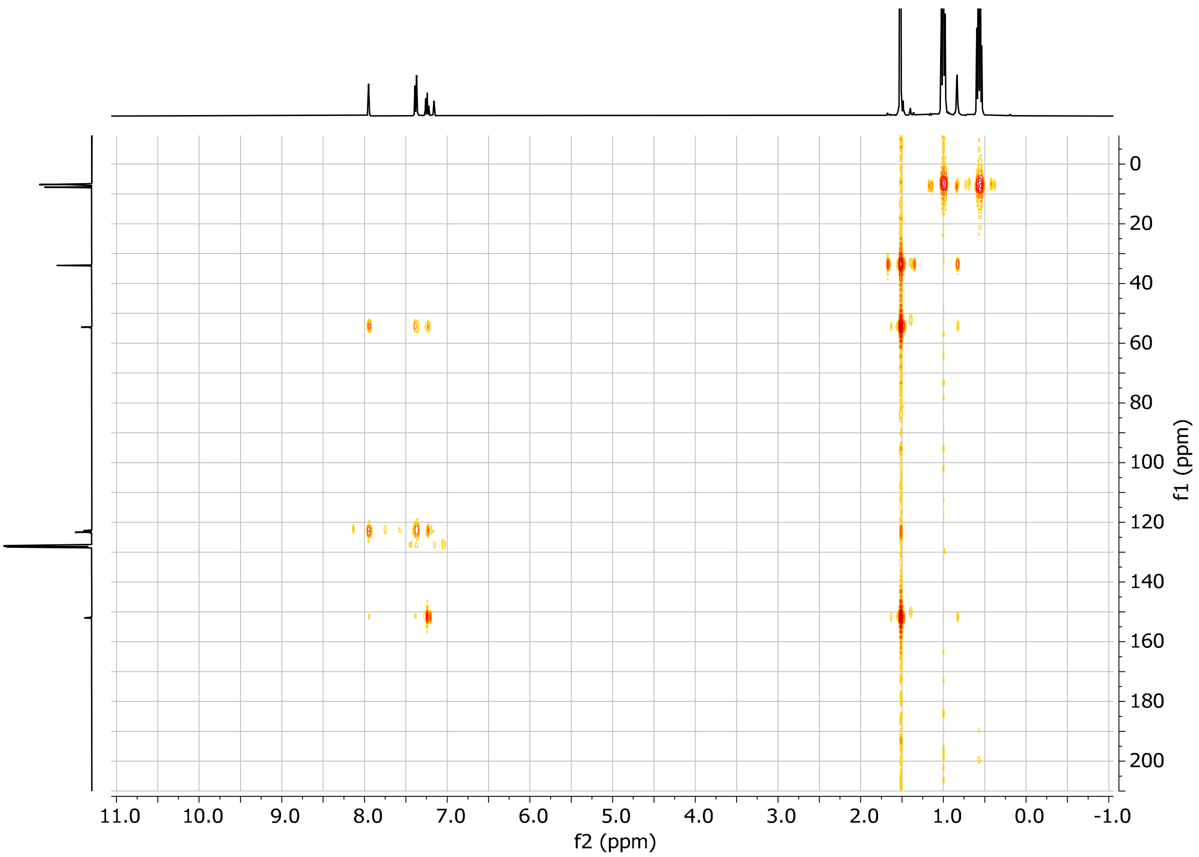

Figure S44. $^{1}H^{13}C$ HMBC NMR spectrum of **5TES-H$_3$** in C_6D_6.

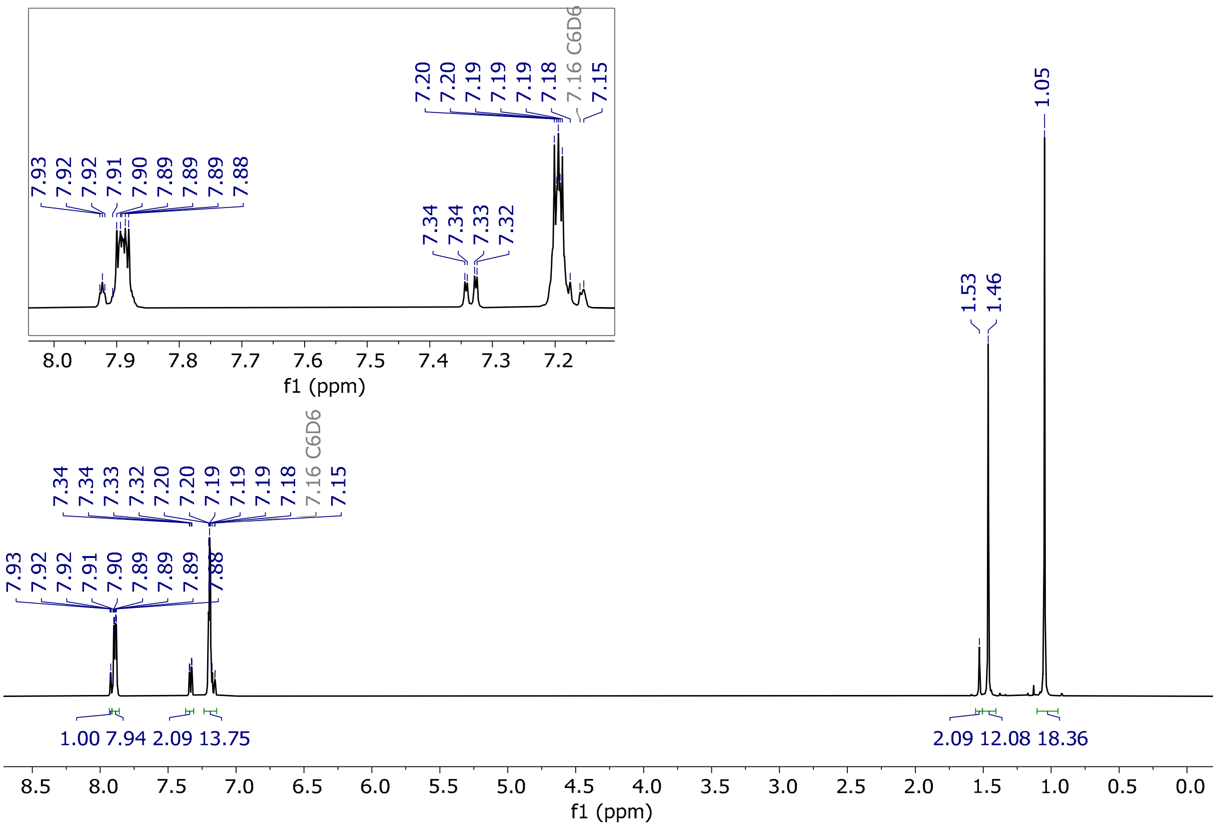

Figure S45. ^{1}H NMR spectrum of **5TBP-H$_3$** in C_6D_6.

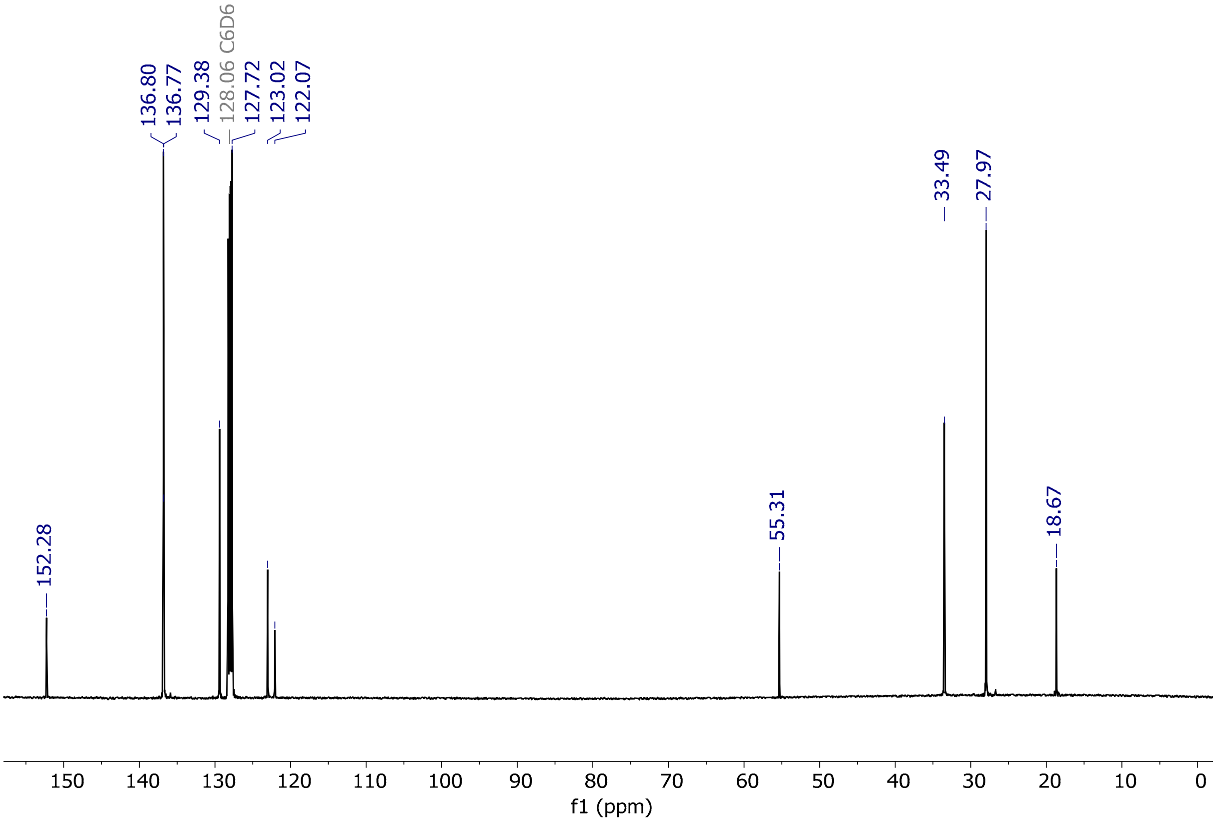

Figure S46. $^{13}C\{^1H\}$ NMR spectrum of **5TBP-H$_3$** in C_6D_6.

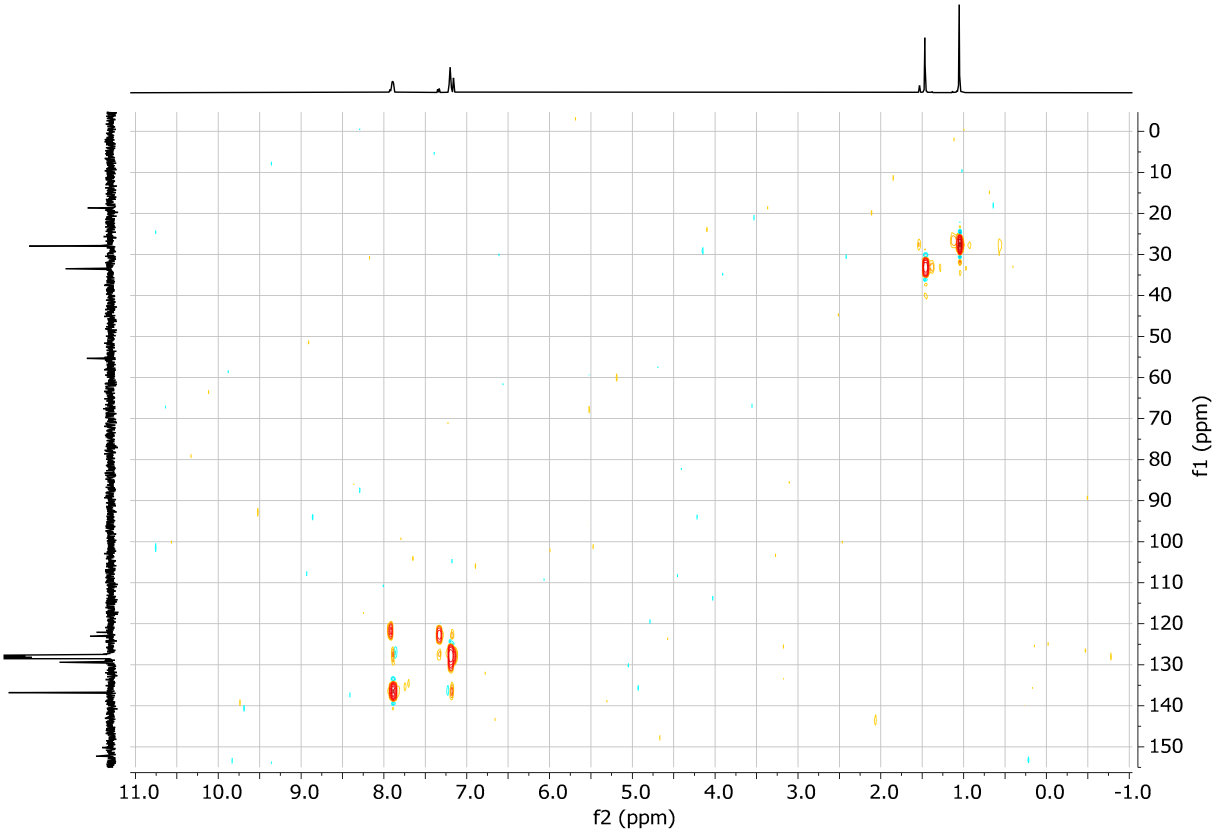

Figure S47. $^1H^{13}C$ NMR HSQC spectrum of **5TBP-H$_3$** in C_6D_6.

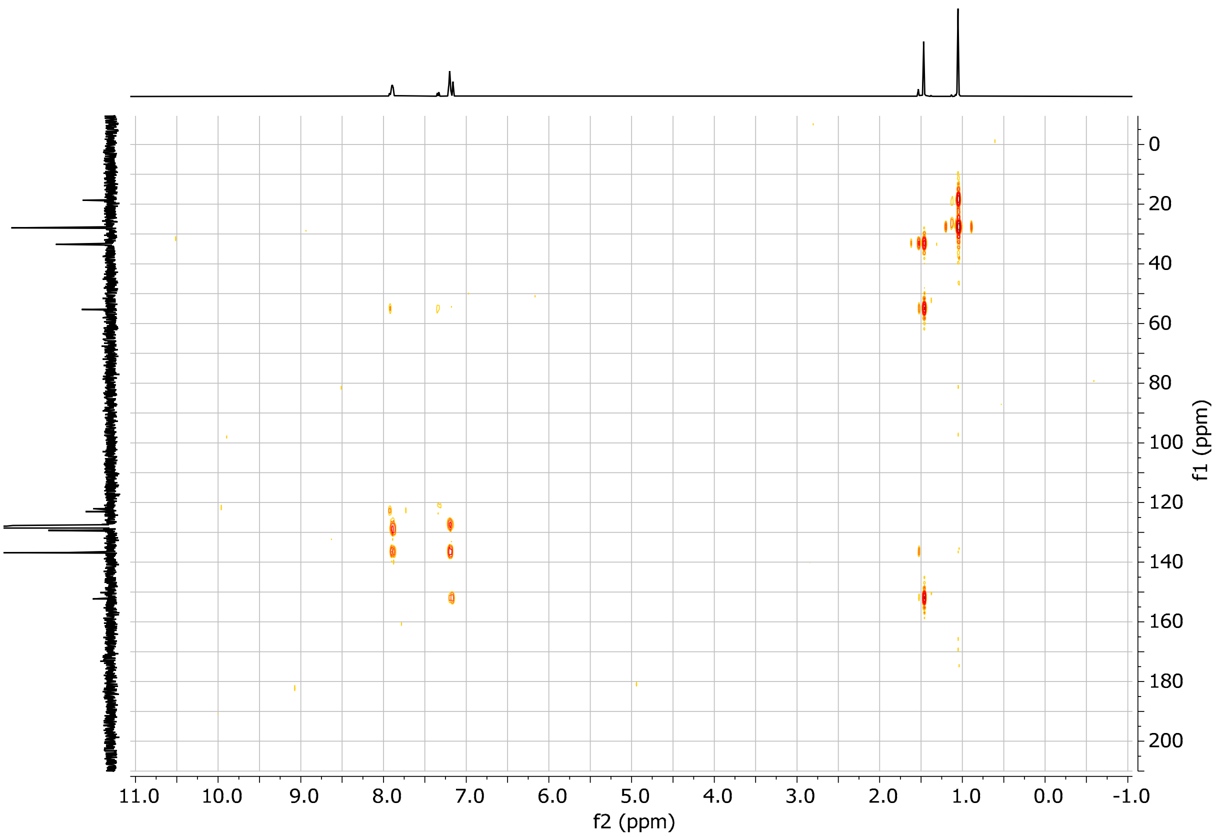

Figure S48. $^{1}H^{13}C$ NMR HMBC spectrum of **5TBP-H$_3$** in C_6D_6.

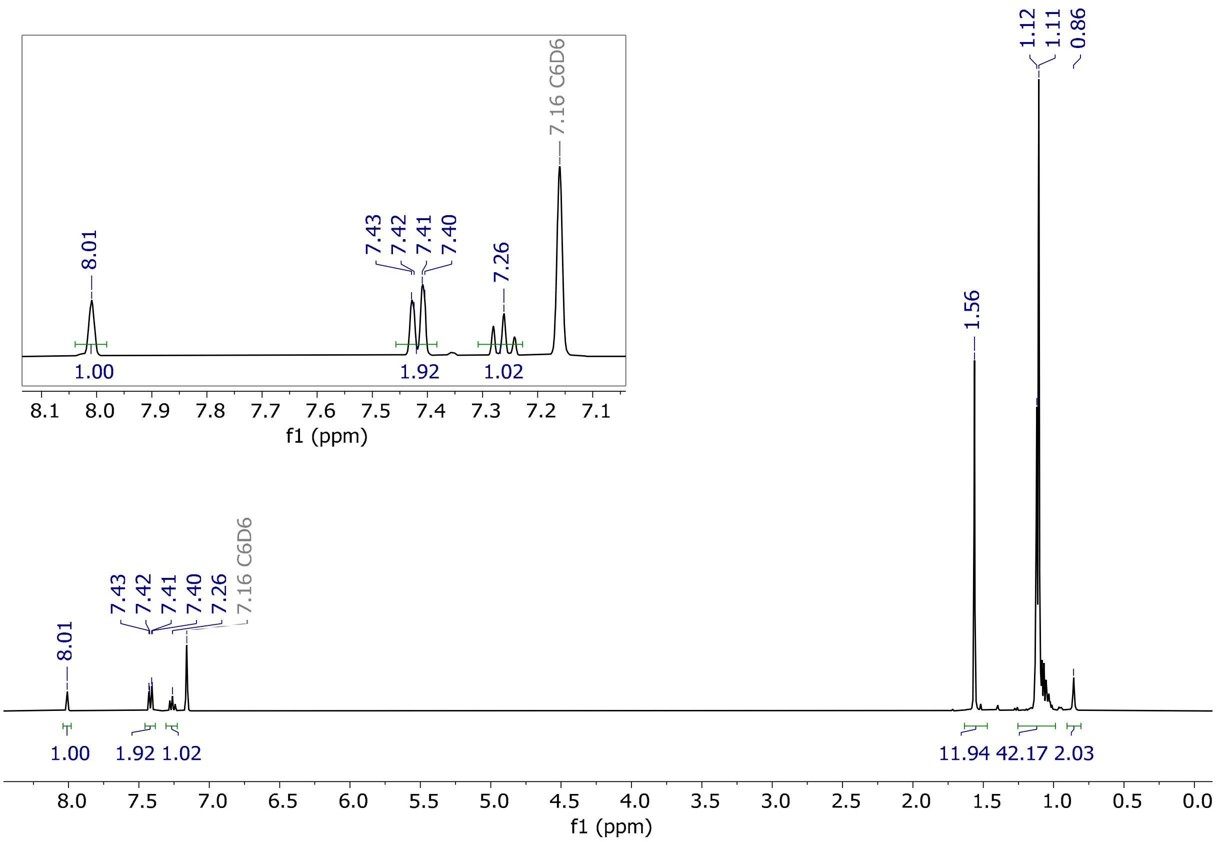

Figure S49. ^{1}H NMR spectrum of **5TIPS-H$_3$** in C_6D_6.

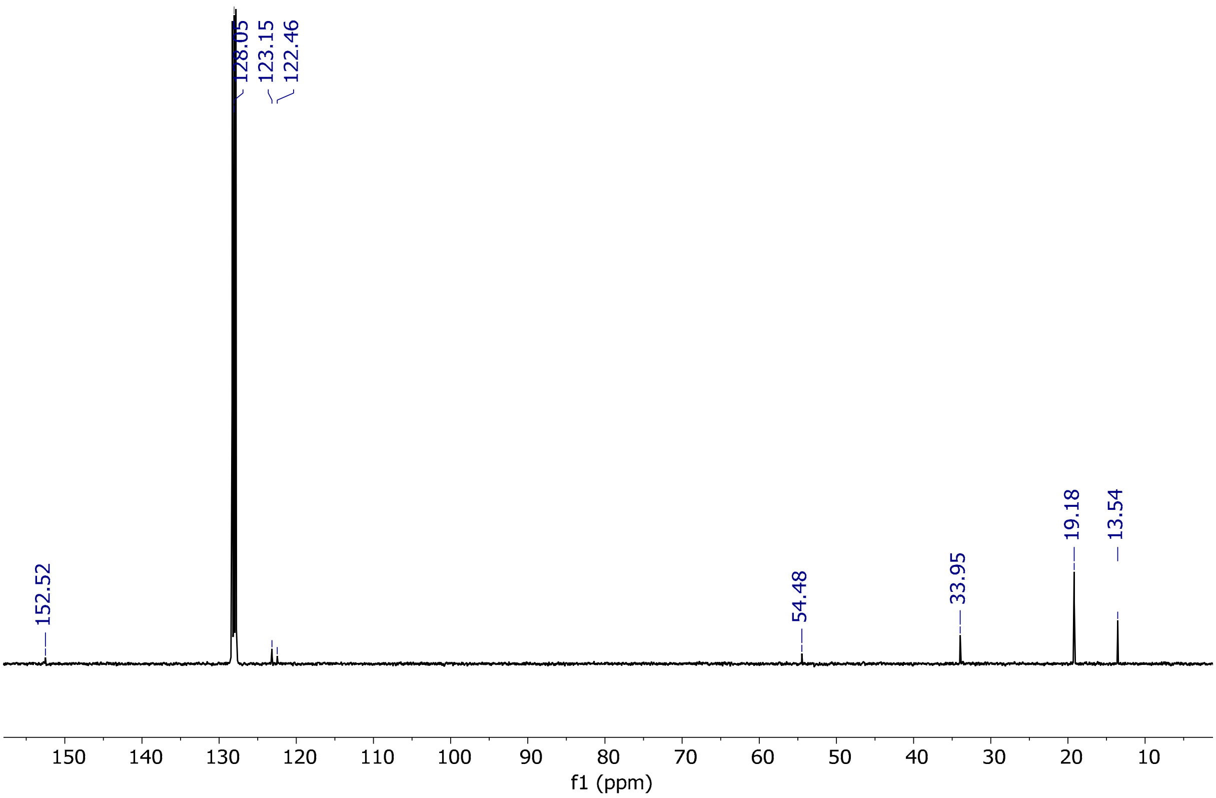

Figure S50. $^{13}C\{^{1}H\}$ NMR spectrum of **5TIPS-H$_3$** in C_6D_6.

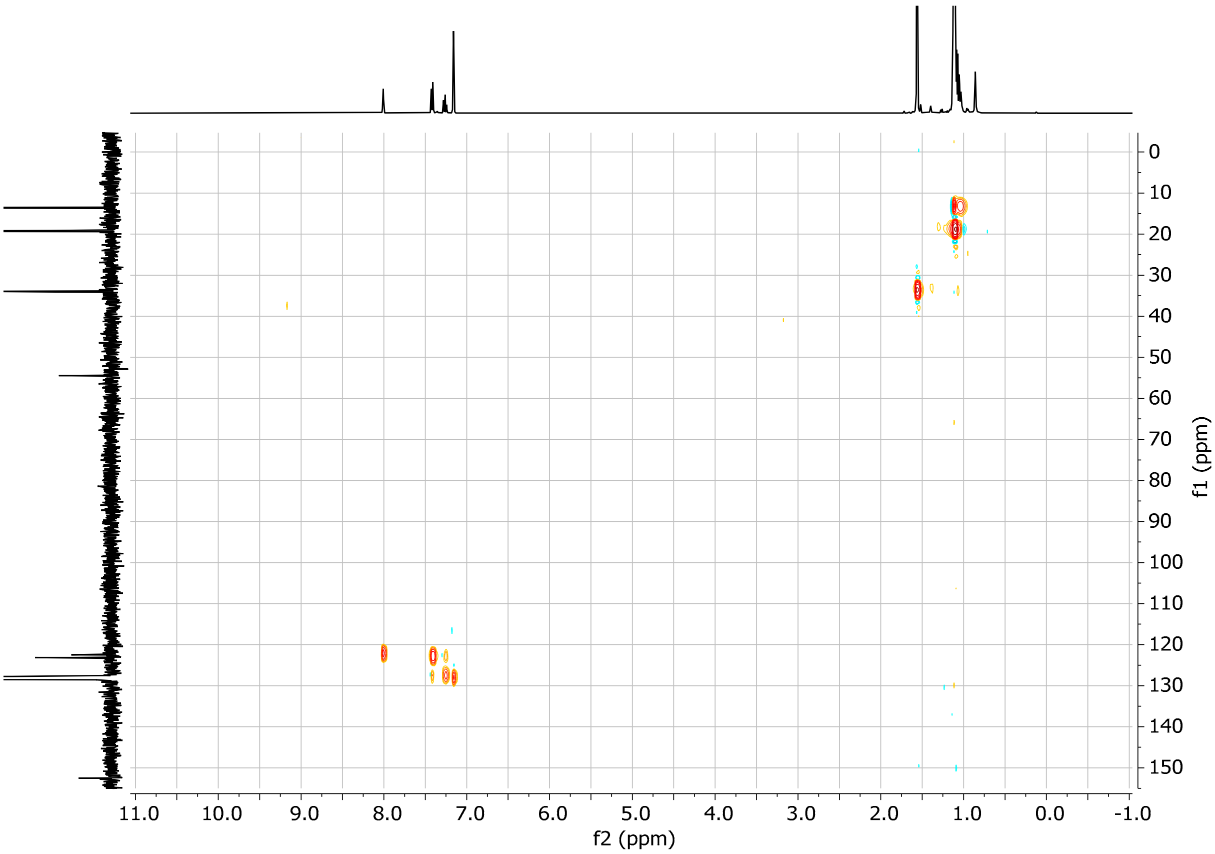

Figure S51. $^{1}H^{13}C$ HSQC NMR spectrum of **5TIPS-H$_3$** in C_6D_6.

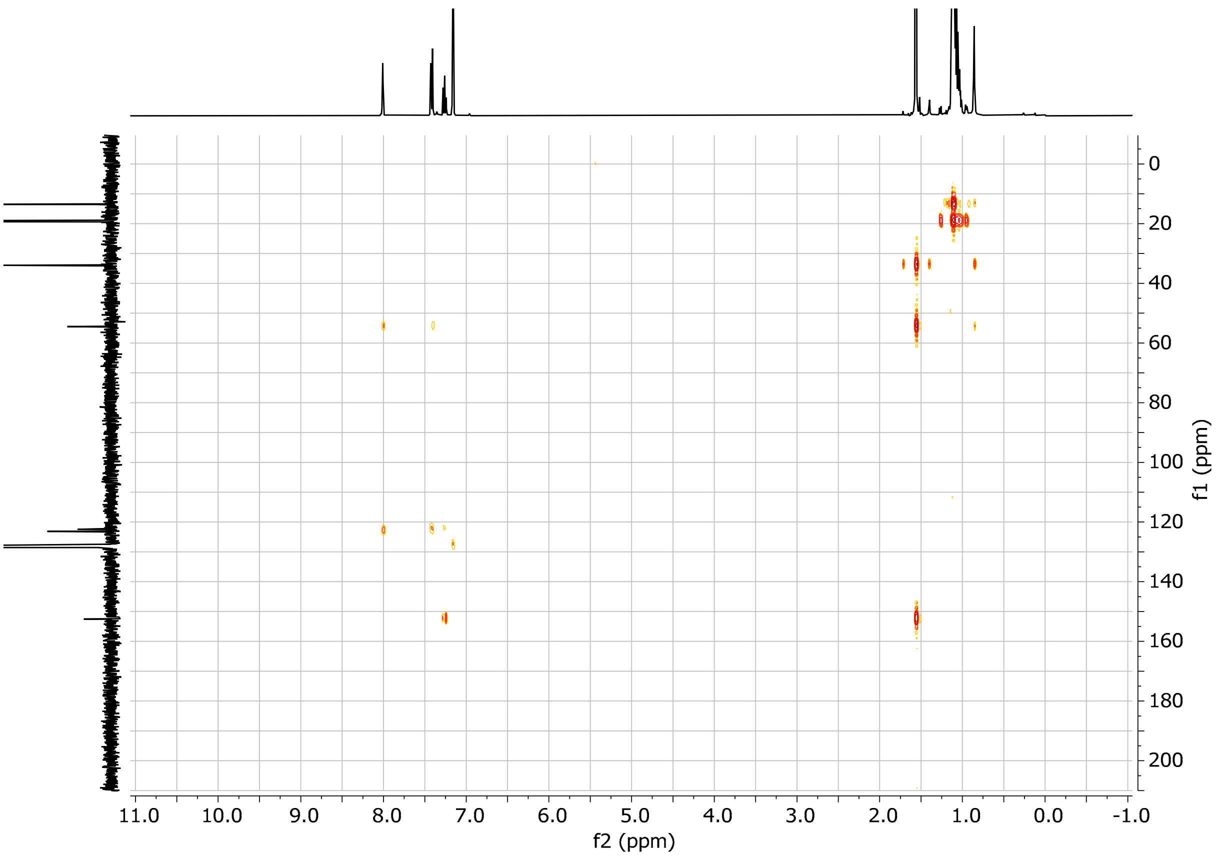

Figure S52. $^{1}H^{13}C$ HMBC NMR spectrum of **5TIPS-H$_3$** in C_6D_6.

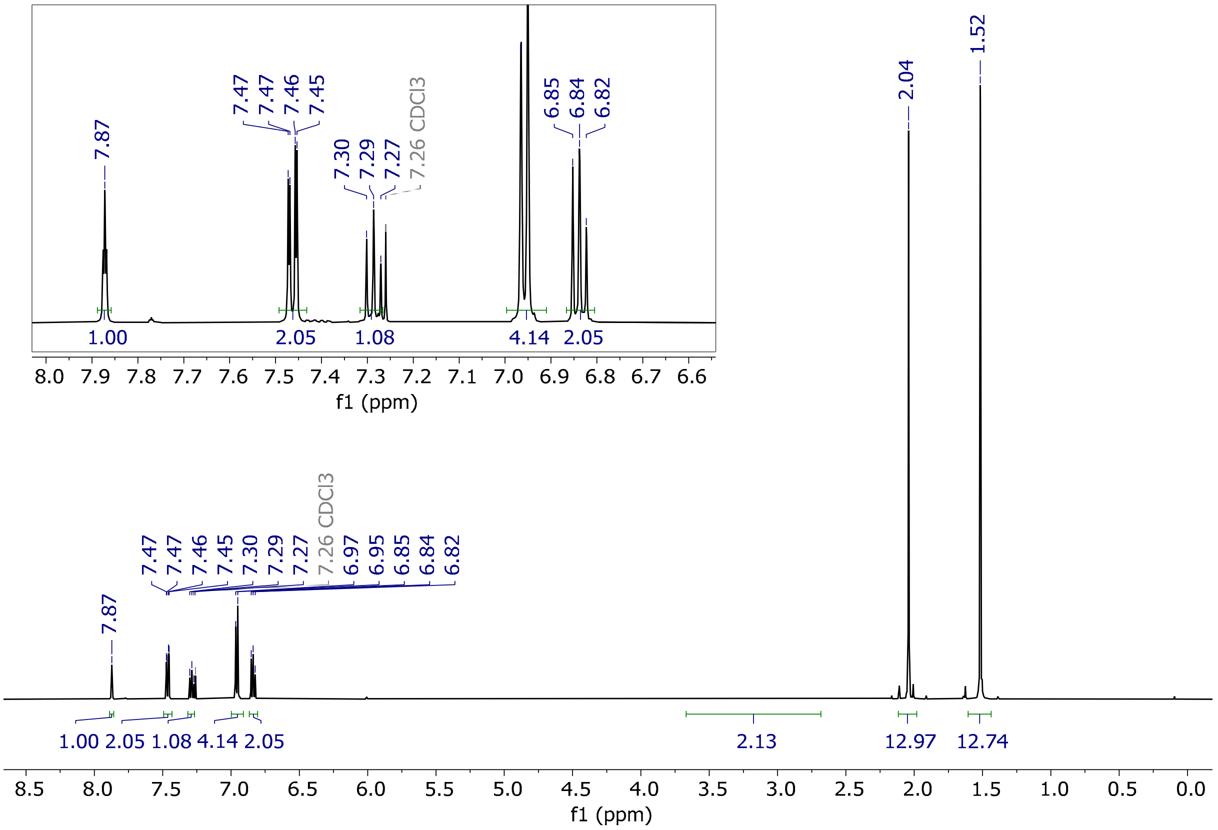

Figure S53. ^{1}H NMR spectrum of **5Xyl-H$_3$** in $CDCl_3$.

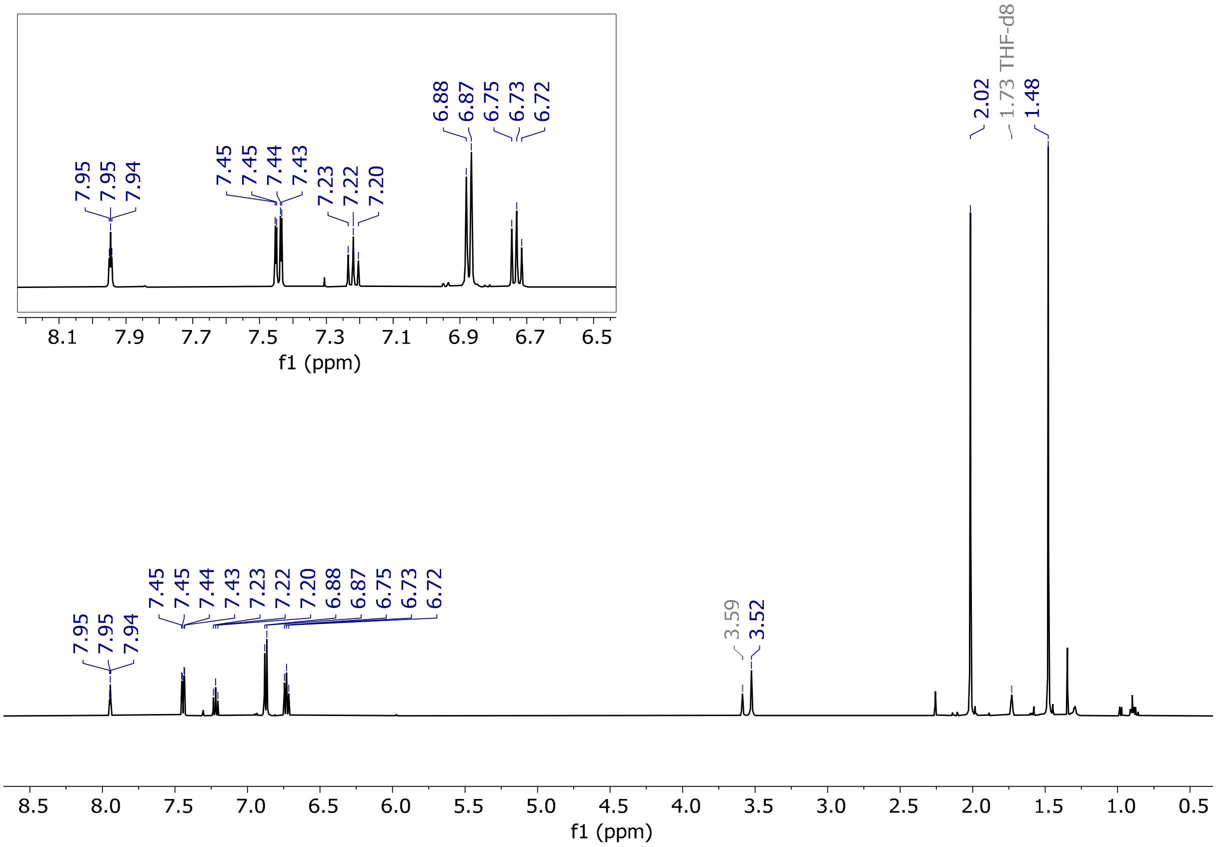

Figure S54. ^{1}H NMR spectrum of **5Xyl-H$_3$** in thf-d_8.

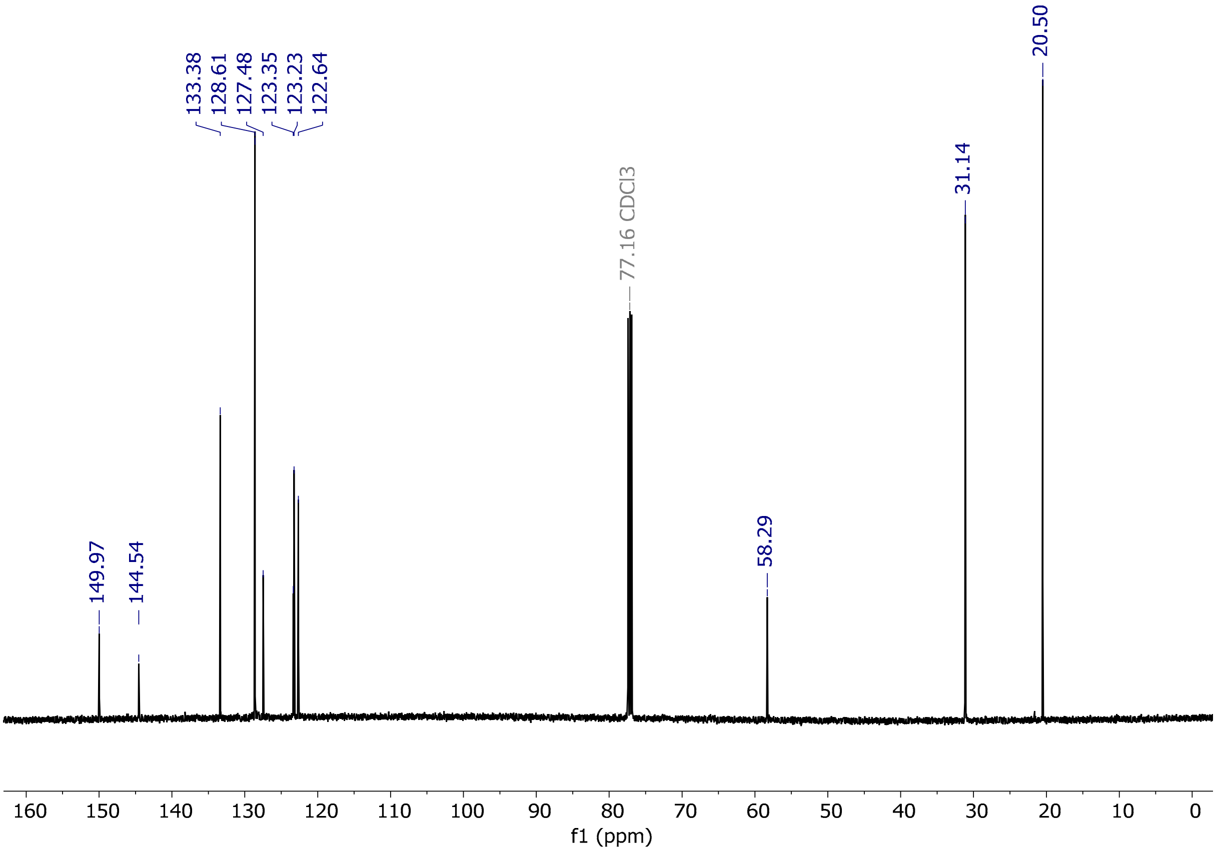

Figure S55. ^{13}C{^{1}H} NMR spectrum of **5Xyl-H$_3$** in CDCl$_3$.

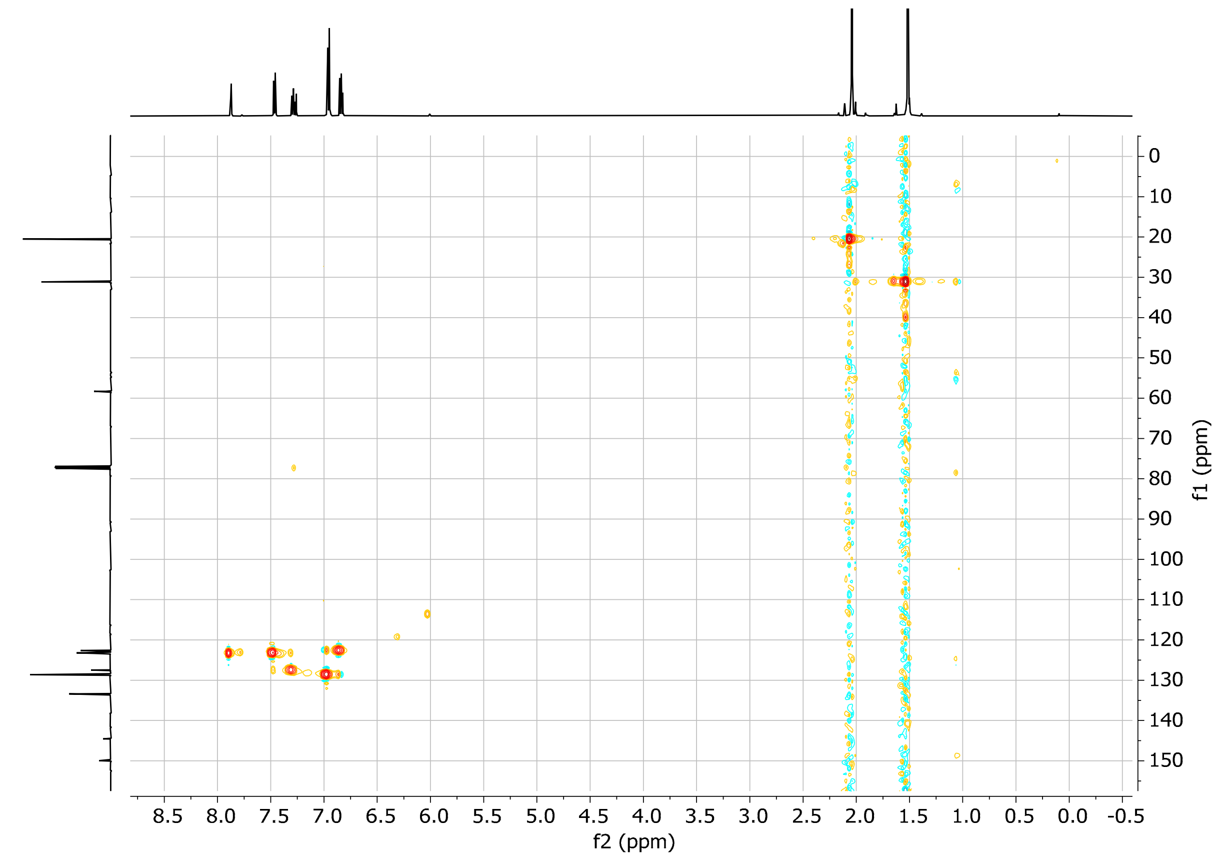

Figure S56. $^{1}H^{13}C$ HSQC NMR spectrum of **5Xyl-H$_3$** in $CDCl_3$.

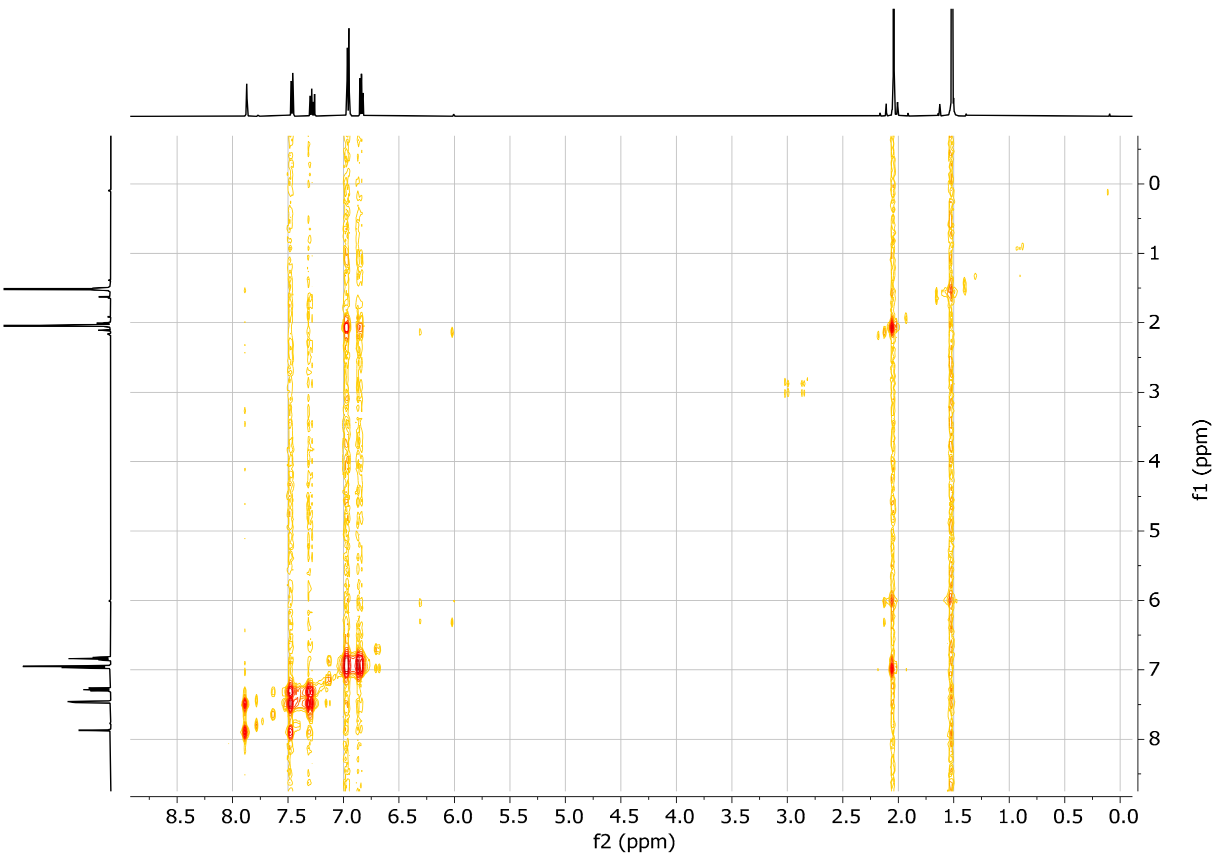

Figure S57. $^{1}H^{1}H$ COSY NMR spectrum of **5Xyl-H$_3$** in $CDCl_3$.

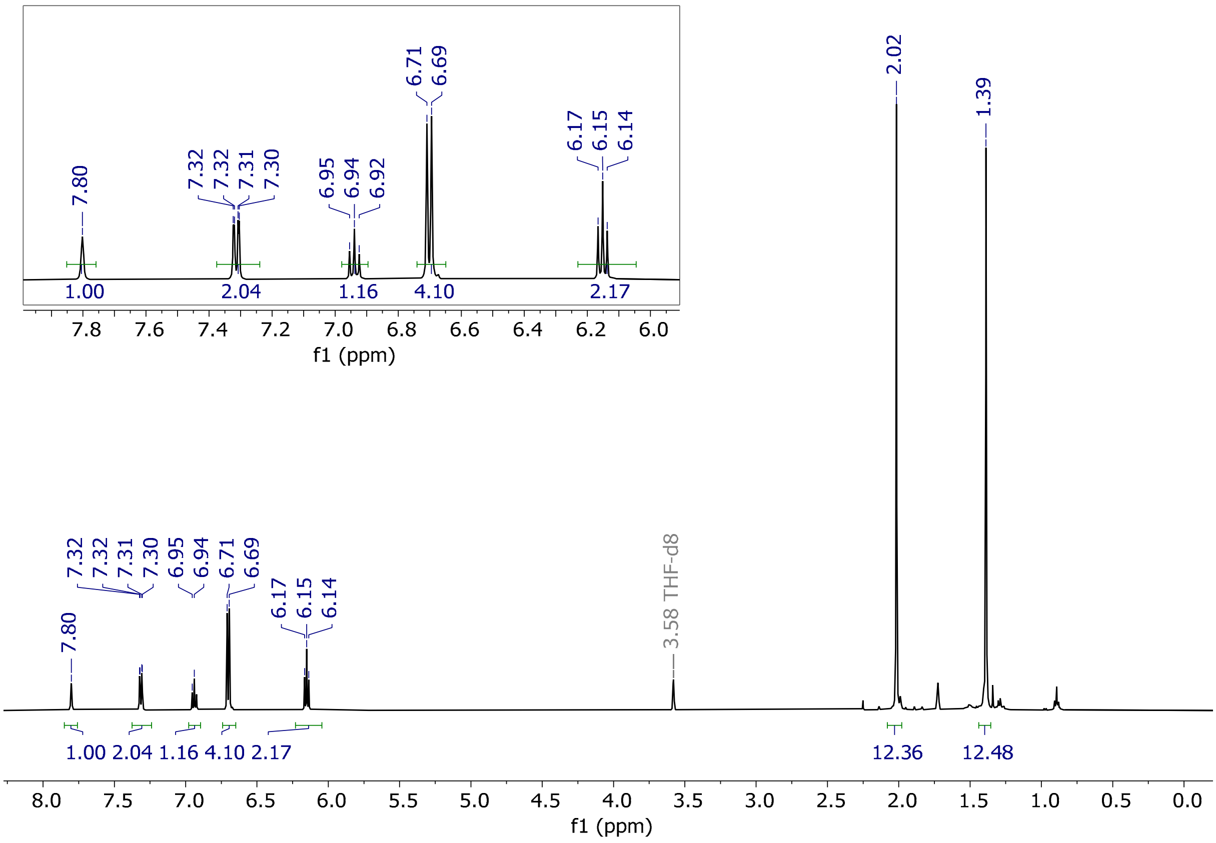

Figure S58. ^{1}H NMR spectrum of **5Xyl-HLi₂** in thf-d_8.

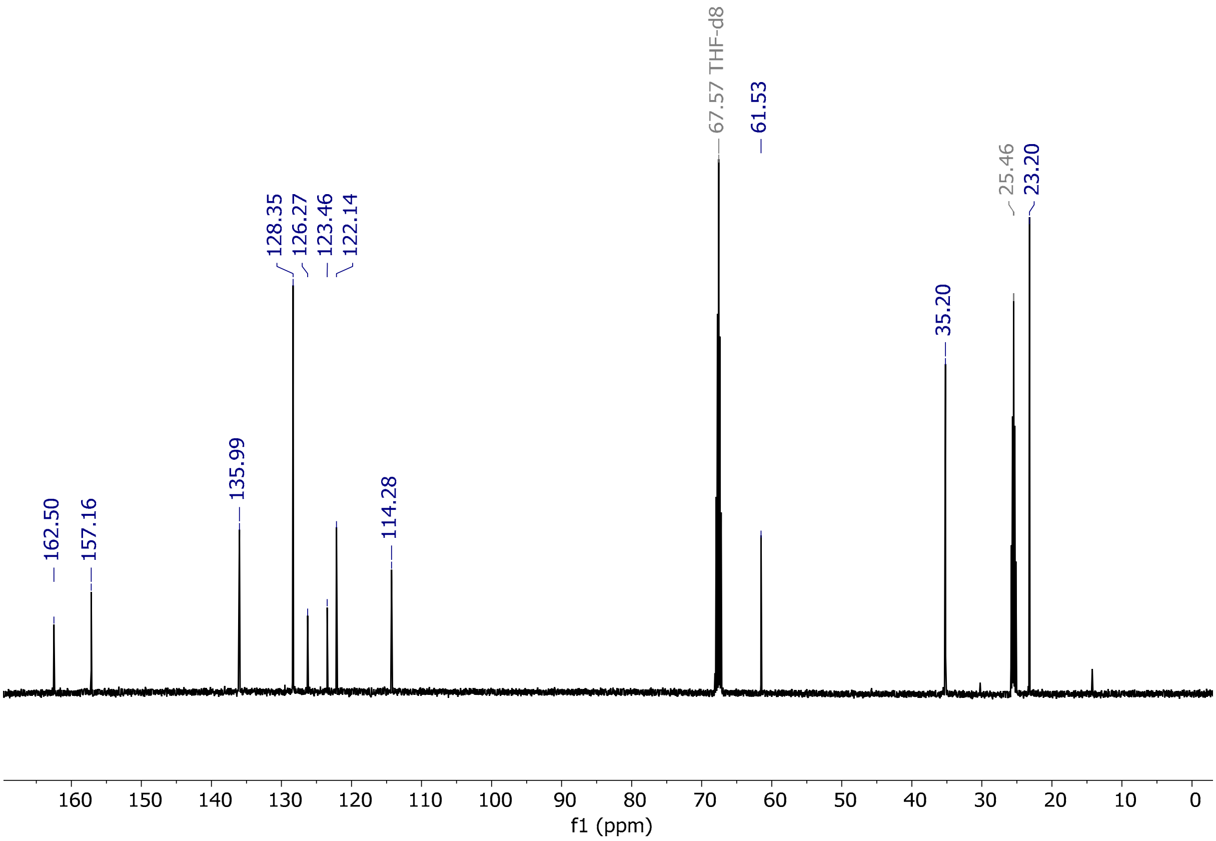

Figure S59. $^{13}C\{^{1}H\}$ NMR spectrum of **5Xyl-HLi₂** in thf-d_8.

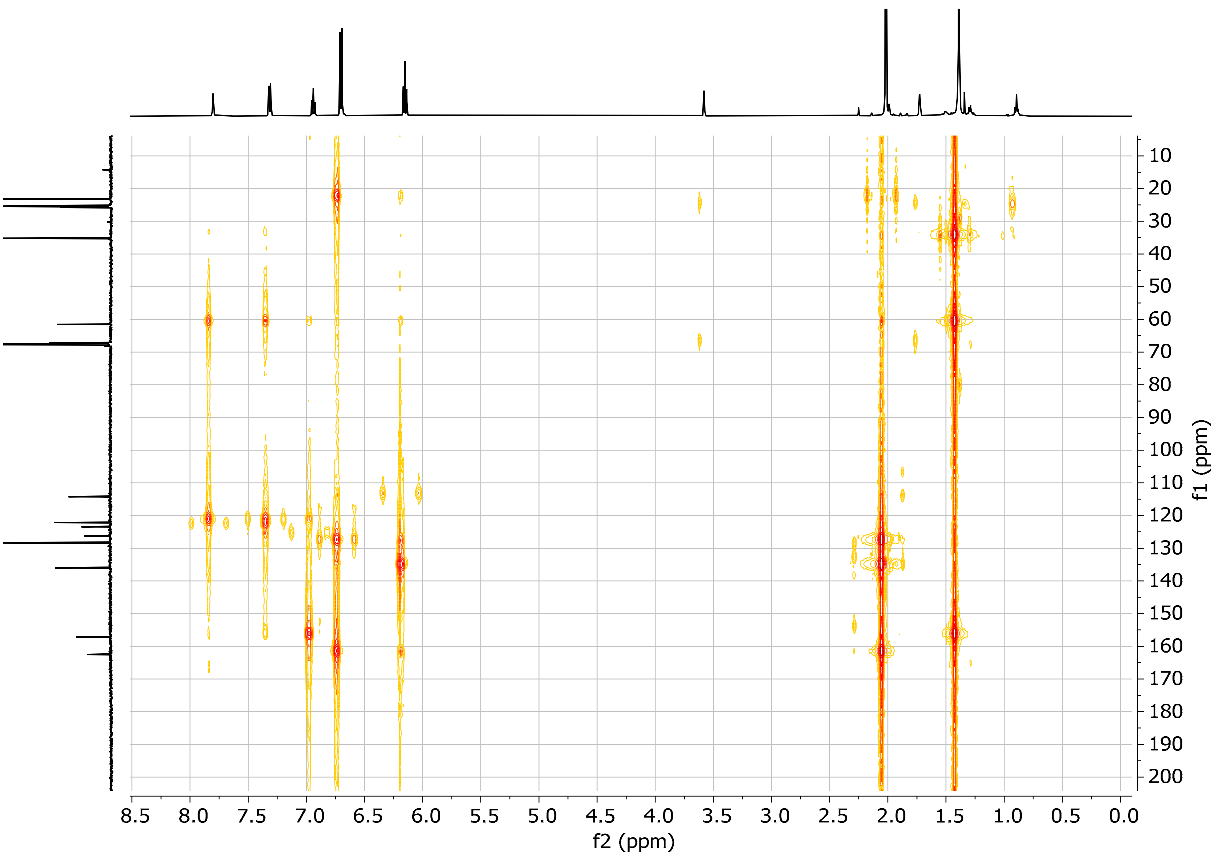

Figure S60. $^{1}H^{13}C$ HMBC NMR spectrum of **5Xyl-HLi$_2$** in thf-d_8.

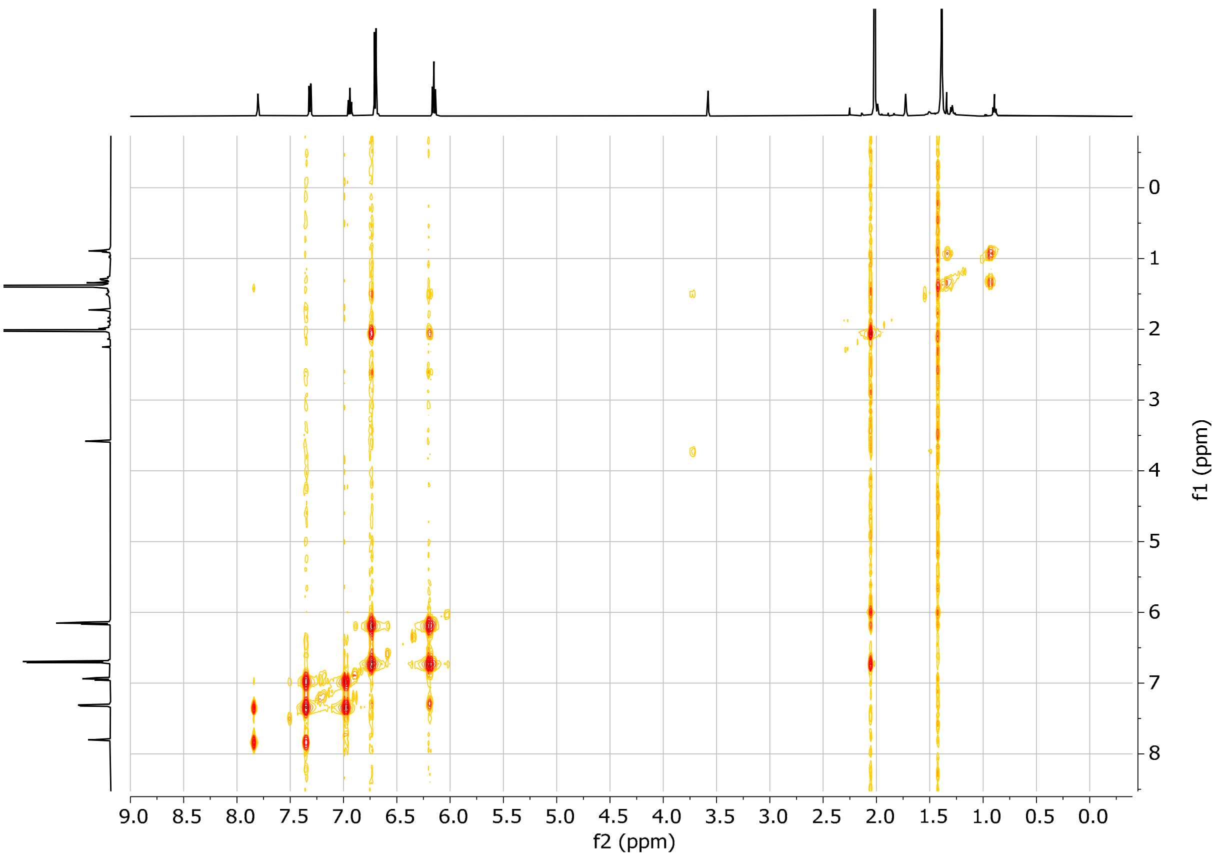

Figure S61. $^{1}H^{1}H$ COSY NMR spectrum of **5Xyl-HLi$_2$** in thf-d_8.

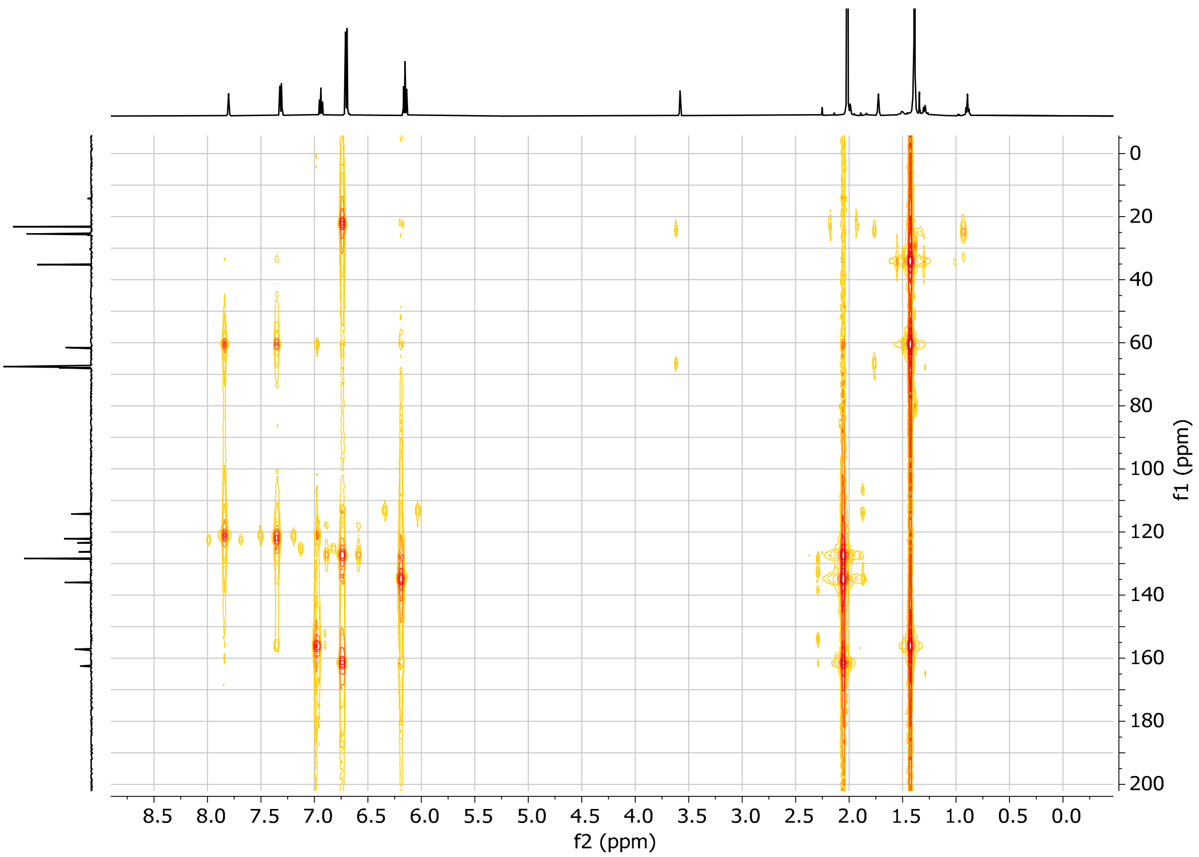

Figure S62. $^{1}H^{13}C$ HMBC NMR spectrum of **5Xyl-HLi$_2$** in thf-d_8.

Computational details

Cartesian coordinates for **1TMS-Bi**

Theory: PBE1PBE-GD3BJ

Basis Set: Def2-SVP

Bi	0.00000	-0.84284	-0.00000
Si	3.42097	-1.40854	-0.24929
Si	-3.42097	-1.40854	0.24929
N	-0.00000	1.33451	-0.00000
N	-2.17424	-0.19546	0.09709
N	2.17424	-0.19546	-0.09709
C	3.63065	1.74148	0.21959
H	4.51902	1.13627	0.09329
C	-1.36720	3.29525	-0.60222
H	-0.49584	3.88724	-0.83814
C	-1.20800	1.96025	-0.19482
C	-3.63065	1.74148	-0.21959
H	-4.51902	1.13626	-0.09329

C	1.20800	1.96025	0.19482
C	-2.61688	3.84324	-0.77293
H	-2.71043	4.87097	-1.10243
C	-2.36641	1.12898	-0.08546
C	1.36719	3.29525	0.60222
H	0.49583	3.88724	0.83814
C	-3.75703	3.06990	-0.54146
H	-4.74313	3.50632	-0.65103
C	2.61688	3.84324	0.77294
H	2.71042	4.87097	1.10243
C	3.75703	3.06990	0.54146
H	4.74313	3.50632	0.65103
C	2.36641	1.12898	0.08546
C	2.56668	-3.06694	-0.45088
H	1.96198	-3.34135	0.41797
H	3.33347	-3.83944	-0.56093
H	1.93547	-3.11092	-1.34224
C	-4.48853	-1.51064	-1.28666
H	-5.05281	-0.59565	-1.47420
H	-5.20376	-2.33334	-1.19586
H	-3.86695	-1.70142	-2.16548
C	4.46713	-1.11256	-1.77333
H	3.83703	-1.09874	-2.66633
H	5.20233	-1.91387	-1.89116
H	5.00778	-0.16507	-1.73590
C	-2.56668	-3.06694	0.45087
H	-1.96199	-3.34135	-0.41799
H	-3.33347	-3.83944	0.56093
H	-1.93546	-3.11093	1.34223
C	-4.46711	-1.11256	1.77335
H	-3.83701	-1.09878	2.66635
H	-5.20235	-1.91384	1.89116
H	-5.00773	-0.16506	1.73594
C	4.48852	-1.51065	1.28667
H	5.05275	-0.59563	1.47425

H	5.20378	-2.33331	1.19585
H	3.86693	-1.70148	2.16548

Cartesian coordinates for **3TMS-Bi**

Theory: PBE1PBE-GD3BJ

Basis Set: Def2-SVP

C	-1.22503	2.35198	0.00005
C	-1.21828	3.74862	-0.00008
C	-0.00011	4.43282	-0.00021
C	1.21811	3.74870	-0.00018
C	1.22496	2.35205	-0.00001
C	-0.00002	1.68937	0.00008
C	2.47733	1.50729	0.00024
C	-2.47737	1.50713	0.00015
N	2.20079	0.09777	-0.00013
N	-2.20076	0.09764	0.00038
Bi	0.00005	-0.49532	0.00021
Si	3.53092	-1.03416	-0.00020
C	4.60625	-0.80559	1.53178
C	2.82919	-2.78081	-0.00160
C	4.60788	-0.80374	-1.53073
C	-4.60922	-0.80299	1.52936
C	-2.82931	-2.78093	0.00267
C	-4.60491	-0.80622	-1.53312
Si	-3.53093	-1.03425	-0.00011
H	-2.15880	4.30802	-0.00010
H	-0.00014	5.52597	-0.00035
H	2.15859	4.30816	-0.00028
H	3.08780	1.80765	0.87948
H	3.08868	1.80801	-0.87825
H	-3.08821	1.80758	-0.87880
H	-3.08826	1.80781	0.87897
H	5.42840	-1.53899	1.55352
H	4.01102	-0.93217	2.44992
H	5.05798	0.19886	1.55978

H	3.65408	-3.51132	-0.00137
H	2.21513	-2.97945	-0.89451
H	2.21388	-2.98037	0.89024
H	5.42825	-1.53909	-1.55399
H	5.06205	0.19969	-1.55557
H	4.01298	-0.92625	-2.44963
H	-5.43017	-1.53773	1.55161
H	-5.06270	0.20077	1.55370
H	-4.01549	-0.92604	2.44894
H	-3.65423	-3.51141	0.00210
H	-2.21593	-2.97918	0.89614
H	-2.21330	-2.98092	-0.88860
H	-5.42659	-1.54012	-1.55588
H	-4.00863	-0.93224	-2.45065
H	-5.05721	0.19797	-1.56139

Cartesian coordinates for **5TMS-Bi**

Theory: PBE1PBE-GD3BJ

Basis Set: Def2-SVP

C	-1.21563	2.18449	0.07214
C	-1.19774	3.58215	0.09333
C	0.01725	4.26261	0.00044
C	1.22862	3.57562	-0.09223
C	1.23653	2.17777	-0.10120
C	0.00685	1.52575	-0.04022
C	2.51841	1.34175	-0.19021
C	-2.49386	1.35458	0.23856
N	2.20782	-0.07612	-0.10140
N	-2.20001	-0.06294	0.10797
Bi	-0.00309	-0.64956	-0.09383
C	-3.02897	1.65727	1.65485
C	-3.52418	1.81006	-0.80842
C	3.15999	1.64947	-1.56025
C	3.46899	1.78522	0.93444
Si	3.37745	-1.35023	0.17875

C	3.87721	-1.49377	1.99570
C	2.56909	-2.99368	-0.28447
C	4.95634	-1.22135	-0.84890
C	-4.88781	-1.19411	1.08805
C	-2.56405	-2.97837	0.33379
C	-4.04557	-1.46979	-1.83402
Si	-3.39797	-1.33071	-0.06401
H	-2.12416	4.15485	0.18663
H	0.02107	5.35563	0.01132
H	2.16086	4.14351	-0.15011
H	-3.97338	1.12723	1.83455
H	-3.20535	2.73439	1.79421
H	-2.29553	1.32615	2.40421
H	-3.16006	1.58624	-1.82140
H	-3.71705	2.89047	-0.73932
H	-4.48402	1.29326	-0.65874
H	2.48283	1.32901	-2.36505
H	4.11212	1.11477	-1.67353
H	3.35308	2.72663	-1.67643
H	3.02633	1.55985	1.91535
H	3.67635	2.86403	0.88722
H	4.43269	1.25990	0.85577
H	4.47976	-2.40282	2.15665
H	2.98246	-1.56285	2.63480
H	4.46866	-0.63313	2.34135
H	3.32229	-3.79347	-0.19511
H	2.20508	-2.99578	-1.32422
H	1.73031	-3.26957	0.37400
H	5.58094	-2.10980	-0.65916
H	5.56091	-0.33654	-0.59713
H	4.73563	-1.18932	-1.92716
H	-5.52947	-2.07981	0.94876
H	-5.50695	-0.30667	0.88571
H	-4.58164	-1.16387	2.14526
H	-3.32494	-3.77539	0.30663

H	-2.11761	-2.98050	1.34100
H	-1.78204	-3.25750	-0.38983
H	-4.67249	-2.36988	-1.94399
H	-3.20642	-1.55218	-2.54310
H	-4.65029	-0.60060	-2.13213

Cartesian coordinates for **3Xyl-Bi**

Theory: PBE1PBE-GD3BJ

Basis Set: Def2-SVP

C	-1.22396	2.55221	-0.04273
C	-1.21640	3.94874	-0.04321
C	0.00000	4.63368	-0.00032
C	1.21640	3.94874	0.04266
C	1.22396	2.55221	0.04243
C	-0.00000	1.88493	-0.00008
C	2.47770	1.71919	0.08487
C	-2.47771	1.71920	-0.08509
N	2.18247	0.31443	0.08478
N	-2.18249	0.31444	-0.08476
C	3.28012	-0.56261	0.08745
Bi	-0.00002	-0.30465	0.00012
C	-3.28011	-0.56263	-0.08745
C	4.01423	-0.80375	-1.09774
C	5.07763	-1.71041	-1.06542
C	5.42601	-2.37062	0.10783
C	4.70049	-2.13145	1.27100
C	3.62692	-1.23780	1.28073
C	3.65158	-0.11478	-2.38048
C	2.85254	-0.99921	2.54559
C	-4.01446	-0.80350	1.09765
C	-5.07779	-1.71023	1.06535
C	-5.42588	-2.37079	-0.10780
C	-4.70013	-2.13188	-1.27088
C	-3.62662	-1.23815	-1.28062
C	-3.65217	-0.11412	2.38027

C	-2.85202	-0.99986	-2.54541
H	-2.15681	4.50689	-0.07658
H	0.00001	5.72659	-0.00042
H	2.15682	4.50689	0.07593
H	3.06745	1.99948	0.98432
H	3.12409	2.00525	-0.77111
H	-3.06742	1.99934	-0.98461
H	-3.12414	2.00539	0.77082
H	5.63661	-1.90304	-1.98566
H	6.26125	-3.07480	0.11566
H	4.97161	-2.64507	2.19766
H	2.56884	0.07033	-2.43710
H	3.95498	-0.71396	-3.25067
H	4.14951	0.86519	-2.47316
H	3.40432	-1.37523	3.41859
H	1.87837	-1.51650	2.53092
H	2.64006	0.06886	2.69863
H	-5.63697	-1.90265	1.98551
H	-6.26107	-3.07502	-0.11562
H	-4.97102	-2.64575	-2.19746
H	-2.56947	0.07122	2.43702
H	-3.95559	-0.71313	3.25057
H	-4.15032	0.86577	2.47262
H	-3.40395	-1.37551	-3.41848
H	-1.87814	-1.51770	-2.53073
H	-2.63896	0.06811	-2.69833

Cartesian coordinates for **5Xyl-Bi**

Theory: PBE1PBE-GD3BJ

Basis Set: Def2-SVP

C	-1.22824	2.40434	0.00001
C	-1.21583	3.80211	0.00041
C	-0.00008	4.48674	0.00062
C	1.21573	3.80220	0.00050
C	1.22825	2.40443	0.00007

C	0.00003	1.74318	-0.00031
C	2.52037	1.58842	0.00007
C	-2.52031	1.58822	-0.00007
N	2.17446	0.16912	-0.00010
N	-2.17424	0.16890	-0.00001
C	3.18803	-0.80769	0.00021
Bi	0.00012	-0.44337	-0.00067
C	-3.18798	-0.80778	0.00025
C	-3.34158	1.96857	1.24580
C	-3.34114	1.96835	-1.24632
C	3.34135	1.96873	-1.24605
C	3.34147	1.96865	1.24609
C	3.66532	-1.35369	-1.22241
C	4.66222	-2.33354	-1.19656
C	5.17952	-2.81086	0.00088
C	4.66173	-2.33323	1.19796
C	3.66477	-1.35341	1.22313
C	3.10964	-0.94776	-2.55845
C	3.10835	-0.94728	2.55880
C	-3.66496	-1.35332	1.22316
C	-4.66230	-2.33276	1.19795
C	-5.18029	-2.81011	0.00084
C	-4.66278	-2.33297	-1.19658
C	-3.66540	-1.35361	-1.22238
C	-3.10842	-0.94743	2.55887
C	-3.10924	-0.94817	-2.55838
H	-2.14961	4.37025	0.00061
H	-0.00012	5.57964	0.00097
H	2.14945	4.37042	0.00081
H	-4.25962	1.36446	1.29384
H	-3.63368	3.02789	1.20609
H	-2.75883	1.80981	2.16220
H	-2.75820	1.80899	-2.16249
H	-3.63284	3.02780	-1.20707
H	-4.25938	1.36452	-1.29435

H	2.75844	1.80949	-2.16227
H	4.25954	1.36482	-1.29405
H	3.63310	3.02815	-1.20664
H	2.75863	1.80968	2.16238
H	3.63354	3.02799	1.20657
H	4.25954	1.36455	1.29413
H	5.02447	-2.73948	-2.14553
H	5.95896	-3.57638	0.00113
H	5.02360	-2.73890	2.14720
H	3.77800	-0.24801	-3.08488
H	2.13702	-0.45054	-2.45835
H	2.98948	-1.82893	-3.20613
H	3.77723	-0.24878	3.08623
H	2.98621	-1.82859	3.20595
H	2.13655	-0.44864	2.45789
H	-5.02431	-2.73833	2.14717
H	-5.96009	-3.57527	0.00105
H	-5.02522	-2.73870	-2.14557
H	-2.13642	-0.44917	2.45803
H	-2.98673	-1.82880	3.20600
H	-3.77705	-0.24864	3.08624
H	-2.98754	-1.82981	-3.20516
H	-2.13725	-0.44981	-2.45797
H	-3.77805	-0.24971	-3.08595

Cartesian coordinates for **3Xyl-HLi$_2$**

Theory: PBE1PBE-GD3BJ

Basis Set: Def2-SVP

Solvent Model: SCRF=(Solvent=TetraHydroFuran)

C	1.31110	2.64812	-0.67849
C	1.58635	3.45424	0.43423
C	0.53198	4.00001	1.17515
C	-0.78878	3.61588	0.91163
C	-1.06875	2.82345	-0.20629

C	-0.02029	2.51920	-1.07493
C	-2.26346	1.91737	-0.36017
C	2.21052	1.55720	-1.20455
N	-1.72849	0.57247	-0.09253
N	1.60268	0.30120	-0.72850
C	-2.61261	-0.44980	0.20119
C	2.47271	-0.74462	-0.43139
C	-2.18188	-1.44710	1.13504
C	-3.00960	-2.51519	1.47850
C	-4.28428	-2.65231	0.93309
C	-4.70390	-1.71012	-0.00097
C	-3.90079	-0.63414	-0.39482
C	-0.84216	-1.32583	1.80202
C	-4.38959	0.21742	-1.53590
C	2.23206	-2.03091	-1.00072
C	3.04946	-3.11833	-0.68370
C	4.12165	-2.98671	0.19409
C	4.35217	-1.74488	0.78023
C	3.55191	-0.63372	0.49965
C	1.10116	-2.22921	-1.96971
C	3.79291	0.63134	1.27292
Li	-0.26918	-0.10362	-1.42189
Li	0.15399	0.75614	0.73954
H	2.61857	3.60134	0.76223
H	0.74894	4.63937	2.03480
H	-1.58818	3.88008	1.60940
H	-0.24390	1.96969	-1.99600
H	-2.64724	-3.24613	2.20816
H	-4.93017	-3.48613	1.21727
H	-5.68354	-1.82327	-0.47677
H	-0.76003	-0.37252	2.35585
H	0.01099	-1.40182	1.10231

H	-0.69321	-2.12849	2.53709
H	-5.27269	-0.24511	-1.99947
H	-3.61809	0.31699	-2.31689
H	-4.67350	1.23907	-1.24039
H	2.84334	-4.08678	-1.15008
H	4.75876	-3.84228	0.43020
H	5.16350	-1.63302	1.50683
H	1.20623	-3.17854	-2.51318
H	1.06975	-1.42193	-2.72079
H	0.11538	-2.29203	-1.46873
H	2.84594	1.15472	1.47732
H	4.43818	1.35081	0.74145
H	4.28305	0.41032	2.23228
H	2.24913	1.59253	-2.31502
H	3.24766	1.70916	-0.86577
H	-3.05975	2.19354	0.35732
H	-2.69035	2.05890	-1.37174

Cartesian coordinates for **5Xyl-HLi$_2$**

Theory: PBE1PBE-GD3BJ

Basis Set: Def2-SVP

Solvent Model: SCRF=(Solvent=TetraHydroFuran)

C	-1.26635	2.42010	0.21442
C	-1.36090	3.17578	-0.96186
C	-0.19434	3.62263	-1.59180
C	1.06648	3.21038	-1.14566
C	1.17100	2.46175	0.03382
C	0.00346	2.25207	0.76846
C	2.33668	1.55479	0.43602
C	-2.32093	1.44907	0.75084
N	1.71839	0.20047	0.28224
N	-1.68412	0.13020	0.43602

C	2.57609	-0.87036	-0.02839
C	-2.53827	-0.92707	0.07201
C	-2.53090	1.66973	2.26064
C	-3.68465	1.67304	0.09352
C	3.54997	1.76445	-0.47142
C	2.78341	1.90368	1.86733
C	2.68964	-1.31840	-1.37903
C	3.51329	-2.39744	-1.70959
C	4.22977	-3.08422	-0.73291
C	4.09512	-2.69277	0.59320
C	3.28514	-1.61021	0.96141
C	1.88863	-0.67379	-2.47600
C	3.16084	-1.30220	2.42634
C	-2.96113	-1.89898	1.02399
C	-3.79323	-2.95618	0.64056
C	-4.22078	-3.10044	-0.67585
C	-3.77835	-2.18798	-1.62757
C	-2.93962	-1.12249	-1.28154
C	-2.46958	-1.85555	2.44330
C	-2.42332	-0.24797	-2.39238
H	-2.33169	3.37662	-1.41924
H	-0.27177	4.22743	-2.49940
H	1.95120	3.43483	-1.74467
H	-3.32621	1.00691	2.63207
H	-2.83473	2.70822	2.47225
H	-1.62150	1.45721	2.84223
H	-3.65692	1.58106	-0.99885
H	-4.06522	2.67598	0.34135
H	-4.40866	0.93460	0.46668
H	3.31805	1.57639	-1.52823
H	4.35583	1.07390	-0.18379
H	3.93560	2.79172	-0.38053

H	3.65867	1.30795	2.15751
H	1.98889	1.71360	2.60411
H	3.06278	2.96781	1.93686
H	3.58804	-2.70717	-2.75657
H	4.87139	-3.92686	-1.00263
H	4.62319	-3.24503	1.37727
H	2.30483	-0.91589	-3.46420
H	1.84766	0.42097	-2.38422
H	0.85023	-1.05231	-2.48452
H	2.26437	-0.70413	2.63100
H	4.01945	-0.72345	2.80334
H	3.11709	-2.22935	3.01781
H	-4.10926	-3.68165	1.39672
H	-4.88098	-3.92412	-0.95910
H	-4.08083	-2.30506	-2.67306
H	-2.45118	-0.83839	2.84916
H	-1.44186	-2.25419	2.51453
H	-3.09241	-2.48312	3.09684
H	-3.17748	-0.11326	-3.18177
H	-1.54495	-0.71240	-2.87672
H	-2.12035	0.74574	-2.03989
H	0.08664	1.73752	1.72541
Li	0.07394	-0.35309	1.41422
Li	-0.03091	0.45138	-0.74317

Cartesian coordinates for **[3Xyl-Li$_2$]$^-$**

Theory: PBE1PBE-GD3BJ

Basis Set: Def2-SVP

Solvent Model: SCRF=(Solvent=TetraHydroFuran)

C	-1.26782	2.27725	0.14631
C	-1.24554	3.66098	0.38730
C	-0.03345	4.34446	0.45358

C	1.16056	3.64844	0.26425
C	1.14011	2.27218	-0.00720
C	-0.07518	1.54604	-0.07274
C	2.47390	1.58337	-0.27910
C	-2.63258	1.58899	0.17591
N	2.49000	0.20156	0.13074
N	-2.65588	0.33043	-0.52009
C	3.60780	-0.54820	0.11709
C	-3.63160	-0.57643	-0.31517
C	4.89287	-0.25372	-0.48319
C	5.95976	-1.14026	-0.30875
C	5.86001	-2.34259	0.39031
C	4.60808	-2.67836	0.90974
C	3.51335	-1.83352	0.77775
C	5.14672	0.92222	-1.39946
C	2.19171	-2.24585	1.34736
C	-4.95738	-0.34761	0.21341
C	-5.81132	-1.43053	0.44249
C	-5.47309	-2.74939	0.14197
C	-4.22786	-2.97765	-0.44864
C	-3.33297	-1.94123	-0.68612
C	-5.54125	1.03295	0.39415
C	-2.00300	-2.23625	-1.30727
H	-2.18536	4.20712	0.53532
H	-0.01768	5.42014	0.65555
H	2.11601	4.18361	0.32315
H	3.26276	2.17484	0.22994
H	2.67282	1.72395	-1.36360
H	-3.36861	2.33242	-0.19383
H	-2.89506	1.45499	1.25050
H	6.91689	-0.87472	-0.77254
H	6.72083	-3.00567	0.50259
H	4.47254	-3.62827	1.43910
H	4.47081	0.92151	-2.26932
H	6.17267	0.86421	-1.79332

H	5.04027	1.90470	-0.91814
H	2.25126	-3.22996	1.83404
H	1.41319	-2.32879	0.56273
H	1.83008	-1.52890	2.11037
H	-6.80698	-1.21538	0.84767
H	-6.16870	-3.56926	0.33552
H	-3.93400	-3.99591	-0.72716
H	-5.08162	1.62441	1.19960
H	-6.61549	0.95421	0.61956
H	-5.44565	1.63044	-0.52714
H	-1.87262	-3.31156	-1.49595
H	-1.16785	-1.91775	-0.65166
H	-1.87509	-1.72516	-2.28219
Li	-0.95788	0.13531	-1.33885
Li	0.73283	-0.21560	0.72249

Cartesian coordinates for **$[5Xyl\text{-}Li_2]^-$**

Theory: PBE1PBE-GD3BJ

Basis Set: Def2-SVP

Solvent Model: SCRF=(Solvent=TetraHydroFuran)

C	1.15147	2.02609	-0.16841
C	1.07031	3.42302	-0.32507
C	-0.14673	4.07814	-0.17679
C	-1.29966	3.35234	0.12932
C	-1.23580	1.96076	0.28732
C	-0.00188	1.26746	0.14231
C	-2.50788	1.15453	0.66061
C	2.52813	1.34779	-0.38723
N	-2.58541	-0.01444	-0.21810
N	2.48687	-0.03406	0.06906
C	-3.65112	-0.88684	-0.13422
C	3.61431	-0.82644	-0.01260
C	2.85968	1.47632	-1.89334
C	3.61297	2.14870	0.37043
C	-2.35553	0.75491	2.14940

C	-3.76867	2.03515	0.60082
C	-3.52562	-2.13077	0.56991
C	-4.58999	-3.03332	0.63971
C	-5.80268	-2.77963	0.00002
C	-5.91918	-1.62183	-0.76227
C	-4.87546	-0.69295	-0.85506
C	-2.21424	-2.51083	1.19606
C	-5.02853	0.46173	-1.80344
C	3.88963	-1.65097	-1.15544
C	5.01360	-2.48299	-1.19155
C	5.89536	-2.56789	-0.11767
C	5.61357	-1.83173	1.03013
C	4.50124	-0.98739	1.10602
C	2.93598	-1.69362	-2.31498
C	4.22967	-0.28288	2.40512
H	1.95754	4.01431	-0.57208
H	-0.20284	5.16453	-0.30193
H	-2.24152	3.89451	0.24036
H	3.82416	0.99208	-2.11696
H	2.92909	2.53103	-2.20526
H	2.08097	0.99367	-2.50108
H	3.35209	2.24212	1.43416
H	3.75904	3.16152	-0.03637
H	4.57831	1.62324	0.29623
H	-1.41498	0.20686	2.30059
H	-3.19481	0.11102	2.46234
H	-2.33782	1.64060	2.80667
H	-4.65516	1.41514	0.80311
H	-3.89509	2.49927	-0.38616
H	-3.74837	2.83650	1.35674
H	-4.45829	-3.96400	1.20235
H	-6.62986	-3.49149	0.06768
H	-6.84076	-1.43551	-1.32521
H	-2.27899	-3.49961	1.67470
H	-1.88423	-1.78113	1.94673

H	-1.41394	-2.55689	0.43760
H	-4.03669	0.88400	-2.01767
H	-5.64795	1.27475	-1.39171
H	-5.50595	0.13568	-2.74162
H	5.18792	-3.09424	-2.08416
H	6.77061	-3.22179	-0.16229
H	6.26881	-1.92014	1.90399
H	1.95492	-1.32166	-1.98979
H	2.83734	-2.71812	-2.70762
H	3.25783	-1.05424	-3.15285
H	4.46214	-0.93190	3.26433
H	3.17643	0.02752	2.44539
H	4.83327	0.63281	2.51739
Li	-0.91804	-0.21523	-1.03445
Li	0.88104	-0.44879	0.91482

References

[1] A. S. Bruker, A. Bruker, *Acta Cryst. A* **1990**, *46*, 467-473.
[2] G. Sheldrick, *Acta Crystallographica Section A* **2015**, *71*, 3-8.
[3] G. Sheldrick, *Acta Crystallographica Section C* **2015**, *71*, 3-8.
[4] O. V. Dolomanov, L. J. Bourhis, R. J. Gildea, J. A. K. Howard, H. Puschmann, *Journal of Applied Crystallography* **2009**, *42*, 339-341.
[5] K. Sato, T. Honma, S. Sugai, *Agricultural and Biological Chemistry* **1985**, *49*, 3563-3567.
[6] T. Hynes, J. D. Masuda, S. S. Chitnis, *ChemPlusChem* **2022**, *87*, e202200244.
[7] C.-Y. Ho, C.-W. Chan, L. He, *Angewandte Chemie International Edition* **2015**, *54*, 4512-4516.
[8] J. F. McGarrity, C. A. Ogle, *Journal of the American Chemical Society* **1985**, *107*, 1805-1810.
